# Using high-throughput multiple optical phenotyping to decipher the genetic architecture of maize drought tolerance

**DOI:** 10.1186/s13059-021-02377-0

**Published:** 2021-06-24

**Authors:** Xi Wu, Hui Feng, Di Wu, Shijuan Yan, Pei Zhang, Wenbin Wang, Jun Zhang, Junli Ye, Guoxin Dai, Yuan Fan, Weikun Li, Baoxing Song, Zedong Geng, Wanli Yang, Guoxin Chen, Feng Qin, William Terzaghi, Michelle Stitzer, Lin Li, Lizhong Xiong, Jianbing Yan, Edward Buckler, Wanneng Yang, Mingqiu Dai

**Affiliations:** 1 National Key Laboratory of Crop Genetic Improvement, National Center of Plant Gene Research, and Hubei Key Laboratory of Agricultural Bioinformatics, Huazhong Agricultural University, Wuhan, 430070 China; 2 Hubei Hongshan laboratory, Wuhan, 430070 China; 3 Guangdong Academy of Agricultural Sciences, Guangzhou, 510640 China; 4 School of Integrative Plant Sciences, Section of Plant Breeding and Genetics, Cornell University, Ithaca, NY 14850 USA; 5 State Key Laboratory of Plant Physiology and Biochemistry, College of Biological Sciences, China Agricultural University, Beijing, 100193 China; 6 Department of Biology, Wilkes University, Wilkes-Barre, PA 18766 USA; 7 Institute for Genomic Diversity, Cornell University, Ithaca, NY 14850 USA; 8 Agricultural Research Service, United States Department of Agriculture, Ithaca, NY 14850 USA

## Abstract

**Background:**

Drought threatens the food supply of the world population. Dissecting the dynamic responses of plants to drought will be beneficial for breeding drought-tolerant crops, as the genetic controls of these responses remain largely unknown.

**Results:**

Here we develop a high-throughput multiple optical phenotyping system to noninvasively phenotype 368 maize genotypes with or without drought stress over a course of 98 days, and collected multiple optical images, including color camera scanning, hyperspectral imaging, and X-ray computed tomography images. We develop high-throughput analysis pipelines to extract image-based traits (i-traits). Of these i-traits, 10,080 were effective and heritable indicators of maize external and internal drought responses. An i-trait-based genome-wide association study reveals 4322 significant locus-trait associations, representing 1529 quantitative trait loci (QTLs) and 2318 candidate genes, many that co-localize with previously reported maize drought responsive QTLs. Expression QTL (eQTL) analysis uncovers many local and distant regulatory variants that control the expression of the candidate genes. We use genetic mutation analysis to validate two new genes, *ZmcPGM2* and *ZmFAB1A*, which regulate i-traits and drought tolerance. Moreover, the value of the candidate genes as drought-tolerant genetic markers is revealed by genome selection analysis, and 15 i-traits are identified as potential markers for maize drought tolerance breeding.

**Conclusion:**

Our study demonstrates that combining high-throughput multiple optical phenotyping and GWAS is a novel and effective approach to dissect the genetic architecture of complex traits and clone drought-tolerance associated genes.

**Supplementary Information:**

The online version contains supplementary material available at 10.1186/s13059-021-02377-0.

## Introduction

Maize (*Zea mays*), with more than one billion tons annual production (http://www.fao.org/wsfs/world-summit/en) [1], is a world major crop and an important resource for human food, animal feed, and bioenergy. However, drought, which has increased with global climate warming and increased world population (more demands for fresh water), poses serious threats to maize production worldwide [2–5]. Therefore, there are urgent demands and great interest in generating drought-tolerant maize cultivars through biotechnological approaches to ensure global food security and for sustainable development of agriculture.

Plant drought tolerance is a complex trait that is controlled by multiple quantitative trait loci (QTLs) with small effects [6, 7]. So far, hundreds of QTLs linked to maize plant height, biomass, and anthesis-silk intervals have been detected in drought experiments [8, 9]. Based on 368 natural inbred lines, several drought-tolerant genes that control seedling survival rates after drought stress have been cloned through association mapping [10–13]. Most of the phenotypes in these drought studies have been measured at particular maize developmental stages or given times. There are numerous dynamic molecular and physiological responses of plants under lasting drought. How these responses are genetically controlled remains elusive.

The major challenge for dynamic drought study is the “phenotyping bottleneck,” owing to the lower-throughput, costly, and labor-intensive process of conventional crop phenotyping [14, 15]. In recent years, high-throughput non-destructive plant phenotyping techniques are developing rapidly [16] and have been popularized in various crop populations to dissect the genetic basis of complex quantitative traits under abiotic stresses, such as phosphate deficiency tolerance of *Brassica napus* [17], drought response of wheat [18], salinity tolerance of rice [19], and drought resistance of rice [20]. Most of these studies have been focused on the external responses, for example, the morphology, biomass, and greenness-related traits. The internal responses of plant to drought are largely unknown. Genome-wide association studies (GWAS), which are based on linkage disequilibrium (LD), have been widely applied in genetic dissection of various agronomic traits of crops [21–24]. So far, combining both high-throughput phenotyping and GWAS has not been applied to reveal the genetic architecture of maize drought response.

In this study, we applied drought stresses to a maize association panel consisting of 368 genotypes over a course of 98 days. Many dynamic multiple optical image-based traits (hereafter referred as i-traits) were detected via a high-throughput multiple optical phenotyping system and the high-throughput image analysis pipelines we developed. These i-traits were collected non-destructively by multiple optical imaging, including RGB imaging, hyperspectral imaging (HSI), and X-ray computed tomography (CT), thus reflected the broad external (RGB i-traits) and internal (HSI and CT i-traits) responses of plant to drought. I-trait-based GWAS resulted in the identification of thousands of candidate genes. Gene-trait network indicated that there were distinct genetic controls of different types of i-traits. Many previously identified drought-tolerant genes were included in the candidate genes, and dozens of hotspot genes associated with multiple i-traits were identified. We further revealed the regulatory variants that control the candidate gene expression via eQTL analysis. In addition, we validated the roles of two new genes in the regulation of i-traits and maize drought tolerance through genetic mutation analyses. Furthermore, several i-traits associated well with survival rate and known drought-related spectral index were selected as potential markers for drought tolerance maize cultivar screening and breeding. The huge amounts of “genetic treasures” detected in our study indicate that combining high-throughput multiple optical phenotyping and GWAS is a powerful and promising approach to dissect the genetic architecture of complex crop traits and clone causal genes.

## Results

### Capture of large-scale i-traits in maize drought response

**Fig. 1:**
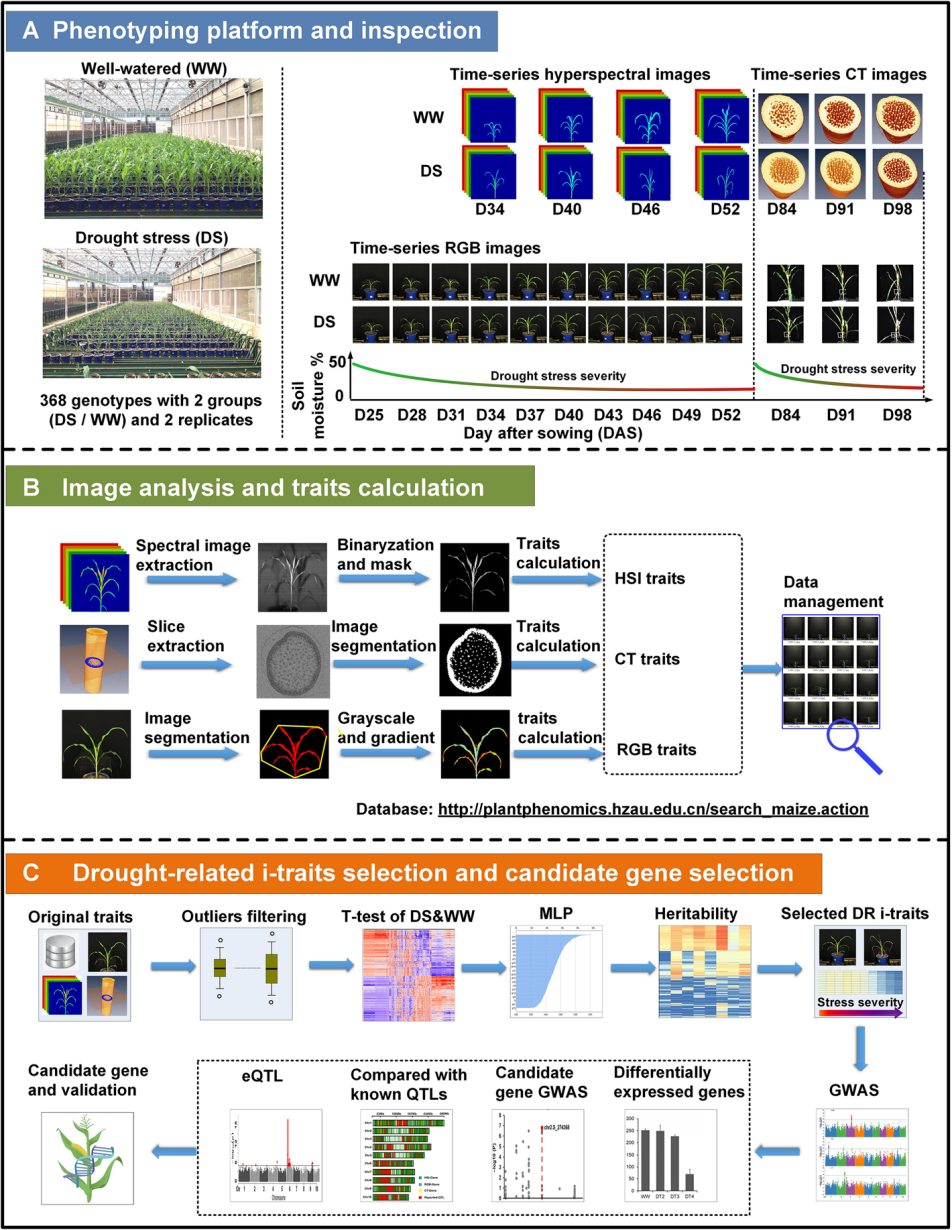
Combining high-throughput phenotyping and GWAS to study maize drought tolerance. **a** The phenotyping platform and experimental design. Left, the growth of the maize population under WW and DS conditions at D52 in greenhouse; middle and right, the capture of images with RGB, hyperspectral (HSI) and CT scanners under WW and DS conditions at different time point (D25-D98). **b** HSI, CT, and RGB image analyses and i-traits calculation with pipelines developed in this study. The details of these pipelines are shown in Additional file 9: Note S2 and Additional file 3: Video S2; Additional file 4: Video S3; Additional file 5: Video S4. All the images, phenotypic data, and genotype data are publicly available for reuse with the link: 10.6084/m9.figshare.14429003.v1. **c** A procedure showing the drought-related i-traits filtering and determining, GWAS, and candidate gene identification / validation


To gain insights into how maize plants respond to drought, we cultivated a maize association mapping population (AMP), which consists of 368 inbred lines and has 1 M SNPs among the population [25], in a greenhouse under well-watered (WW) and drought-stressed (DS) conditions (Additional file 1: Table S1; see “Methods”). By using an automatic platform for crop phenotyping developed based on our previous work (RAP [20, 26, 27]), the dynamic responses of each individual plant were captured in a noninvasive way via three types of scanners, RGB imaging, HSI, and CT, over a course of 98 days (Fig. 1a; Additional file 2: Video S1), which generated ~ 14 TB of images. To process the huge numbers of images, we further developed specific image analysis pipelines (Fig. 1b; Additional file 3: Video S2; Additional file 4: Video S3; Additional file 5: Video S4), with which a total of 26,910 i-traits (2010 RGB, 24,000 HSI, and 900 CT image-based traits) were extracted. After i-traits selection procedures (Fig. 1c), including filtering outliers, determination of drought-related i-traits using T-tests of WW/DS and multilayer perceptron (MLP), and heritability tests (Additional file 6: Video S5; Additional file 7: Video S6; Additional file 8: Video S7), 10,080 drought-related i-traits (37.46% of the rough i-traits, including 1503 RGB-derived, 7902 HSI-derived, and 675 CT-derived i-traits) were selected for further genetic study. The definitions of these i-traits are shown in Additional file 1: Table S2 and Additional file 9: Note S1. All the selected RGB, HSI, and CT i-traits are listed in Additional file 1: Table S3-5. All these images and related i-traits are open access to the public at 10.6084/m9.figshare.14429003.v1.

**Additional file 6: Video S5.** Operation procedure of filtering outlier.


**Additional file 7: Video S6.** Operation procedure of T-test.


**Additional file 8: Video S7.** Operation procedure of Multi-layer perception (MLP).

### Effective and inheritable i-traits to reflect maize drought response

**Fig. 2:**
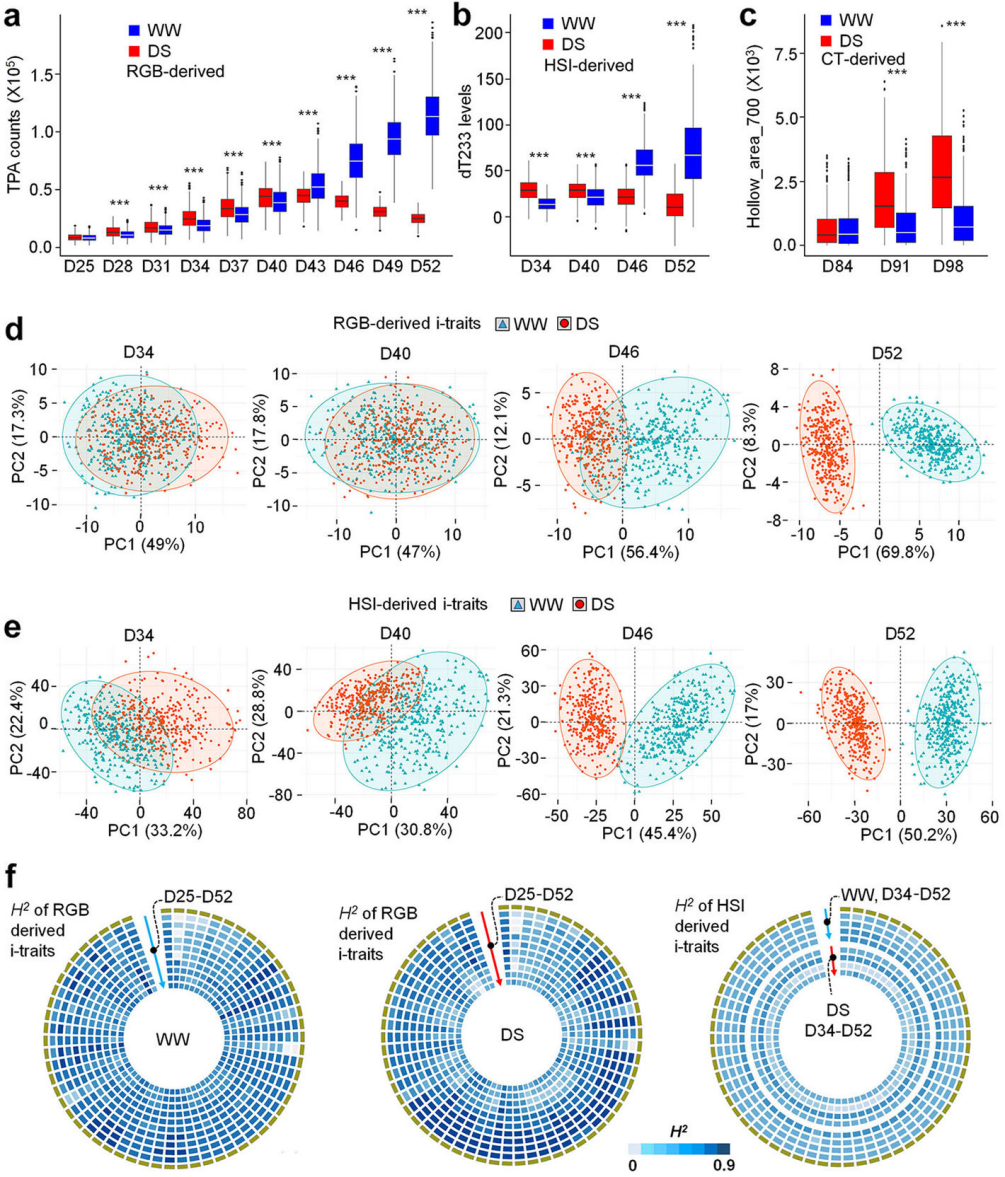
General analyses of i-traits. **a–c** Examples showing the levels of RGB-derived (TPA, **a**), HSI-derived (dT233, **b**), and CT-derived (Hollow_area_700, **c**) i-traits, which effectively indicated the levels of drought stress at different time points. PCA of RGB-derived (**d**) and HSI-derived (**e**) i-traits collected at time points D34, D40, D46, and D52. **f** Broad heritability (*H*^*2*^) of all RGB-derived and top 60 HSI-derived i-traits. More detailed *H*^*2*^ information is shown in Additional file 10: Figure S2. WW, well-watered; DS, drought-stressed; D, days after sowing

Many i-traits changed dynamically during the drought treatments and growth stages (Additional file 10: Figure S1a-c). For example, the RGB-derived i-trait TPA (total projected area), which has been reported as a good indicator of rice growth under drought stress [20], was indicative of different growth situations of maize plants under various drought stresses (Fig. 2a). The HSI-derived i-trait dT233, which is the first-order derivative of the total reflectance under 959 nm, has been reported to reflect internal water content [28]. We observed that dT233 increased under WW conditions and decreased under DS conditions, suggesting that it was an effective indicator of drought responses (Fig. 2b). The CT-derived i-trait hollow_area_700 reflected culm wall size also effectively indicated different levels of drought stress (Fig. 2c).

To further explore the potential of i-traits to reflect drought responses, principal components analysis (PCA) was performed to capture the phenotypic variations in the entire maize population. For the RGB- and HSI-derived i-traits, at D52 with more severe stress, PC1 alone explained more than 50% of the phenotypic variation, which clearly separated the WW plants from those undergoing DS (Fig. 2d, e). Interestingly, compared with RGB-derived and CT-derived traits, HSI-derived traits had better distinguishing ability even in early stress stages (Fig. 2d, e, Additional file 10: Figure S1d-f). Next, we calculated the broad-sense heritability (*H*^*2*^) of each individual i-trait over the growth period with or without drought stress, and the middle number of *H*^*2*^ of these i-traits was 0.4 (Fig. 2f, Additional file 10: Figure S2).

### Genetic basis of i-traits in maize drought response

We performed GWAS of 10,080 i-traits with a mixed linear model (MLM) to detect significant SNP-trait associations. More than 2989 (29.6% of 10,080) i-traits had at least one significant associated SNP (*P* ≤ 1.8 × 10^−6^). We identified a total of 4322 distinct significant SNPs associated with 2989 i-traits (Additional file 1: Table S6 and 7). More significant SNPs (2378, ~ 55%) were detected with ratio i-traits as compared to those with i-traits from WW (972, ~ 22.5%) or DS (849, ~ 19.6%) conditions alone (Additional file 10: Figure S3a, Additional file 1: Table S7). Each SNP explained 5.3–22.6% of the observed phenotypic variance of the i-traits. The SNPs associated with CT-derived i-traits explained more phenotypic variance on average as compared to those with RGB and HSI-derived i-traits (Additional file 10: Figure S3b and c), suggesting either less complex genetic architecture or highly enriched diversity, both quantitatively and qualitatively, of CT-derived i-traits. We mapped the significant SNPs onto the maize chromosomes at 200-kb intervals (100 kb upstream and downstream of the significant SNP), and the mapped intervals were defined as QTLs controlling maize drought tolerance. In total, 1529 QTLs were identified (Additional file 1: Table S8). Of these, 71.4% (1092/1529) were co-localized with previously reported QTLs (Additional file 1: Table S9) [9, 29–31].

**Fig. 3:**
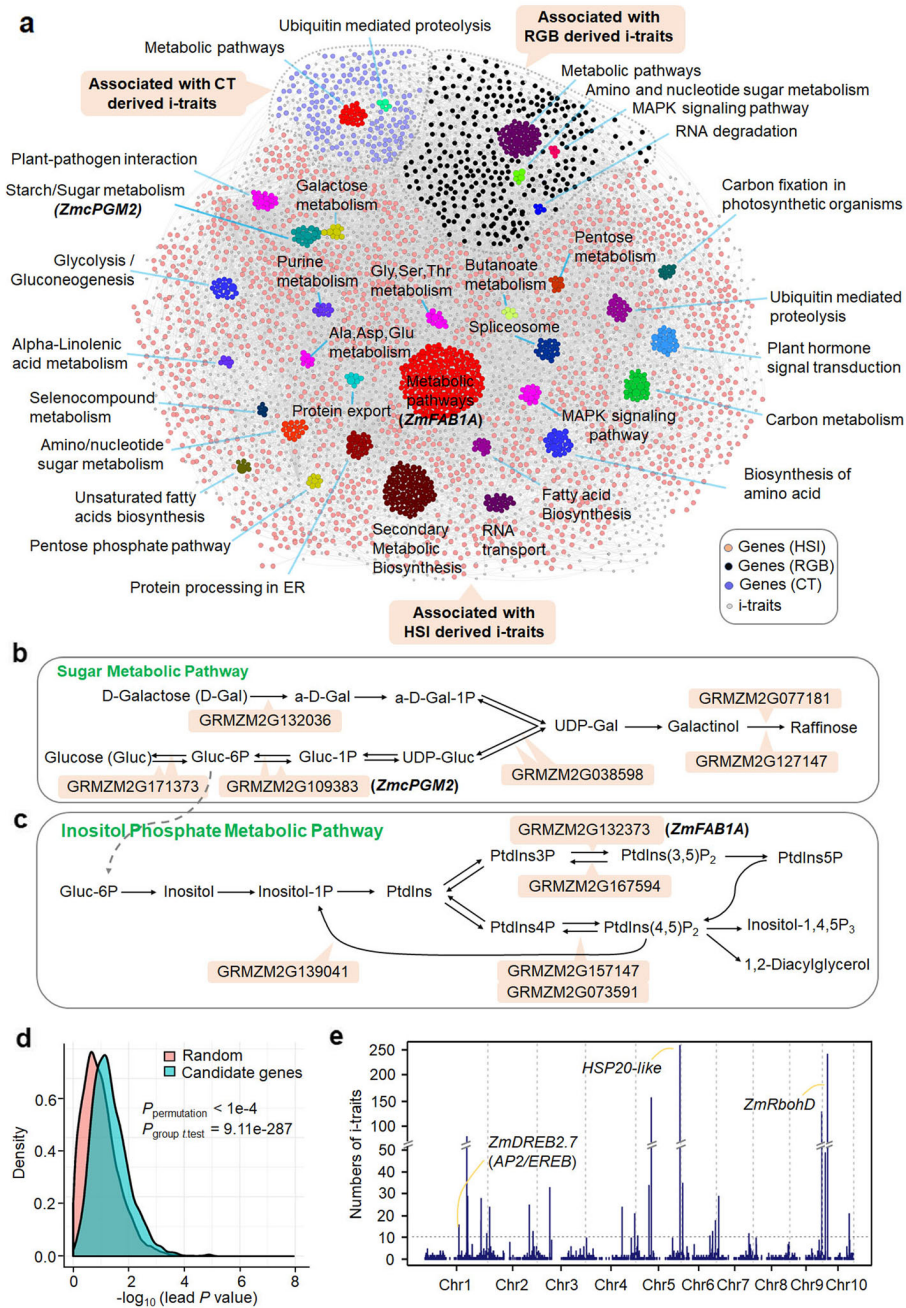
Associations from I-trait-based GWAS and analysis of the candidate genes. **a** Gene-trait network showing the distribution of candidate genes and the clustering of genes enriched in the same pathways. I-traits and their related network were shown in the bottom layer. Genes and their enriched pathways are shown in the upper layer. *ZmcPGM2* and *ZmFAB1A* were highlighted. **b** Genes enriched in the sugar metabolic pathway. *ZmcPGM2* that catalyzes the invertible step of Gluc-6P to Gluc-1P was highlighted. **c** Genes enriched in inositol phosphate metabolic pathway. *ZmFAB1A* that catalyzes the step of PtdIns3P to PtdIns(3,5)P_2_ was highlighted. **d** Density plot showing the P value distribution of most significant SNPs of the candidate genes and randomly selected genes. Ten thousand times of permutation tests with randomly selected genes were performed and compared to the candidate genes. **e** Number of i-traits associated with candidate genes

We extracted the candidate genes based on the significant SNPs, whose average LD decay in AMP used in this study have been reported to be 0.5 kb, reaching single-gene resolution [25]. In total, 2318 unique candidate genes related to 4322 significant associations were identified and annotated (Additional file 1: Table S7). Of which, only 95 genes (~ 4.1%) were consistently detected in two or more types of i-traits (Additional file 10: Figure S4a). Based on the genes and i-traits, we built a gene-trait network, in which the genes that involved in the same biological pathway were gathered in a group (Fig. 3a). This network would facilitate candidate gene identification and its function elucidation. We found that very few pathways were shared by genes associated with three types of i-traits, and many unique pathways were detected for genes associated with HSI-derived or RGB-derived i-traits (Fig. 3a, Additional file 1: Table S10 and Additional file 10: Figure S4a). For instance, although MAPK (mitogen-activated protein kinase) signaling and BR (Brassinolide) signaling pathways were shared by genes from HSI and RGB i-traits, several pathways, such as one carbon pool by folate, RNA degradation, and trypophan metabolism were unique to genes detected with RGB i-traits, and many other pathways, such as ABA signaling pathway, sugar metabolic pathway, and inositol phosphate metabolic pathway, were specific to genes associated with HSI-derived i-traits (Fig. 3b, c, Additional file 10: Figure S4b-d). These results indicated different genetic controls of these i-traits in drought responses. Further example of insights based on the data integration are shown below with case studies to *ZmcPGM2* and *ZmFAB1A* in regulation of i-traits and drought tolerance.

The candidate genes were significantly enriched in GO terms response to different stimuli or stresses, suggesting the importance of these candidate genes in maize drought/stress responses (Additional file 10: Figure S5, Additional file 1: Table S11). GWAS showed that many genes were significantly associated with previously published drought-tolerant phenotype survival rates (SR) of AMP [12] (MLM, Additional file 1: Table S1, Additional file 1: Table S12). Permutation assay showed that there were enriched most significant *P* values of these candidate genes as compared to those from randomly selected genes (*P*_*t*.test_ = 9.11e-287, *P*_permutation_ < 0.0001, Fig. 3d), suggesting that these associations are not false positive but real associations. Moreover, 25 previously identified drought-tolerant genes were detected in our candidate gene set (Additional file 1: Table S13). Taken together, these results indicated that the candidate genes were reliable and that the i-trait-based GWAS was powerful in mapping drought-responsive QTLs and causal genes.

Transcription factors (TFs) play key roles in plant drought tolerance [32]. In our GWAS results, there are 165 genes (7.1% or 165/2318) encoding TFs of 41 families, of which the NAC (14 genes) and AP2/EREB ERF (12 genes) TF families, which are well-known to control plant drought tolerance [32], were the families with the most members (Additional file 1: Table S14). The well-studied TF genes *ZmNAC111* (GRMZM2G127379), *Zmhdz10* (GRMZM2G041127), *ZmDREB2A* (GRMZM2G006745), and *ZmDREB2*.7 (GRMZM2G028386) [10, 11, 33, 34] were all detected by GWAS in this study. For example, *ZmNAC111* positively regulates maize drought tolerance [11]. The most significant SNP chr10.S_2681198 of *ZmNAC111* was significantly associated with the HSI-derived ratio i-trait ddT136_D46_R (the ratio of second-order derivative of the 725 nm total reflectance under drought stress to second-order derivative of the 725 nm total reflectance under well-water condition at 46 days after sowing) (*P* = 1.5 × 10^−6^, MLM) (Additional file 10: Figure S6a-d). There were two alleles of chr10.S_2681198. Plants with allele T had lower ddT136_D46_R levels (*P* = 1.39 × 10^−6^, *t*-test) but much higher (*P* = 2.65 × 10^−4^, *t*-test) *ZmNAC111* expression under DS (Additional file 10: Figure S6e and f), implying that allele T of chr10.S_2681198 could be a favorable allele in AMP for regulating maize drought tolerance by enhancing *ZmNAC111* expression. These analyses further suggested the reliability of the candidate drought-tolerant genes.

### Functional interpretation of hotspot candidate genes

Next, we identified hotspot candidate genes that associated with no less than 10 i-traits. In total, 34 hotspot genes were detected (Fig. 3e, Additional file 1: Table S15), of which 29 were associated with HSI-derived i-traits (85% or 29/34). The gene GRMZM2G028386 (*ZmDREB2.7*) was associated with 13 HSI-derived i-traits and encoded AP2/EREBP ERF TFs. *ZmDREB2.7* belongs to the AP2 DREB subfamily and positively regulates maize drought tolerance [10]. The most significant SNP chr1.s_201957847 in *ZmDREB2.7* was significantly associated with HSI-derived i-trait lgA15_D34_WW (the logarithm of the 434 nm average reflectance under well-water condition at 34 days after sowing) (*P* = 7.5 × 10^−7^, MLM) and 12 other i-traits (Additional file 1: Table S15, Additional file 10: Figure S7a-d). This most significant SNP showed high linkage (*R*^*2*^ > 0.93) with two other SNPs in the coding region and the reported drought-tolerant causal allele, five polymorphic sites in the promoter region (*R*^*2*^ = 1) [10] (Additional file 10: Figure S7d). Based on the A/T alleles of the most significant SNP, plants with allele T had higher levels of i-trait lgA15_D34_WW (*P* = 4.27 × 10^−8^, *t*-test) and higher survival rates (*P* = 8.10 × 10^−11^, *t*-test) after drought stress (Additional file 10: Figure S7e and f), suggesting that allele T is a favorable allele in regulation of lgA15_D34_WW levels and maize drought tolerance.

Reactive oxygen species (ROS) are important signaling molecules in stress responses [35]. The membrane protein respiratory burst oxidase homolog D (RbohD) triggers ROS signaling at the very early stage of dehydration (e.g., in ~ 20 min) and plays positive roles in stomatal closure and ABA signaling [36, 37]. HSP proteins play key roles in maintaining ROS homeostasis and further in plant drought tolerance [38, 39]. GRMZM2G098167 (HSP20-like protein) was associated with 258 HSI-derived ratio i-traits and GRMZM2G300965 (*ZmRbohD*) was associated with 241 HSI-derived ratio i-traits, and both genes shared 34 associated i-traits (Fig. 3e, Additional file 1: Table S4, Additional file 1: Table S15). Intriguingly, all of these i-traits were calculated from the HSI images captured at D34 (the first time point for HSI imaging) with SM = ~ 20% (Fig. 1a), which was at the early drought stress stage. Based on these data, we deduced that *ZmRbohD* could play a key role in initiating *ZmRbohD*-dependent ROS signaling initiation, and *HSP20-like* could function to maintain ROS signaling homeostasis in maize drought tolerance.

### Identification of the regulatory variants that control the candidate gene expression

**Fig. 4:**
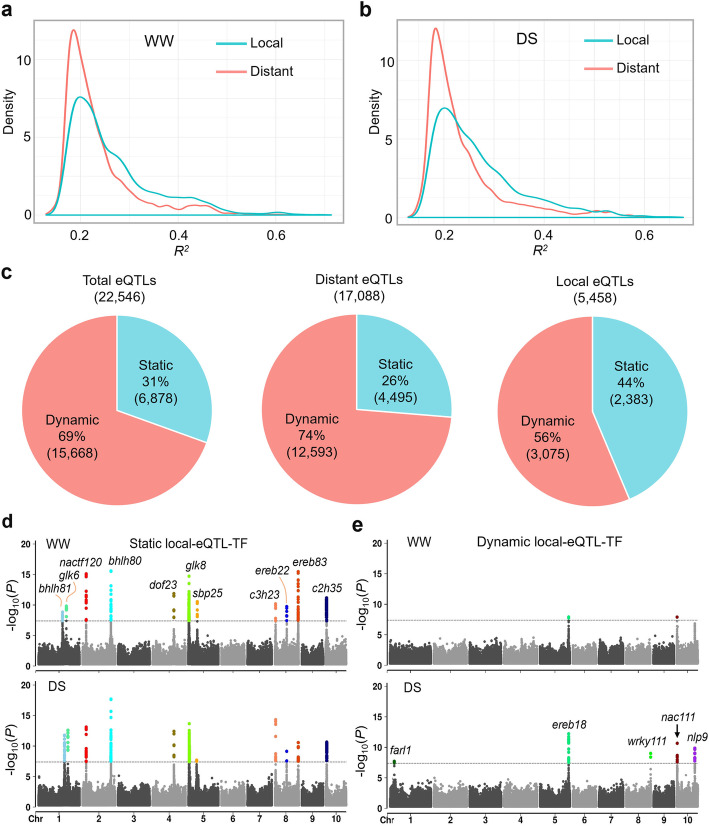
eQTLs that were associated with the expression of candidate genes. **a, b** Density plots showing the explained expression variance by significant local or distant eQTLs under WW (**a**) or DS (**b**) conditions. **c** The amounts and their percentages of static and dynamic eQTLs from total, distant, or local eQTL groups. **d** The local TF eQTLs that constantly detected under both WW and DS conditions. **e** The local TF eQTLs that specifically detected or enhanced significance under DS conditions

The difference in gene expression could originate from changes in local and/or distant regulation [40]. We next investigated the expression QTLs (eQTLs) that associated with the expression of the 2318 candidate genes based on the transcriptome of 197 lines from 540 association mapping population treated with or without drought (M. Dai and L. Li unpublished RNA-seq data) [41]. Totally, 54.2% (1257/2318) of the candidate genes were controlled by 22,546 significant eQTLs (*P* ≤ 4.2 × 10^−8^, MLM, Additional file 1: Table S16-18). When the most significant SNP of an eQTL was located in a 20-kb region (upstream of downstream) of the expression trait (etrait) gene, this eQTL was defined as a local eQTL; otherwise, it was a distant eQTL. We found that distant eQTLs were identified for most (~ 63%) candidate genes under both WW and DS conditions (Additional file 1: Table S18); however, the local eQTLs had much larger effects on the expression of the etrait genes under both WW and DS conditions (Fig. 4a, b), indicating that local variations have great effect on gene expression regulation.

Among the total eQTLs, the majority (69%, or 15,668/22,546) were dynamic (detected under WW or DS condition), only 31% of the eQTLs were static (detected under both WW and DS conditions). Similar ratios of dynamic (74%) and static (26%) eQTLs were observed in distant (17,088) eQTLs (Fig. 4c), indicating vast and dynamic gene regulatory networks in maize i-trait formation. We took closer look at the local eQTLs as they averagely have greater effects on gene expression regulation than distant eQTLs (Fig. 4a, b). Totally, 2383 static and 3075 dynamic local eQTLs were detected based on the gene expression under WW and DS conditions (Fig. 4c, Additional file 1: Table S18). For instance, very specific and significant eQTL peaks were constantly detected under WW and DS conditions for genes involved in IAA biosynthesis: GRMZM2G048295 (*myb15*), GRMZM2G163848 (*iap3*), GRMZM2G045404 (*ibr5*), sugar metabolism: GRMZM2G111324 (*ogh17*), GRMZM2G318780 (*scs3*), GRMZM2G171373 (*hk1*) and peroxide metabolism: GRMZM2G162688 (*sip*), GRMZM5G872256 (*gs1*). In addition, many significant peaks were repeatedly detected for genes encoding TFs that regulate multiple biological processes or stress responses (Fig. 4d). These data strongly indicated that the local regulatory variations have significant effects on the expression of its own. Dynamic significant peaks were detected under DS conditions for genes regulate BR biosynthesis: GRMZM2G472625 (*pk*), GRMZM2G012391 (*p450*), protein phosphorylation: GRMZM2G002100 (*mapk6*), GRMZM2G146553 (*cipk3*), heat stress response: GRMZM2G428391 (*hsp70*). Some significant peaks or enhanced significance of the peaks were detected for TF genes under DS conditions (Fig. 4e). Therefore, the local regulatory variants of these genes could be more specific for stress-responsive gene expression regulation in AMP.

### *ZmcPGM2* contributed to the diversity of HSI i-trait ddT200_R and maize drought tolerance via regulating the changes of sugar contents

To further interpret the findings from GWAS, we tested two genes *ZmcPGM2* (cytosolic phosphoglucomutase) and *ZmFAB1A* (1-phosphatidylinositol-4-phosphate 5-kinase or forms aploid and binucleate cells 1) which are annotated in sugar metabolic pathway and inositol phosphate metabolic pathway, respectively (Fig. 3b,c, Additional file 10: Figure S8a and b). In Arabidopsis, cPGM proteins regulate starch-dependent protein synthesis balance and are required for male and female gametophyte function [42, 43], but they have not been reported in regulation of plant drought tolerance.

**Fig. 5:**
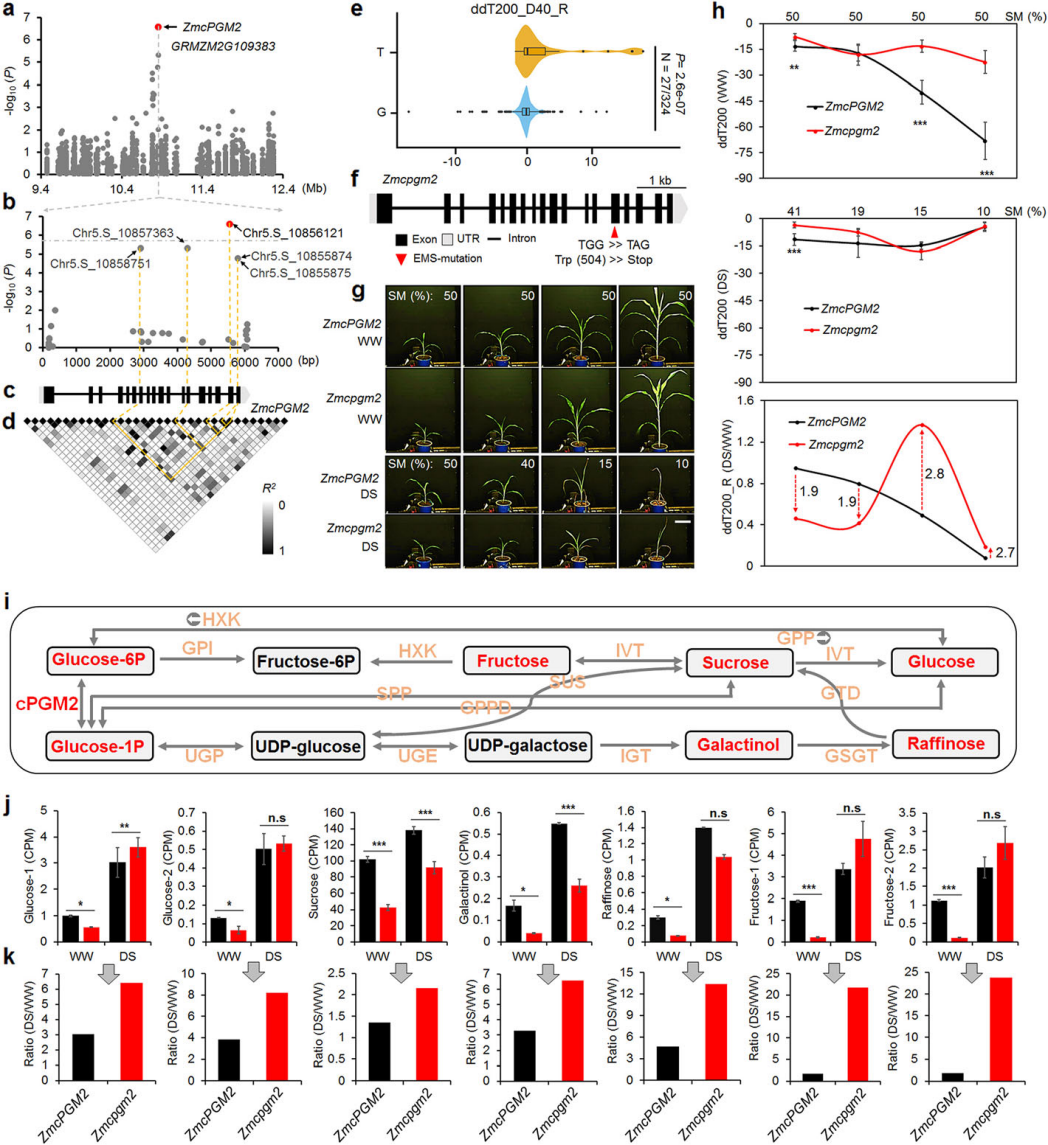
Roles of candidate gene *ZmcPGM2* in regulation of i-traits and sugar biosynthesis. **a** Zoom in on the view of Manhattan plot of chromosomal 5 region 9.4~12.4 Mb, where there were significant associations of SNPs with i-trait ddT200_D40_R. **b–d** Distribution of SNPs (**b**) in gene model *ZmPGM2* (**c**) and their LD to each other (**d**). The most significant SNP chr5.S_10856121 is highlighted with red dots in **b**. In panel **c**, filled black boxes indicate exons and black lines indicate introns of *ZmcPGM2*. **e** Plants with the T allele of chr5.S_10856121 showed significantly higher levels of i-trait ddT200_D40_R than those with the G allele in the AMP. **f** *ZmcPGM2* gene structure and position of the EMS mutation. **g** Growth of B73 wild type and *Zmcpgm2* mutant plants under WW and DS conditions. The soil moisture (SM) is shown on the top of the panels. Bar = 20 cm for all plants shown in this panel. **h** The levels of i-trait ddT200 in B73 wild type and *Zmcpgm2* mutant plants under WW, DS and ratio (DS/WW) conditions. The arrows and numbers show the fold decrease or increase in this trait in Zmcpgm2 mutants as compared to those in B73 wild type plants. **i** cPGM2 is responsible for reversibly converting glucose-1p to glucose-6p in sugar biosynthesis. Adapted and edited based on the KEGG database. Enzymes and their abbreviations: phosphoglucomutase (PGM), UTP-glucose-1-phosphate uridylyltransferase (UGP), UDP-glucose 4-epimerase (UGE), inositol 3-α-galactosyltransferase (IGT), galactinol-sucrose galactosyltransferase (GSGT), α-galactosidase (GTD), sucrose synthase (SUS), sucrose phosphorylase (SPP), glucose-1-phosphate phosphodismutase (GPPD), Hexokinase (HXK), glucose-6-phosphatase (GPP), glucose-6-phosphate isomerase (GPI), invertase (IVT). Arrows indicate the direction of the reaction. Sugars identified with GC-MS in this study are highlighted in red. **j** Sugar contents of B73 wild type and *Zmcpgm2* mutant plants grown under WW and DS conditions. **k** Fold increase in sugar contents (DS/WW) in B73 wild type and *Zmcpgm2* mutant plants. Statistical significance was determined by Student’s t-test: *P < 0.05; **P < 0.01; ***P < 0.001

The *ZmcPGM2* locus (GRMZM2G109383) showed significant (*P* = 2.57 × 10^−7^, MLM) association with i-trait ddT200_D40_R (the ratio of second-order derivative of the 880 nm total reflectance under drought stress to second-order derivative of the 880 nm total reflectance under well-water condition at 40 days after sowing) (Fig. 5a). The most significant SNP chr5.S_10856121, which explained 8.4% of the phenotypic variance (Additional file 1: Table S7), was located in the coding region of *ZmcPGM2* and had strong LD (*R*^2^ > 0.76) with four other less significant SNPs (*P* < 10^−4^) (Fig. 5b–d). There are two alleles of SNP chr5.S_10856121 and plants in the maize population with T allele had higher levels of ddT200_D40_R than plants with the G allele (Fig. 5e). A mutant *Zmcpgm2*, which had a stop mutation at Trp(504) of *ZmcPGM2* (Fig. 5f), was obtained from a maize EMS mutant bank [8]. *Zmcpgm2* plants were grown under WW and DS conditions and the HSI i-traits ddT200 were captured and calculated (Fig. 5g). We observed that the levels of ratio i-traits ddT200_R were lower in *Zmcpgm2* than those in B73 wild type (WT) plants when there was no stress, but the levels of this i-trait were higher in *Zmcpgm2* than those in WT plants when the stress was more severe (SM ≤ 15%) (Fig. 5 h), demonstrating a role of *ZmcPGM2* in regulation of i-trait ddT200_R.

cPGM reversibly converts glucose-1P to glucose-6P and plays important roles in regulation of sugar biosynthesis [42] (Fig. 5i). Previous studies showed that ddT200 reflects the cellular sugar contents [44]. We investigated the sugar contents of *Zmcpgm2* and WT plants treated with or without drought (Additional file 1: Table S19). Under WW conditions, the main sugars showed lower levels in *Zmcpgm2* than in WT plants, and drought promoted the levels of all these sugars in both *Zmcpgm2* and WT plants, but the changes in all these sugars (ratios of sugar contents under DS/WW conditions) were much higher in *Zmcpgm2* than in WT plants (Fig. 5j, k). These results demonstrated important roles of ZmcPGM2 in regulation of maize sugar contents and suggested the consistence of ddT200_R with the changes in sugar contents during maize drought responses.

*ZmcPGM2* was also significantly associated with CT i-trait Culm_diameter_700_D98_R (the ratio of stem thickness under DS/WW conditions), and the most significant SNPs were chr5.S_10857363 and chr5.S_10858751 (*P* = 3.46 × 10^−7^, MLM), which were completely linked to each other (*R*^*2*^ = 1) and highly linked with Chr5.S_10856121 (*R*^*2*^ = 0.81) (Additional file 10: Figure S8c-f). Plants with allele C had higher levels of Culm_diameter_700_D98_R than those of plants with allele A (from chr5.S_10857363) (Additional file 10: Figure S8g). Under WW conditions, the levels of i-trait Culm_diameter_700_D98_R in WT plants were higher than those in *Zmcpgm2* mutants, but after severe stress (SM = 15% or 10%), the levels of this i-trait in *Zmcpgm2* mutants were higher than those in WT plants (Additional file 10: Figure S8h-j). The ratios of these i-traits were larger in *Zmcpgm2* mutants than in WT plants under both WW and DS conditions (Additional file 10: Figure S8k). These results suggested a role *ZmcPGM2* in regulation of relatively higher (~ 10%) maize stem thickness.

**Fig. 6:**
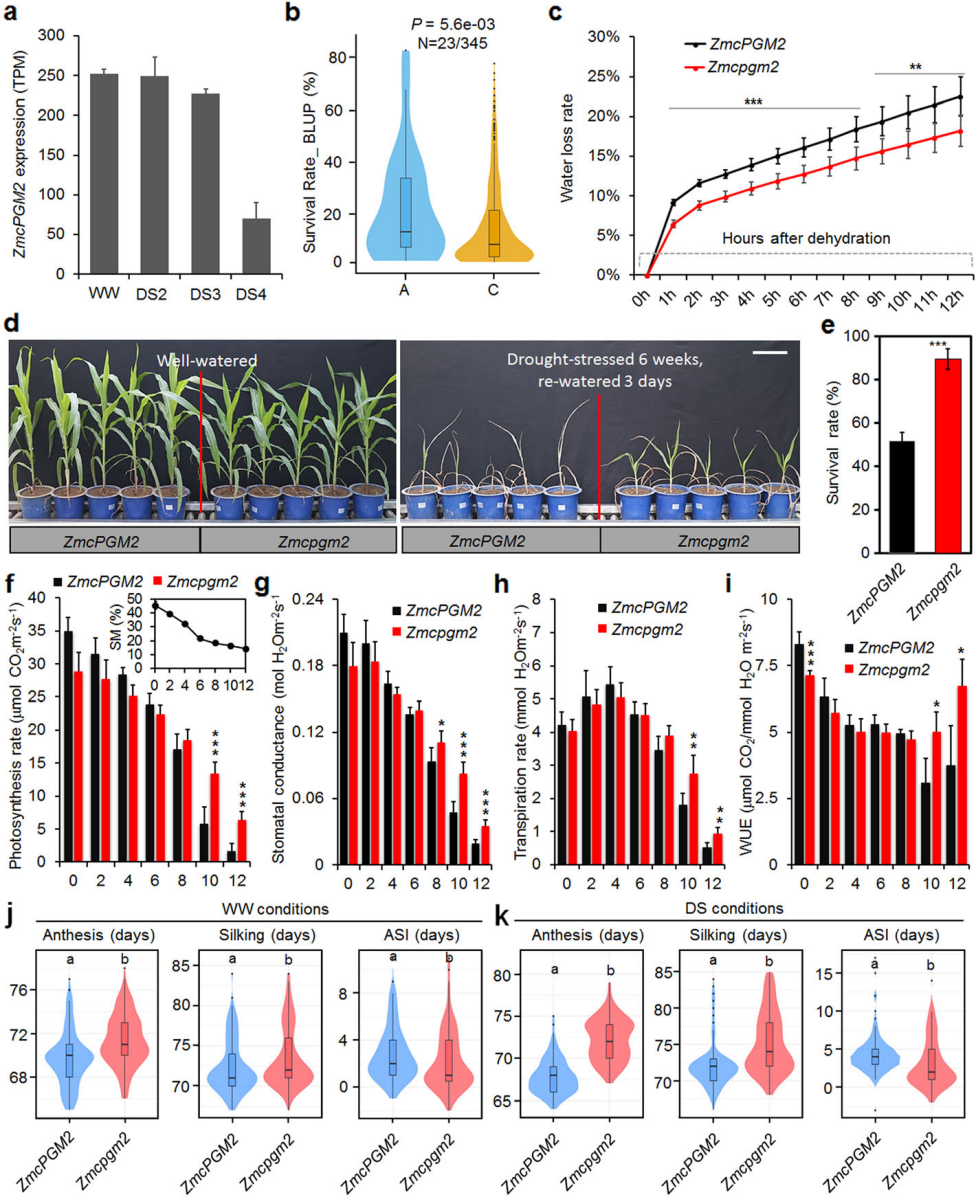
Roles of *ZmcPGM2* in regulation of maize drought tolerance. **a** Expression of *ZmcPGM2* in maize plants grown under WW or DS conditions. DS2-4 indicates different stress levels. **b** Plants with different alleles (A/C) of chr5.S_10857363, which showed high LD with chr5.S_10856121 (*R*^*2*^ = 0.81), showed significantly different survival rates in the maize population. **c** Comparison of water loss rate between detached leaves of B73 wild type and *Zmcpgm2* mutants. **d** Growth of B73 wild type and *Zmcpgm2* mutant plants under well-watered (WW) and drought-stressed (DS) conditions followed by re-watering. Bar = 20 cm for all plants shown in this panel. **e** Comparison of the survival rates of B73 wild type and *Zmcpgm2* mutant plants after drought stress. **f–i** Comparison of the photosynthetic rates (**f**), stomatal conductances (**g**), transpiration rates (**h**), and water use efficiencies (WUE, **i**) of B73 wild type and *Zmcpgm2* mutant plants after ceasing watering at different time points. Days indicate the time after irrigation ceased. The embedded graph in (**f**) indicates the soil moistures (SM) at each time point without irrigation. Statistical significance was determined by Student’s t-test: *P < 0.05; **P < 0.01; ***P < 0.001. **j, k** Anthesis-silking intervals (ASI) of B73 and *Zmcpgm2* mutant plants grown under WW (**j**) and DS (**k**) conditions. Means with letters a and b show significantly different by *t* test (*P* < 0.05)

*ZmcPGM2* expression was inhibited by severe drought stress [45] (Fig. 6a). The SNP chr5.S_10857363 of *ZmcPGM2* was significant associated with maize SR (*P* = 5.6 × 10^−3^, GLM plus 3PCs), and plants with the A allele showed higher survival rates than those with the C allele [13] (Fig. 6b). These results indicated a role of *ZmcPGM2* in regulation of maize drought tolerance. SNP Chr5.S_10856121 had strong LD (*R*^2^ > 0.8) with four other less significant SNPs (chr5.S_10855874, chr5.S_10855875, chr5.S_10857363, and chr5.S_10858751). Re-sequencing to the genomic DNA of *ZmcPGM2* in the maize populations did not detect more significant genomic variations. Further analyses showed that SNPs Chr5.S_10856121, chr5.S_10857363 and chr5.S_10858751 are synonymous variations, while chr5.S_10855874 and chr5.S_10855875 are located in *ZmcPGM2* 3′-untranslated region, and showed significant associations with i-trait ddT200_D40_R and SR (Additional file 10: Figure S9a-e), indicating that SNPs chr5.S_10855874 and chr5.S_10855875 could be the potential causal variants that regulate i-traits and drought tolerance. We next used *Zmcpgm2* mutants to test a possible role of *ZmcPGM2* in maize drought tolerance. Detached leaves from *Zmcpgm2* mutants lost water more slowly than WT leaves under dehydration conditions (Fig. 6c). More *Zmcpgm2* mutants than WT survived after drought stress (Fig. 6d, e), indicating that *Zmcpgm2* mutants were more tolerant to drought and that *ZmcPGM2* had a negative role in maize drought tolerance. Although the photosynthetic rate, stomatal conductance, transpiration rate, and water use efficiency (WUE) showed slightly higher levels in WT plants under WW conditions, these indices were significantly higher in *Zmcpgm2* mutants after severe drought stress (SM < 15%) (Fig. 6f–i). We deduced that the weaker role of *ZmcPGM2* promoted higher WUE and photosynthetic rates under DS conditions, which benefitted maize drought tolerance. The anthesis-silking interval (ASI) is an important maize flowering trait, the shorter the ASI, the better for pollen and silk to meet with each other to produce seeds. We observed that the ASIs of *Zmcpgm2* mutants were significantly shorter than those in WT plants under both WW and DS conditions in the field, indicating that *ZmcPGM2* could also play important roles in flowering regulation.

### *ZmFAB1A* was a key regulator of i-trait dT233_R and maize drought tolerance

The Arabidopsis FAB1A/B regulates the endomembrane homeostasis of pleiotropic developmental processes and is required for pollen development [46, 47], but their roles in crop stress responses remain elusive. There were 11 SNPs in the *ZmFAB1A* locus (GRMZM2G132373) that showed significant association with i-trait dT233_D40_R (the ratio of first-order derivative of the 959 nm total reflectance under drought stress to first-order derivative of the 959 nm total reflectance under well-water condition at 40 days after sowing under DS/WW conditions) (Additional file 10: Figure S10a-c, Additional file 1: Table S7). The most significant SNP chr6.S_117795068 (*P* = 1.51 × 10^−6^, MLM) explained 7.2% of the phenotypic variance and had high linkage with 10 other significant SNPs (*R*^*2*^ = 0.9) (Additional file 10: Figure S10d). Plants with the allele G of the most significant SNP had higher levels of dT233_D40_R than those with the allele C (Additional file 10: Figure S10e). A premature stop mutant *Zmfab1a*, which had a stop mutation at Gln (409) (Additional file 10: Figure S10f), was obtained to further verify the function of *ZmFAB1A*. We grew B73 WT and *ZmFAB1A* mutant plants under WW and DS conditions and investigated the i-traits dT233 and dT233_R at different growth/stress stages (Additional file 10: Figure S10g). The results showed that the levels of dT233_R were higher in *Zmfab1a* than in WT plants after slight or severe drought stress (Additional file 10: Figure S10h-j) and demonstrated that *ZmFAB1A* had a role in regulation of i-trait dT233_R.

The expression of *ZmFAB1A* was increased under severe drought stress (Additional file 10: Figure S10k). Plants with the G allele had higher survival rates after drought stress than those with the C allele (Additional file 10: Figure S10l), which suggested a role of *ZmFAB1A* in maize drought tolerance. Re-sequencing the genomic DNA of *ZmFAB1A* in the maize populations did not detect new significant genomic variations. Analyses to the 11 significant SNPs (tightly linked to each other, *R*^*2*^ = 0.9) showed that 4 were synonymous variations and 7 were missense variations, including chr6.S_117795068 (46^Asp/Glu^), chr6.S_117795706 (231^Asp/Asn^), chr6.S_117795706 (592^Glu/Val^), chr6.S_117795706 (665^Ala/Val^), chr6.S_117795706 (1020^Pro/Arg^), chr6.S_117795706 (1072^Met/Thr^), chr6.S_117795706 (1112^Gln/Pro^), which could be potential causative variations. We further verified the function of *ZmFAB1A* in drought tolerance and the results showed that *Zmfab1a* mutants had higher survival rates than those of WT plants after drought stress (Additional file 10: Figure S10m and n). Moreover, as compared to WT plants, *Zmfab1a* mutants had higher photosynthetic rates, stomatal conductance, and transpiration rates after drought stress with SM < 20% (Additional file 10: Figure S10o-q), and higher WUE after severe drought stress (SM = 12%) (Additional file 10: Figure S10r). Together, these data demonstrated an important role of *ZmFAB1A* in regulation of maize photosynthesis, WUE and drought tolerance.

### Potential utilization of the candidate genes and i-traits

**Fig. 7:**
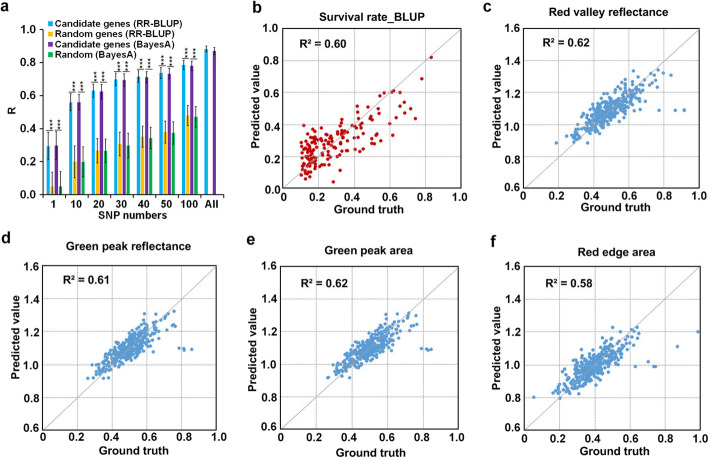
Prediction of maize drought tolerance by candidate genes and i-traits. **a** Drought tolerance selection accuracies by different amounts of candidate and random genes with RR-BLUP and Bayes A models (see “Materials and methods”). The significances were determined by *t* test: ***, *P* < 0.001. **b** Survival rate predicted by combining 15 i-traits across the 4 time points. **c–f** Prediction of four known spectral indexes: red valley reflectance (**c**), green peak reflectance (**d**), green peak area (**e**), and red edge area (**f**), respectively

Genomic selection (GS) is helpful in rapid selection of the superior genetic components that associated with given phenotypes. Because GS utilizes all genetic makers to predict the performance of certain candidates in selection, it is therefore a very useful and effective approach to predict the values of certain genetic makers in breeding [48]. Based on the i-traits collected in this study, we identified more than two thousands of candidate drought-tolerant genes. We performed GS with ridge regression best linear unbiased predictor (RR-BLUP) [49] and Bayes A (Method) to the candidate genes to see the accuracy of their certain combinations in selection of AMP drought-tolerant phenotype survival rates. The randomly selected same amount genes from maize genome (excluded candidate genes) were used in the control analysis. The results showed that the selection accuracies of maize drought tolerance by the candidate genes were significantly higher than those by random genes (Fig. 7a), indicating that these candidate genes could be potential genetic markers in drought-tolerant maize selection and breeding.

To know if the i-traits could be potential biomarkers, we evaluated 1311 ratio i-traits (DS/WW, with significant trait loci associations) in explaining the phenotypic variance of survival rates using a linear stepwise regression model. The results showed that up to 60% of the phenotypic variance in survival rates could be explained by combining 15 i-traits across the 4 time points (Fig. 7b; Additional file 1: Table S20), indicating that these i-traits could be used as markers to select drought-tolerant maize germplasm. Interestingly, 53% of these marker i-traits, including the i-trait ddT200 that was associated with new drought-tolerant gene *ZmcPGM2*, had wavelengths of 780–1000 nm (Fig. 5; Additional file 1: Table S20). These 15 marker i-traits were further compared with four known spectral indexes including red valley reflectance, green peak reflectance, green peak area, and red edge area, which are widely used in agricultural remote sensing to indicate the chlorophyll or water content, and crop health [50, 51]. The results showed good correlation of these marker i-traits with the four indexes (Fig. 7c–f). For example, 58% of the phenotypic variance of red edge area was explained using two markers A248, ddT200 (Fig. 7f), indicating that these markers reflected the change in chlorophyll or water content and could be used to dynamically monitor drought responses and screen for maize accessions with higher drought resistance. The cross-validation of the observation for survival rates and four spectral indexes are shown in Additional file 1: Table S20.

## Discussion

The genetic architecture of drought tolerance is complex and controlled by multiple quantitative trait loci (QTLs) with small effects, and the traditional phenotyping of drought tolerance is still a bottleneck of genetic studies [15, 52]. In this study, we used HSI, CT, and RGB imaging systems to obtain high-dimensional i-traits of a big maize population. Most of these i-traits effectively reflected the dynamic responses of maize to drought. PCA analyses indicated that HSI-derived i-traits were better than RGB or CT-derived i-traits at reflecting the drought responses in the seedling stage (Fig. 2, Additional file 10: Figure S1d-f). It has been reported that metabolites, especially secondary metabolites, are extremely tissue-specific and drought-sensitive [53–55]. The HSI-derived i-traits might largely reflect the maize metabolite levels during drought responses. As proof of concept, the HSI-derived i-trait ddT200_D40_R (the ratio of second-order derivative of the 880 nm total reflectance under drought stress to second-order derivative of the 880 nm total reflectance under well-water at 40 days after sowing) was mainly associated with sugar content (Fig. 5), and a previous study has indicated the associations between metabolites and hyperspectral signatures [56]. Combining metabolite profiling and HSI-derived i-traits analyses would be helpful to further reveal the biochemical and biological roles of these i-traits in plant drought tolerance.

Previously, the RGB-derived i-traits have been used to reveal the genetic basis of drought responses in rice [20]. According to our analyses in maize, very few candidate genes (~ 4.1%) were simultaneously detected with GWAS in RGB-derived, HSI-derived, or CT-derived i-traits (Fig. 3a). In addition, there were distinct pathways where the candidate genes associated with different i-traits were enriched in (Fig. 3a–c, Additional file 10: Figure S4). These data indicated tremendous difference in genetic control of these three types of i-traits. Moreover, the genes that were associated with HSI-derived i-traits represented the majority of the candidate gene sets detected in this study. These observations together suggested the necessity and importance of using different optical imaging methods and pipelines to extract dynamic i-traits in order to get broader and deeper insights into crop drought responses. To our knowledge, this is the first study using HSI and CT-derived i-traits to track plant internal drought responses in a large crop population. Our data thus represent the comprehensive data sets reflecting both internal (HSI, CT i-traits) and external (RGB i-traits) drought responses of big crop populations. In this study, the majority of the candidate genes were identified with ratio i-traits, including some genes with known roles and two novel drought-tolerant genes *ZmcPGM2* and *ZmFAB1A* (Figs. 5 and 6, Additional file 10: Figure S10), indicating the strong power of these i-traits in mapping drought-tolerant genes via GWAS. The possible reason is that these ratio i-traits are enriched with more phenotypic variance, both qualitatively and quantitatively, in response to drought. The data of our study demonstrate that multiple imaging (i-trait)-based GWAS is a powerful and promising approach to dissect the genetic architecture of drought-tolerant traits. This approach could be also suitable for studying the complex traits of other crops. However, it is worthy to note that rare alleles have been reported to play important roles in regulation crop agronomic traits, including grain yield and disease resistance [57, 58]. We used the SNP markers with minor allele frequency no less than 0.05 for GWAS to lower the noise effect; therefore, some rare alleles important for maize drought response might not be detected based on the current maize panel. New populations and statistical approaches need to be developed to study these rare alleles in crop drought responses.

Sugars are very sensitive to drought and ensure the carbohydrate supply from source to sink tissues during the stress [59]. Many sugars respond to drought at very early stage and function as signal molecules, thus have important roles in plant drought tolerance [59–61]. Enhanced expression of some sugar synthetic genes, or application of exogenous sugars promoted plant drought tolerance in terms of growth and yield [62], suggesting that absolute sugar contents of plants play positive roles in drought tolerance. Our study of *ZmcPGM2* demonstrated another scenario of sugar role in plant drought responses, that is, the relatively elevated sugar contents (increased ratio of sugar contents, DS/WW) were also important for the fitness of maize to drought (Figs. 5 and 6). Therefore, this index (relatively elevated sugar contents) could be used as potential and new physiological marker to identify drought-tolerant maize germplasms. This notion was further evidenced by the involvement of i-trait ddT200_D40_R (reflecting ratio change of sugar contents), together with few other i-traits, in prediction of maize drought tolerance (Fig. 7). EMS mutagenesis has been widely used to generate plant materials not only for functional studies but also for plant breeding [8]. Previous studies have suggested a breeding strategy for crop drought tolerance: breeding for higher drought tolerance could simultaneously select shorter plants [15]. The EMS mutant *Zmcpgm2* could be useful in maize breeding due to (1) its relative dwarfism but stronger stems under drought stress (Additional file 10: Figure S8), indicating its lodging-resistance; (2) its higher water use efficiency and survival rates under drought stress, demonstrating its tolerance to drought; (3) its shorter ASI. *Zmfab1a* mutants showed similar phenomena (Additional file 10: Figure S10), which could also be useful genetic materials for maize drought tolerance breeding.

Maize was domesticated from its ancestor teosinte 9000 years ago [63]. The modern maize cultivars have been further improved with many agronomic traits based on the domesticated landraces [64]. There have been thousands of genes that play roles in maize domestication and improvement [64, 65]. In this study, we identified thousands of candidate drought-tolerant genes based on a large-scale of i-traits, which, to our knowledge, are the biggest amount of maize dynamic responses to drought detected so far. It has been reported that the sensitivity of modern maize to drought has steadily and significantly increased during the past decades [66], which could be due to the possible depletion of drought-tolerant genes in the processes of maize domestication and improvement (Additional file 10: Figure S11). With the global climate change, the maize yield loss from drought could be more severe than model predictions [67]. To meet the environmental sustainability and high yield demands, it has been argued that taking back the “lost” genes or alleles (the so-called re-domestication), coupled with precise de novo genetic design, is necessarily required to develop new varieties with both stress tolerance and high yield potential [68]. The genes and their natural variations identified in this study could provide invaluable genetic resources and targets for this purpose.

## Materials and methods

### Plant materials, growth conditions, and experiment design

As shown in Fig. 1a, an association mapping panel (AMP) composed of 368 diverse inbred lines [69, 70], with 2 treatments: DS and WW was planted in the RAP [27] with updated HSI and CT scanners in 2 replicates. Seeds were sown directly in pots with 4.5 kg soil on March 27, 2017, and the WW group was sown 1 day earlier than the DS group. After sowing, all the plants were watered and then covered with film which was removed when seedlings emerged. Fertilizing was carried out at sowing, 3-leaf stage and 10-leaf seedling stage (60 kg of water + 370.68 g of carbamide + 330.76 g of potassium dihydrogen phosphate + 94.24 g of potassium chloride, to be fully dissolved with 150 mL of liquid fertilizer). From the 4-leaf stage (D25), the DS group was stopped irrigation, the WW group was watering normally, and the soil moisture (SM) was measured by a DELTA-T Soil moisture Kit (Delta-T Devices Ltd., UK). As shown in Fig. 1a, the DS conditions are relative to the WW conditions in this study, with the soil moistures of DS conditions dropping from 50 to ~ 10%, while with the soil moistures of WW conditions keeping at ~ 50%. At the seedling stage, all the maize accessions were screened at 10 time points for RGB imaging (once every 3 d starting from D25 to D52), and 4 time points for HSI imaging (once every 6 days starting from D34 to D52).

In order to phenotype the dynamic drought response of the AMP during the flowering stage, two replicates of the WW group in the seedling experiment were divided into one DS group and one WW group, and the drought treatment was the same as for the seedling stage. At the flowering stage, the AMP was screened with 3 time points using CT (once every 7 days starting from D84 to D98). The entire experimental design, inspection dates, weather conditions, and SM are provided in Additional file 1: Table S21.

### Image analysis and trait extraction

After a binary data stream of HSI imaging was acquired for one plant, the binary data stream was reorganized to 250 hyperspectral images. After image segmentation and trait calculation, 2000 hyperspectral i-traits, which included total reflectance related traits, average reflectance related traits, and logarithm related traits, were calculated (Additional file 1: Table S2, Additional file 9: Note S1 and 2). For each accession, 3 different treatments (DS, WW, DS/WW) were inspected at 4 time points, which resulted in 24,000 i-traits.

For each RGB scanning of one maize plant, 20 side-view RGB images from 360 angles were obtained. After image determination with calculation of maximum plant width, image segmentation, and traits, 67 RGB i-traits were extracted (Additional file 1: Table S2, Additional file 9: Note S1 and 2). For each accession, 3 different treatments (DS, WW, DS/WW) were inspected at 10 time points, which resulted in 2010 i-traits.

For each CT scan of one maize plant, one series of 360 X-ray-projected images (step 1°, total angle 1° × 360, ~ 360°) was collected by the high-throughput micro-CT-RGB system (HCR) [71]. The power of the X-ray source was set to 40 KV and 400 μA, and the spatial resolution of the HCR was set as ~ 36 μm. After sinogram extracting, image reconstruction, image segmentation, and calculation of traits, 100 CT traits were obtained (Additional file 1: Table S2, Additional file 9: Note S1 and 2). For each accession, 3 different treatments (DS, WW, DS/WW) were inspected at 3 time points, which resulted in 900 CT i-traits. The HSI, RGB, and CT image analyses were developed using LabVIEW 2015 (National Instruments, USA) and dynamic link library generated using Visual Studio 2013 (Microsoft, USA).

### Selection of drought-responsive i-traits

After a total of 26910 i-traits were obtained, the drought-related i-traits were selected using the following steps: (1) First, a 3σ criterion was used to eliminate abnormal data, which were defined as values greater than the mean value*±* 3σ. The basic concept of 3σ or PauTa criterion is to take the given confidence probability 99.7% as the standard and the triple standard deviation limit as the basis. Any error exceeding this limit is considered not belonging to the category of random error, but to the gross error. The measurement value with gross error is called abnormal value, which is eliminated from the measurement data. (2) After filtering outliers, an independent-samples *t*-test was used to select i-traits with significant differences between the DS group and the WW group, using a 95% confidence interval. (3) Multilayer perceptron (MLP) was used to sort the i-traits depending on their importance for classification of the DS group and the WW group. In order to reduce the error due to random results, the multilayer perceptron operation was repeated five times. If the average value of importance of an i-trait was less than 50%, it would be deleted. (4) Finally, we also checked the heritability (*H*^*2*^) of the i-traits, which was calculated for each i-trait as follows:

$$ {H}^2={\sigma^2}_G/\left[{\sigma^2}_{G+}{\sigma^2}_e/r\right] $$

where ***σ***^***2***^_***G***_ is the genotypic variance, ***σ***^***2***^_***e***_ is the error variance, and r is the number of replications. The i-traits with higher heritability (*H*^*2*^ ≥ 0.2) were retained for further analysis. The outlier filtering was performed using LabVIEW 2015 (National Instruments, USA). The multilayer perceptron and independent-samples t-tests were performed with SPSS software version 25.0 (IBM, USA). The heritability was calculated using the lmer function in the lme4 package in the R environment [26] (http://www.r-project.org/) [72], and heritability screening was implemented with LabVIEW 2015 (National Instruments, USA). The selected RGB, HSI, and CT i-traits are listed in Additional file 1, Table S3-S5.

### Genome-wide association studies

In this study, a genome-wide association study (GWAS) for i-traits was conducted to test the statistical associations between genotype and phenotype (i-traits) using a mixed linear model [25, 73, 74] (MLM, Q + K). SNPs with a minor allele frequency (MAF ≥ 0.05) in the 368 lines were employed in the association analysis. GWAS was performed with TASEEL5.0 software [75] using the uncompressed P3D model. In order to control the type I error rate, the p value of each SNP was calculated and significance was defined at a uniform threshold of p ≤ 1.8 × 10^−6^ (p = 1/n; n = 558,650, total markers used) [25]. For each significant i-trait locus, the significantly associated SNP and its corresponding candidate gene are reported in Additional file 1, Table S7. Only genes that had significantly associated SNPs within range of the gene were selected as candidate genes. If other significant SNPs were identified within 100 kb upstream or downstream of a significant SNP, these adjacent SNPs were merged. This merging operation was repeated until no more SNPs could be merged. The merged area was then designated a target QTL.

### eQTL mapping

In order to determine whether the candidate genes were regulated at the transcriptional level, GWAS was used to analyze the relationship between the whole genome SNPs and the expression levels of i-trait-associated candidate genes. In this study, we used the expression data from 197 diverse inbred lines (from a previous published 540 inbred lines for association mapping) under DS and WW conditions, combined with 1.25 million SNPs [41] for GWAS. There are 135 inbred lines of these 197 lines also involved in the 368 inbred lines used in this study. The SNPs used in this analysis have minor allele frequencies (MAF) ≥ 0.05. The p value of each SNP was calculated and significance was defined at a uniform threshold of p ≤ 8.4 × 10^−7^ (p = 1/n; n = total markers used). Then, we extended the 10-kb interval between significant SNPs upstream and downstream as an eQTL interval. If the candidate gene was within this interval, it was considered to be *cis*-regulated; otherwise, it was *trans*-regulated.

### Gene Ontology analysis

GO enrichment analysis was conducted using agriGO [76] (http://bioinfo.cau.edu.cn/agriGO/index.php [77]. A false discovery rate (FDR ≤ 0.05) was used to identify significant GO terms.

### KEGG analysis

The KEGG pathway enrichment analysis was completed in two steps. First, the maize candidate gene IDs were converted and filtered into Entrez Gene IDs by customized scripts using gene information (Zea_mays.gene info) from GenBank. Then, the Entrez IDs were called by the Gene-list Enrichment tool in KOBAS3.0 (http://kobas.cbi.pku.edu.cn/kobas3) [78] to do KEGG pathway enrichment with the default parameters [79]. The cut-off for significance was P < 0.05. Gene-trait network (Fig. 3a) was implemented using Gephi [80] (version 0.9.2).

### Genome selection analysis

The genome selection analysis was divided into three steps: First, the whole maize genome genes were divided into two gene pools: the candidate gene pool and the random gene pool (excluded candidate genes), and the SNP with the most significant association with survival rate was selected to represent this gene. Second, different number of the most significant SNPs (top1, top10, top20, top30, top40, top50, top100, all genome) were selected for candidate genes. For random genes, SNPs corresponding to the number of candidate genes were randomly selected each time, and the process was repeated 50 times. Finally, after obtaining all SNPs of the selected genes (MAF ≥ 0.05), the R package RR-BLUP (http://www.r-project.org/) [72] and BGLR (Bayes A) [81] was used to predict the survival rates of AMP, 50% of which were used as training and 50% as testing. In this process, candidate genes were repeated 500 times and random genes were repeated 10 times. The final results in comparison were based on 500 repeats of GS analyses for each given number of gene sets. The correlation coefficient r between the predicted value and the observed value is used to evaluate the accuracy of the prediction.

### Metabolomic study using GC-MS

Leaves of WW and DS B73 wild type and *Zmcpgm2* mutant plants were used for the metabolomic experiments with three biological replicates of each. Leaf samples frozen in liquid nitrogen were ground in a Mixer/mill (MM400; Retsch) with a steel ball for 30 s at 30 HZ. Fifty milligrams of each sample was extracted with 3:1 methyl tert-butyl: ether: methanol, v:v, in which 10 μL of 1 mg/mL 13C ribitol was added as an internal standard [82]. In total, 200 μL of the polar phase for each sample was dried in a SpeedVac concentrator without heating. The sample was re-dissolved in 50 μL 20 mg/mL O-methylhydroxylamin hydrochloride (Sigma, Steinheim, Switzerland) in pyridine, vortexed, and incubated at 37 °C for 120 min. Then, 70 μL N-methylN-trimethylsily trifluoroacetamide (Sigma, Steinheim, Switzerland) was added to the mixture, vortexed, and incubated at 37 °C on a shaker for 30 min. The silyl-derivatized samples were analyzed by GC-MS (7890A-5975C, Agilent, USA).

One microliter was taken from each sample and injected into the GC-MS at 270 °C in a split mode (50: 1) with helium carrier gas (> 99.999% purity) flow set to 1 mL/min and separated by a DB-35MS UI (30 m × 0.25 mm, 0.25 μm) capillary column. The temperature was isothermal for 4 min at 90 °C, followed by an 8 °C per min ramp up to 205 °C, then held for 2 min, and finally ramped up at a rate of 15 °C per min to 310 °C, held for 2 min. The transfer line temperature was set to 300 °C, and the ion source temperature was set to 230 °C. The mass range analyzed was from m/z 85 to 700. Agilent MassHunter Qualitative Analysis (version B06.00) software and Agilent MassHunter Quantitative Analysis (version B.07.01) were jointly used for GC-MS data analyses. NIST library and in-house database established using authentic standards were used together for metabolite identification.

### Verify the drought resistance of *Zmcpgm2* and *Zmfab1a*

In order to verify the candidate genes’ functions in drought resistance, we ordered EMS mutants of *Zmcpgm2* and *Zmfab1a* and identified their genotypes by the KASP method (LGC, UK). The putative target EMS sites of the genes were then sequenced to confirm the mutation (Additional file 1, Table S22). Homozygous mutants were purified by backcrossing and were then amplified in Hainan (18° 25′ N, 109° 51′ E).

From late March to early July 2019, we planted *Zmcpgm2*, *Zmfab1a* and B73 (wild type) for i-trait (RGB, HSI, CT) detection. Each genotype was divided into two treatments: DS and WW, with at least 10 pots per treatment. The maize plants were transferred into the RAP at the 2-leaf stage. Drought treatments and planting methods were as described above. All the maize seedlings were subjected to RGB and HSI imaging at 4 time points (the average SM under drought stress was 41%, 19%, 15%, and 10% at the successive time points). At the flowering stage, all maize plants were screened by CT imaging at 4 time points with at least 5 pots per treatment (the average SM under drought stress was 45%, 20%, 15%, and 10%, respectively). The inspection dates, weather conditions, and SM data are provided in Additional file 1: Table S21.

For the survival rate experiment, we planted *Zmcpgm2*, *Zmfab1a*, and B73 in autumn 2019. Each genotype was divided into two treatments: DS and WW, with at least 10 pots per treatment in 3 replicates. Irrigation was stopped at the 4-leaf stage, and the drought treatment was the same as described previously. When the SM reached 10%, the plants were re-watered, and the survival rate was determined 3 days after re-watering.

The photosynthesis experiment was conducted in autumn of 2019. After *Zmcpgm2*, *Zmfab1a*, and B73 seeds germinated on a petri dish, they were transplanted into plastic pots (length × width × height = 42 cm × 32 cm × 15 cm). Each pot was filled with 12.5 kg soil. Fertilizing was performed before transplanting and the 3-leaf stage. The mutants and B73 were grown in the pots side-by-side, with a total of 18 plants for each genotype, and there were 3 replicates of each experiment. Before the maize 4-leaf stage, the pots were planted outdoors. The temperature of the growth chamber was 28 °C and the light cycle was 12 h light/12 h dark. Drought stress was initiated by ceasing irrigation at the 4-leaf stage. During the drought stress, we used a LI-COR6800 (LI-COR, USA) to measure the photosynthesis parameters. Every other day, 6 leaves of the mutant and B73 in each pot were measured starting at 8:30 a.m. After the daily measurement, we used a DELTA-T Soil moisture Kit (Delta-T Devices Ltd., UK) to measure SM.

For water loss measurements, leaves were detached from *Zmcpgm2*, *Zmfab1a*, and B73 at the 6-leaf stage and were exposed to air at room temperature. These leaves were weighed at various time intervals, and the loss of fresh weight (percentage) was used to calculate water loss rate.

### Data availability

The selected RGB, HSI, and CT i-traits are listed in Additional file 1: Table S3-S5. In total, 4322 significant SNPs and 2318 candidate genes associated with i-traits are listed in Additional file 1: Table S7. All the images, phenotypic data, and genotype data are publicly available for reuse with the link: 10.6084/m9.figshare.14429003.v1 [83]. The code of CT, HSI, and RGB image analysis pipelines could be downloaded via the link: https://github.com/fenghuifh2006/Maize-RGB-CT-HSI-program and 10.5281/zenodo.4690730 [84, 85]. All the figures and supplemental files could be downloaded via the link: 10.6084/m9.figshare.14412572.v1 [86]. All other reasonable requests for data and research materials are available by contacting the corresponding authors.

## Supplementary Information

**Additional file 1:** A master file for Table S1-22.

**Additional file 2: Video S1.** High-throughput phenotyping to extract i-traits.

**Additional file 3: Video S2.** High-throughput hyperspectral images acquisition and analysis.

**Additional file 4: Video S3.** High-throughput RGB images acquisition and analysis.

**Additional file 5: Video S4.** High-throughput CT images acquisition and analysis.

**Additional file 9: Note S1-S2.** Information about the i-traits and the operation guide for the HSI, CT, and RGB data processing programs.

**Additional file 10: Figures S1-S11**.

**Additional file 11.** Review history.
