# Supplementary material for: Using high-throughput multiple optical phenotyping to decipher the genetic architecture of maize drought tolerance
Source: Genome Biol. 2021 Jun 24;22:185. doi: 10.1186/s13059-021-02377-0 (PMC8223302; doi:10.1186/s13059-021-02377-0)
Supplement: Supplementary file 9 — Additional file 9: Note S1-S2. Information about the i-traits and the operation guide for the HSI, CT, and RGB data processing programs. [file 13059_2021_2377_MOESM9_ESM.docx]

# Note S1. Definition of the phenotypic traits (page 1-8)

**HSI**

- **T1~T250**: Total reflectance of the plant under different wavelength

$T_{n}=\sum R_{n}(i,j)$ (1)

where R_n_(i,j) was the reflectance of coordinate (i,j) under n wavelength, (i,j)∈plant ROI, n=1, 2, ……, 250.

- **dT1~dT250**: The first-order derivative of the total reflectance of the plant under different wavelength

$dT_{n}=\frac{1}{2dt}(T_{n+1}-T_{n-1})$ (2)

n=2, 3, ……, 249.

- **ddT1~ddT250**: The second-order derivative of the total reflectance of the plant under different wavelength

$ddT_{n}=\frac{1}{2dt}(dT_{n+1}-dT_{n-1})$ (3)

n=2, 3, ……, 249.

- **A1~A250**: Average reflectance of the plant under different wavelength

$A_{n}=\frac{\sum R_{n}(i,j)}{Area}$ (4)

where R_n_(i,j) was the reflectance of coordinate (i,j) under n wavelength, Area was the area of plant ROI, (i,j)∈plant ROI, n=1, 2, ……, 250.

- **dA1~dA250**: The first-order derivative of the average reflectance of the plant under different wavelength

$dA_{n}=\frac{1}{2dt}(A_{n+1}-A_{n-1})$ (5)

n=2, 3, ……, 249.

- **ddA1~ddA250**: The second-order derivative of the average reflectance of the plant under different wavelength

$ddA_{n}=\frac{1}{2dt}(dA_{n+1}-dA_{n-1})$ (6)

n=2, 3, ……, 249.

- **lgT1~lgT250**: The logarithm of the total reflectance of the plant under different wavelength

$\lg T_{n}=\log_{10}(T_{n})$ (7)

n=1, 2, ……, 250

- **lgA1~lgA250**: The logarithm of the average reflectance of the plant under different wavelength

$\lg A_{n}=\log_{10}(A_{n})$ (8)

n=1, 2, ……, 250

**RGB**

- **TPA**: total projected area. Number of foreground pixels attributed by rice plant.
- **GPAR**: green projected area ratio: GPA/TPA. GPA was the number of foreground pixels attributed by green part of rice plant
- **FDNIC**: fractal dimension without image cropping FDNIC: Superimpose boxes with box size of $\delta_{k}$ on the interested object, and calculate the number of boxes that are needed to cover the object, denoted as$N_{\delta_{k}}$. Repeat this process with reducing $\delta_{k}$ until $\delta_{k}$ approaches pixel size. Fractal dimension was calculated using the following Equation.

$FD=\lim_{\delta_{k}\to0} \frac{\ln N_{\delta_{k}}}{-\ln\delta_{k}}$ (9)

- **FDIC**: fractal dimension after image cropping. Cropping the original image to the smaller size with bounding rectangle of rice plant, and calculating the FD with the above steps.
- **MPH**: Height of the bounding rectangle MPH of the object.
- **PW**: Width of the bounding rectangle of the object.
- **HWR**: Height / width ratio: PH/MPW.
- **PP**: Plant perimeter in side view
- **TBR**: Total projected area / bounding rectangle area ratio. A/(PH×MPW)
- **PAR**: Perimeter / projected area ratio. Calculating the outline length of the rice plant, then dividing the length by projected area.
- **PC1-PC6**: Plant compactness. Divide the image into several sub-images using a (5 × 5) window. And calculate the ratio of the foreground pixels to the total number of pixels in each sub-image (5 × 5), denoted as plant compactness in each sub-image (PCs). Categorize PCs into six classes: C1: <10%, C2: 10-20%, C3: 20-40%, C4: 40-60%, C5: 60-80%, C6: 80-100%. Then Count the number of PCs belonging to each class, denoted as NDi (i=1,2…6). At last, leaf compactness of class i (PCi) was computed as the percentage of NDi compared to the sum of NDi.
- **GCV**, green color value in top view. GCV was calculated using the following Equation

$GCV=\frac{\sum R_{i}}{TPA}$ (10)

Where R_i_ was the gray value of foreground pixels of green channel image

- The six histogram traits, including the mean value (**M_HEX**), the standard error (**SE_HEX**), the third moment (**MU3_HEX**), the uniformity (**U_HEX**), the smoothness(**S_HEX**), and the entropy (**E_HEX**), were calculated using the following equations:

$M\_HEX=\sum_{i=0}^{L-1} G_{i}\times p(G_{i})$ (11)

$SE\_HEX=\sqrt{\sum_{i=0}^{L-1} (G_{i}-M\_HEX)^{2}p(G_{i})}$ (12)

$MU3\_HEX=\sum_{i=0}^{L-1} (G_{i}-M\_HEX)^{3}p(G_{i})$ (13)

$U\_HEX\text{=}\sum_{i=0}^{L-1} p^{2}(G_{i})$ (14)

$S\_HEX=1-\frac{1}{1+SE\_HEX^{2}}$ (15)

$E\_HEX=\sum_{i=0}^{L-1} p(G_{i})\times\log_{2}p(G_{i})$ (16)

Where G_i_ was the i-th gray level, and p(G_i_) was the probability of G_i_. L was the maximum gray level. The _I/H/G/S indicated the I/H/G/S images.

- **T1-T15**: The 15 gray level co-occurrence matrix texture traits, including the correlation (T1), the advantages of the small gradient (T2), the advantages of the large gradient (T3), the energy (T4), the intensity inhomogeneity (T5), the gradient inhomogeneity (T6), the mean gray (T7), the mean gradient (T8), the gray entropy (T9), the gradient entropy (T10), the entropy of mixing (T11), the differential moment (T12), the deficit score (T13), the gray variance (T14), and the gradient variance (T15), were calculated using the following equations:

$T1=\frac{1}{\sigma_{1}\sigma_{2}}\sum_{i=1}^{32} \sum_{j=1}^{32} (i-\mu_{1})(j-\mu_{2})P(i,j)$ (17)

$T2=\sum_{i=1}^{32} \sum_{j=1}^{32} \frac{P(i,j)}{j^{2}}/\sum_{i=1}^{32} \sum_{j=1}^{32} P(i,j)$ (18)

$T3=\sum_{i=1}^{32} \sum_{j=1}^{32} j^{2}P(i,j)/\sum_{i=1}^{32} \sum_{j=1}^{32} P(i,j)$ (19)

$T4=\sum_{i=1}^{32} \sum_{j=1}^{32} P(i,j)^{2}$ (20)

$T5=\sum_{i=1}^{32} [\sum_{j=1}^{32} P(i,j)]^{2}/\sum_{i=1}^{32} \sum_{j=1}^{32} P(i,j)$ (21)

$T6=\sum_{j=1}^{32} [\sum_{i=1}^{32} P(i,j)]^{2}/\sum_{i=1}^{32} \sum_{j=1}^{32} P(i,j)$ (22)

$T7=\mu_{1}$ (23)

$T8=\mu_{2}$ (24)

$T9=-\sum_{i=1}^{32} \sum_{j=1}^{32} P(i,j)\cdot\log\sum_{j=1}^{32} P(i,j)$ (25)

$T10=-\sum_{j=1}^{32} \sum_{i=1}^{32} P(i,j)\cdot\log\sum_{i=1}^{32} P(i,j)$ (26)

$T11=-\sum_{i=1}^{32} \sum_{j=1}^{32} P(i,j)\cdot\log P(i,j)$ (27)

$T12=\sum_{i=1}^{32} \sum_{j=1}^{32} (i-j)^{2}P(i,j)$ (28)

$T13=\sum_{i=1}^{32} \sum_{j=1}^{32} \frac{1}{1+(i-j)^{2}}P(i,j)$ (29)

$T14=\sigma_{1}$ (30)

$T15=\sigma_{2}$ (31)

$\mu_{1}=\sum_{i=1}^{32} i\cdot\sum_{j=1}^{32} P(i,j)$ (32)

$\mu_{2}=\sum_{j=1}^{32} j\cdot\sum_{i=1}^{32} P(i,j)$ (33)

$\sigma_{1}=\sqrt{\sum_{i=1}^{32} (i-\mu_{1})^{2}\sum_{j=1}^{32} P(i,j)}$ (34)

$\sigma_{2}=\sqrt{\sum_{j=1}^{32} (j-\mu_{2})^{2}\sum_{i=1}^{32} P(i,j)}$ (35)

- **Area_convex_hull**: The area of convex hull of the plant ROI
- **Perimeter_convex_hull**: The perimeter of convex hull of the plant ROI
- **Elongation_convex_hull**: The max feret diameter divided by equivalent rect short side of convex hull of the plant ROI

$Elongation\_convex\_hull=\frac{F\_convex\_hull}{RF_{b}\_convex\_hull}$ (36)

where F_convex_hull was the distance between the max feret diameter start and the max feret diameter end of the convex hull of the plant ROI. RFb_convex_hull was shortest side of the rectangle with the same area as the particle, and longest side equal in length to the max feret diameter

- **Compactness_convex_hull**: The area divided by the product of bounding rect width and bounding rect height of convex hull of the plant ROI

$Compactness\_convex\_hull=\frac{Area\_convex\_hull}{W\_convex\_hull\times H\_convex\_hull}$ (37)

where W_convex_hull was the bounding rect width of the convex hull of the plant ROI. H_convex_hull was the bounding rect height of the convex hull of the plant ROI

- **Heywood_convex_hull**: The perimeter divided by the circumference of a circle with the same area of convex hull of the plant ROI

$Heywood\_convex\_hull=\frac{Perimeter\_convex\_hull}{2\sqrt{\pi Area\_convex\_hull}}$ (38)

- **Type_convex_hull**: The factor relating area to moment of inertia of convex hull of the plant ROI

 (39)

where I_xx__convex_hull was the moment of Inertia xx of convex hull of the plant ROI. I_yy__convex_hull was the moment of Inertia xy of convex hull of the plant ROI

- **Elongation**: The max feret diameter divided by equivalent rect short side of plant ROI

$Elongation=F/RF_{b}$ (40)

where F was the distance between the max feret diameter start and the max feret diameter end of the plant ROI. RFb was shortest side of the rectangle with the same area as the particle, and longest side equal in length to the max feret diameter

- **Compactness**: The area divided by the product of bounding rect width and bounding rect height of plant ROI

$Compactness=TPA/(W\times H)$ (41)

where W was the bounding rect width of the plant ROI. H was the bounding rect height of the plant ROI

- **Heywood**: The perimeter divided by the circumference of a circle with the same area of plant ROI

$Heywood=\frac{PP}{2\sqrt{\pi TPA}}$ (42)

- **Type**: The factor relating area to moment of inertia of plant ROI

 (43)

where I_xx_ was the moment of Inertia xx of the plant ROI. I_yy_ was the moment of Inertia xy of the plant ROI

- **Area_convex_hull_area**: Plant ROI area/ convex hull area

$Area\_convex\_hull\_area=\frac{TPA}{Area\_convex\_hull}$ (44)

**CT**：

- **Major_axis**: the major axis length of the stem
- **Minor_axis**: the minor axis length of the stem
- **Culm_diameter**: the diameter of the stem
- **Wall_thickness**: the thickness of the stem
- **Culm_wall_area**: the wall area of the stem
- **Cavity_area**: the cavity area
- **Culm_area**: the total area
- **Vascular_area**: the vascular area
- **Hollow_area**: the cavity_area-the vascular_area
- **Duty_cycle**: the vascular area / the cavity_area

The relationship between these parameters is shown in the figure below:


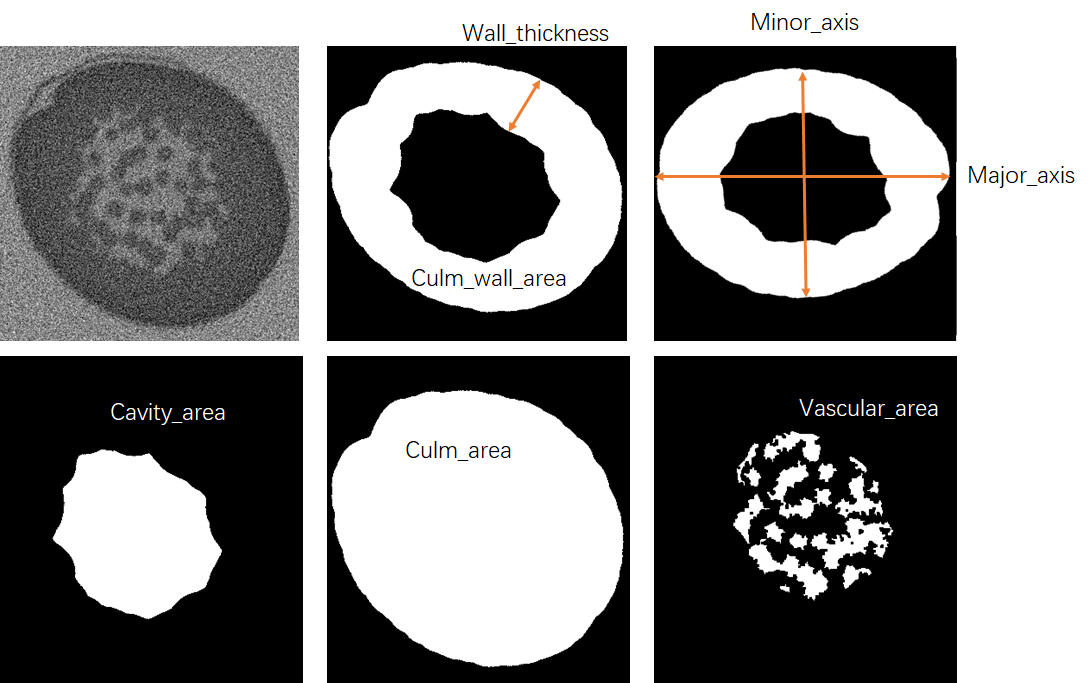


# Note S2. Operation guide for the HSI, CT, and RGB data processing programs (page 9-31)

**Operation guide for the hyperspectral data processing program**

More intuitive information can be obtained in the Supplemental Video 2

Program running environment: Windows 64 bit, LabVIEW2015

1. **Image segmentation**
2. Open the program “1-Main Image segmentation.vi”.


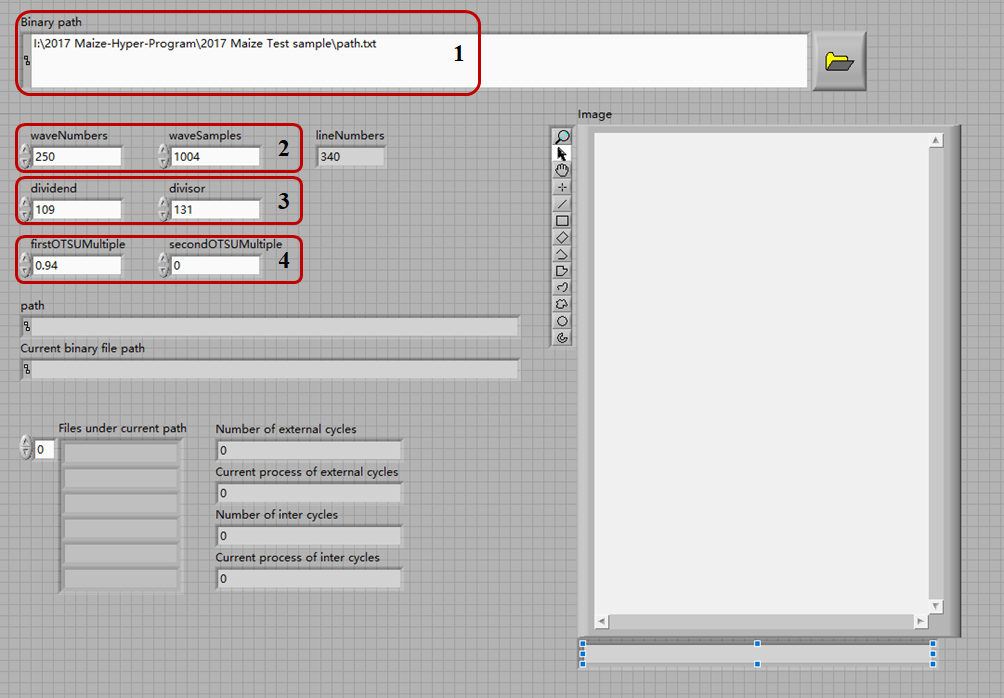


Figure 1 The interface of the program “1-Main Image segmentation.vi”

1. Set parameters. The red box 1 in Figure 1 represents the path of the binary data stream. The red box 2 in Figure 1 represents the parameters of the hyperspectral camera. The red box 3 in Figure 1 represents the image numbers that were used for segmentation. The red box 4 in Figure 1 represents the OTSU related parameters.


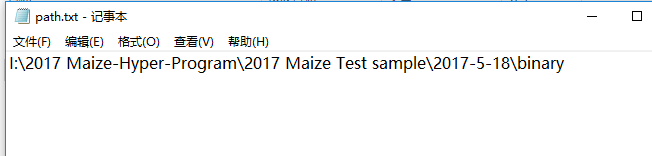


Figure 2 The path of the binary data stream in Figure 1


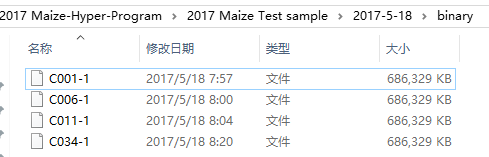


Figure 3 The contents of the folder in Figure 2

1. Press “Ctrl+R” to run the program
2. When the program finished, the results of the image segmentation were saved in a separate folder called “binary segmentation image”


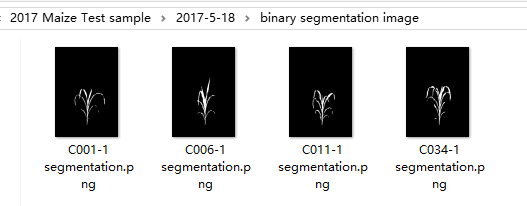


Figure 4 Sample segmentation results images

1. **Total reflectance extraction**
2. Open the program “2-Main Reference calculation.vi”.


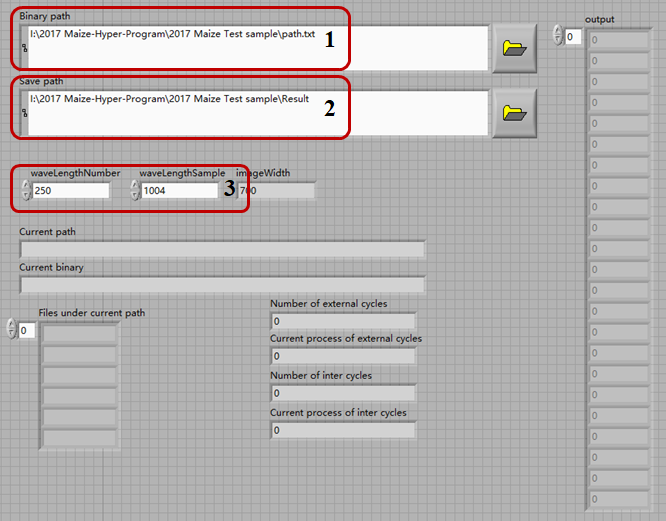


Figure 5 The interface of the program “2-Main Reference calculation.vi”

1. Set parameters. The red box 1 in Figure 5 represents the path of the binary data stream, just like the red box 1 in Figure 1. The red box 2 in Figure 5 represents the results folder path. The red box 3 in Figure 5 represents the parameters of the hyperspectral camera, just like the red box 2 in Figure 1.
2. Press “Ctrl+R” to run the program.
3. When the program finished the results of the reflectance extraction were saved in the folder shown in the red box 2 in Figure 5.


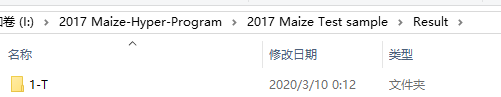


Figure 6 Sample results folder


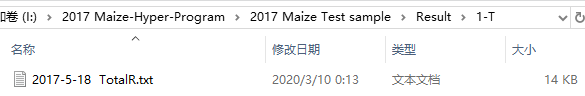


Figure 7 Sample results folder contents


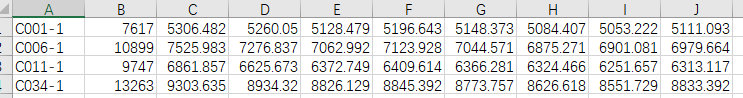


Figure 8 Sample reflectance results

1. **Derivative index calculation and data fusion**
2. Open the program “3-Main Combine data.vi”.


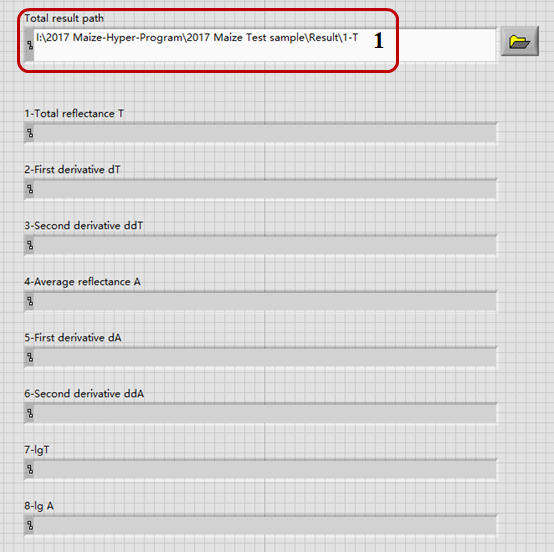


Figure 9 The interface of the program “3-Main Combine data.vi”

1. Set parameters. The red box 1 in Figure 9 represents the path of the total reflectance, just like the red box 2 in Figure 5.
2. Press “Ctrl+R” to run the program.
3. After the program finished, all results were saved in the “Result” folder. The fusion data were saved in the file shown in the red box 1 in Figure 10. The other data were stored in the process results.


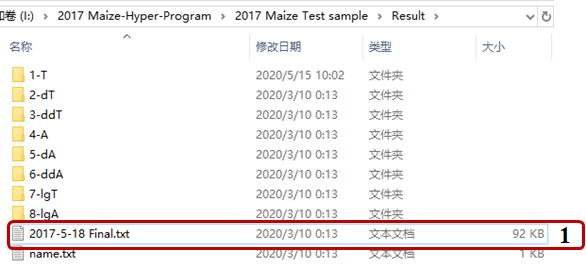


Figure 10 The final results of the program “3-Main Combine data.vi”

**Operation guide for the CT data processing program**

More intuitive information can be obtained in the Supplemental Video 3

Program running environment: Windows 64 bit, LabVIEW2015 32bit

1. **Open the “CT Reconstruction.vi” program and set the path of reading X-ray projected images**

The software reads the x-ray projected images circularly according to the picture names of **Barcode index.txt**, so the first step is to build the text file. Generally, **Barcode index.txt** is relatively simple, which can be edited manually, as shown in Figure 12. The content of **Barcode index.txt** is the barcode index of the maize sample. For example, C363-1 means the maize accession C363.


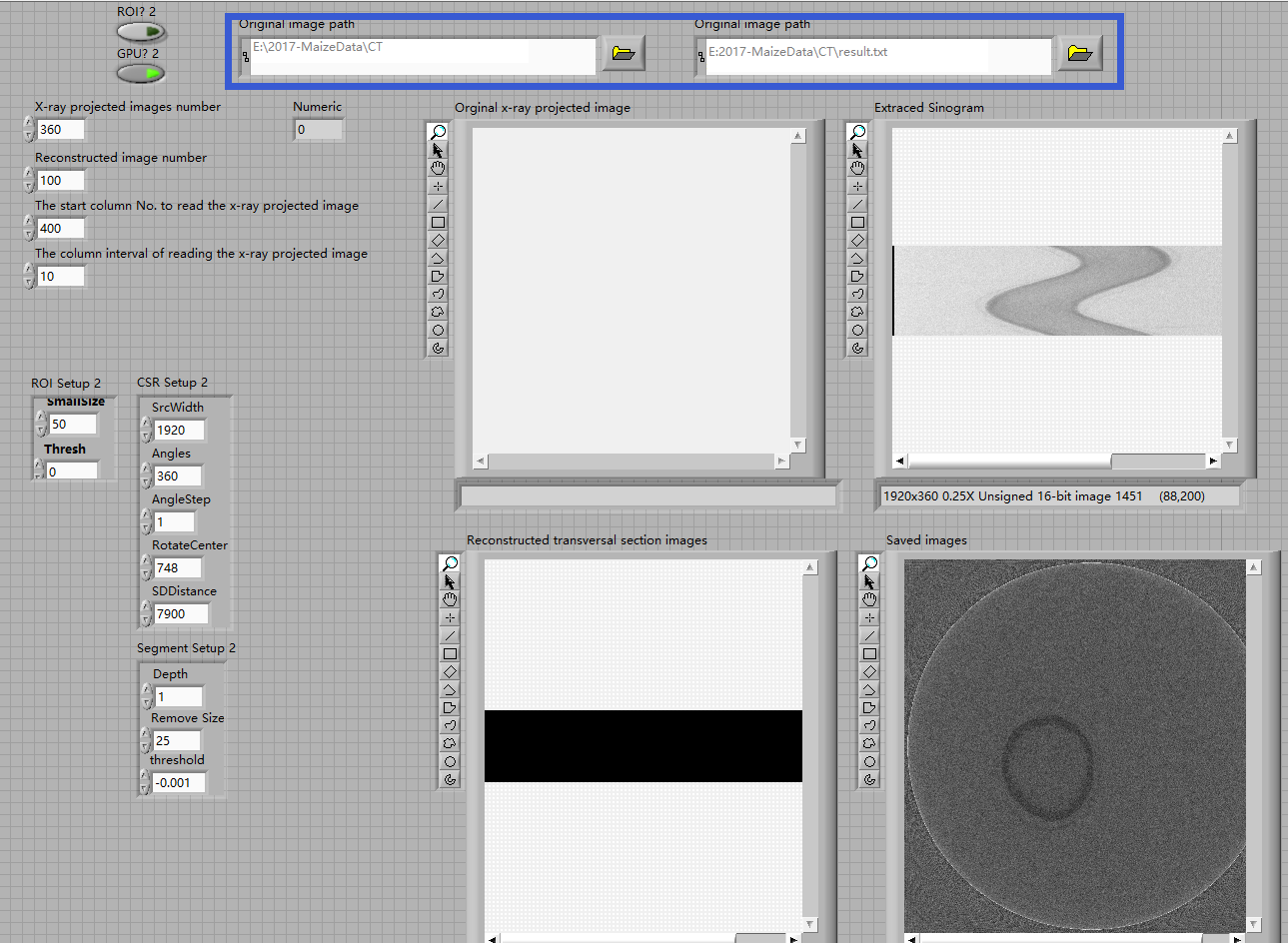


Figure 11 Set the path of reading x-ray projected images


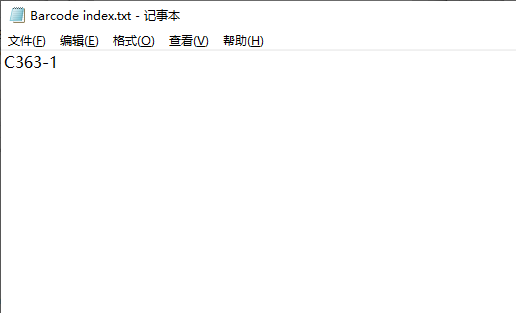


Figure 12 Sample path file content (You can select any folder to be processed)


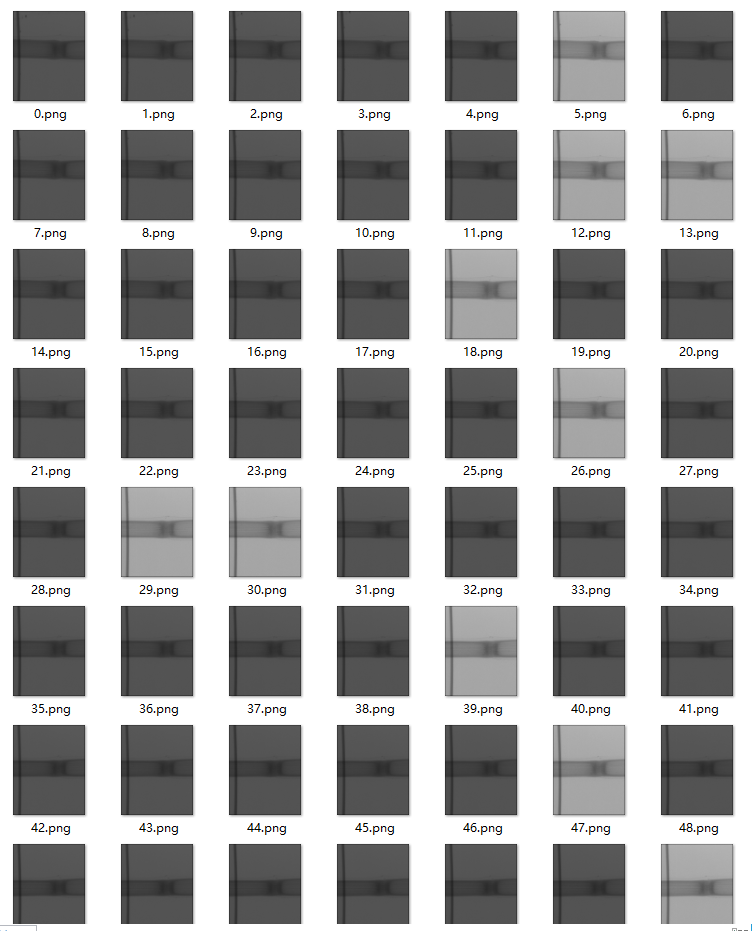


Figure 13 Example of 360 x-ray projected images for one maize plant

1. **Set the parameters of reading X-ray projected images**

Before running the program, we should set the parameters:

1. X-ray projected images number. In the CT experiment for maize plant inspection, X-ray projected images number is set as 360.
2. Reconstructed image number. In the CT experiment for maize plant inspection, the reconstructed image number is set as 10, which means a total of 10 transversal section images at different heights (400, 500, 600, 700, 800, 900, 1000, 1100, 1200, and 1300) are reconstructed for each maize plant. If more images at different height are reconstructed, more time cost should be taken into consideration (~1 second per image reconstruction).
3. The start height No. to read the x-ray projected image. In the CT experiment for maize plant inspection, the start height No. is set as 400.
4. The height interval of reading the x-ray projected image. In the CT experiment for maize plant inspection, the height interval of reading the x-ray projected image is set as 100. This parameter and the start height No. co-determine the transversal section images at different heights (400, 500, 600, 700, 800, 900, 1000, 1100, 1200, and 1300) that were reconstructed.


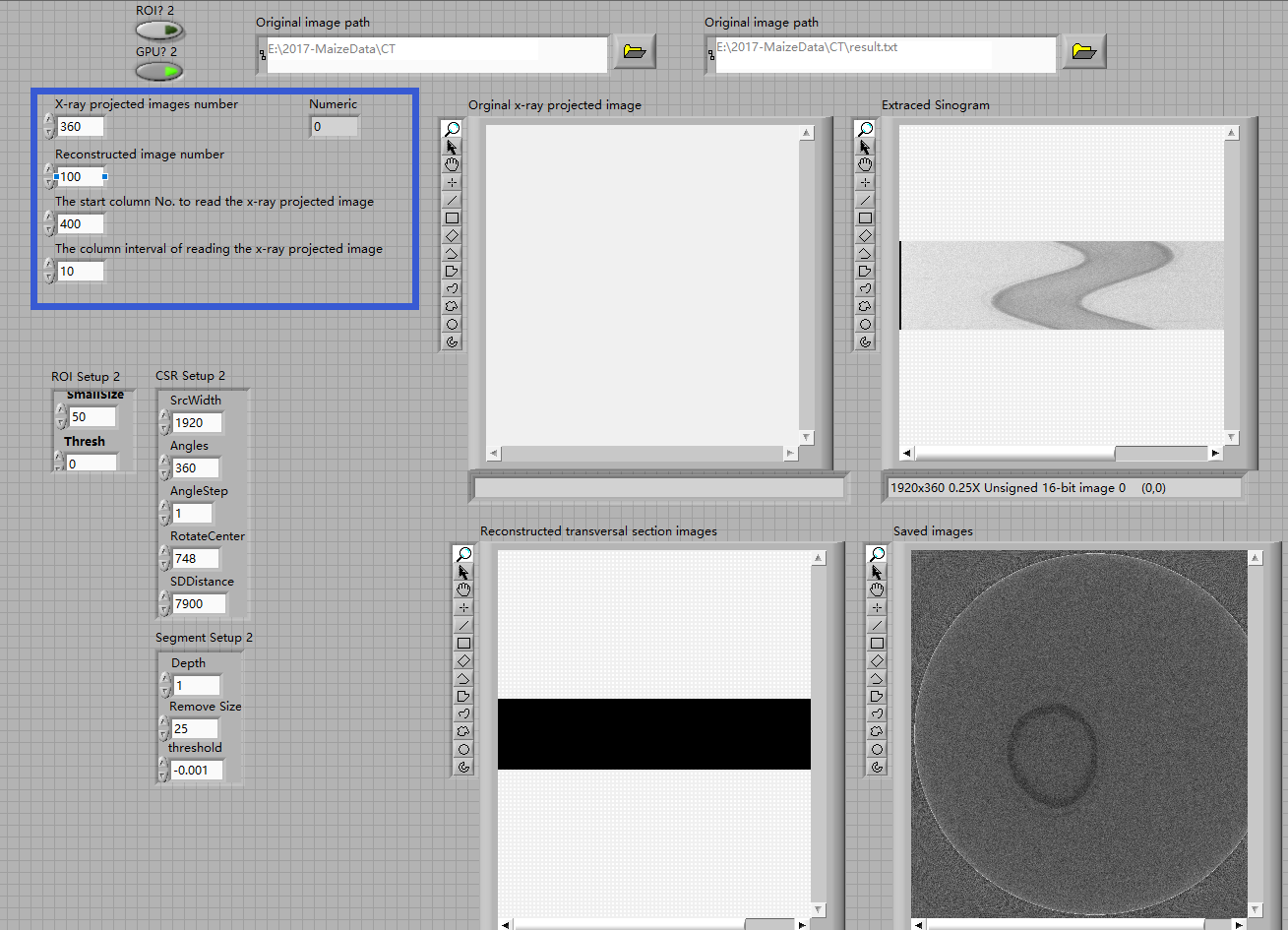


Figure 14 Setting the parameters of reading X-ray projected images

1. **Setting the parameters of X-ray CT reconstruction**

The key parameters of X-ray CT reconstruction are:

1. The width of x-ray panel detector: in this experiment, set as 1920;
2. The total angles: also means the projected image number, in this experiment, set as 360;
3. Angle step: in this experiment, set as 1, which means the angle interval is 1°;
4. Rotate center: in this experiment, set as 748;
5. SDDistance: which means the distance between the x-ray source to detector, in this experiment, set as 7900.


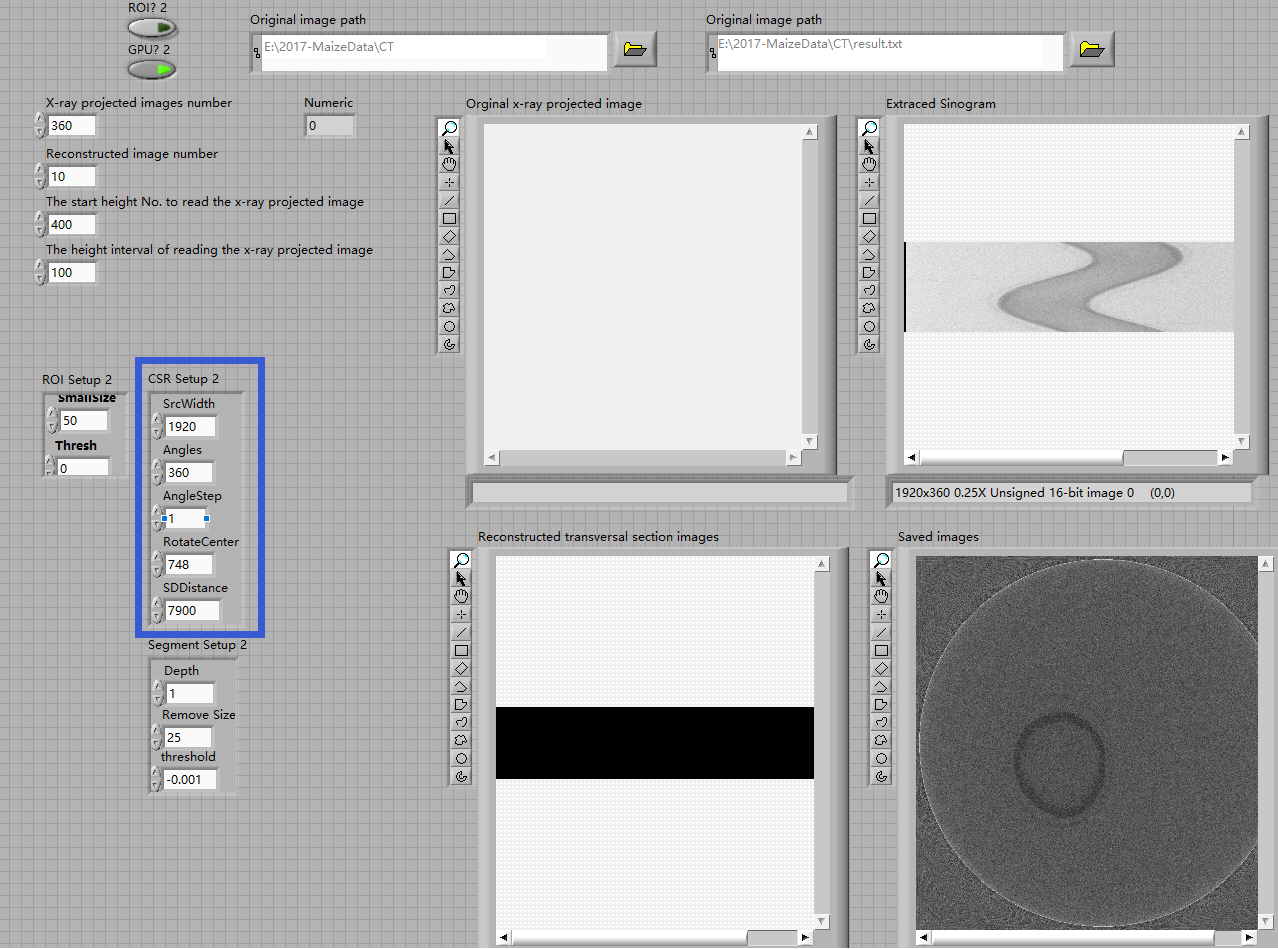


Figure 15 Setting the parameters of X-ray CT reconstruction

1. **Running the X-ray CT reconstruction program and checking the results**

Click “RUN” and wait until all the images are reconstructed. Then check the extracted Sinograms at different heights and reconstructed transversal section images of maize stems at different heights.


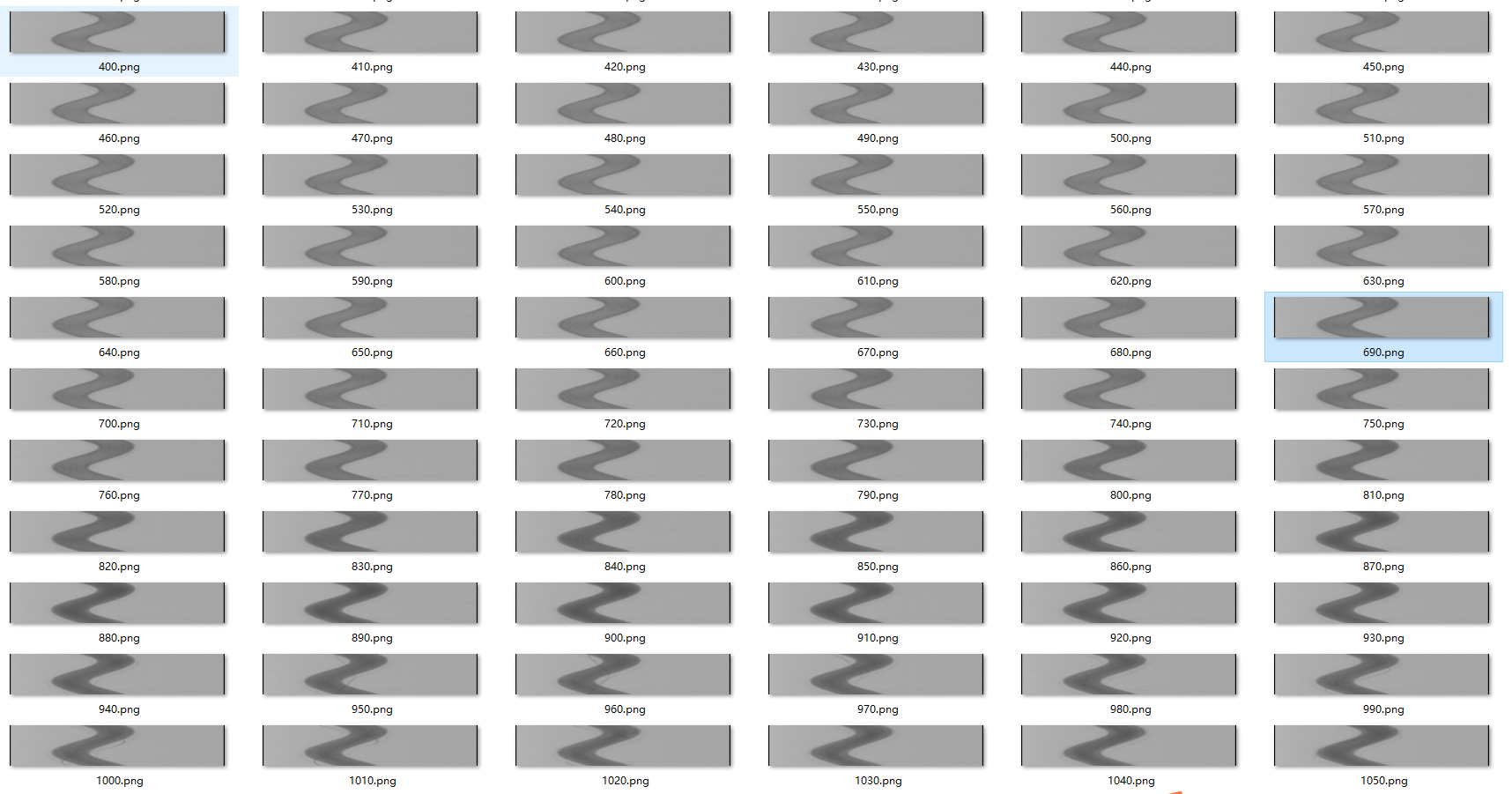


Figure 16 Extracted Sinograms at different heights


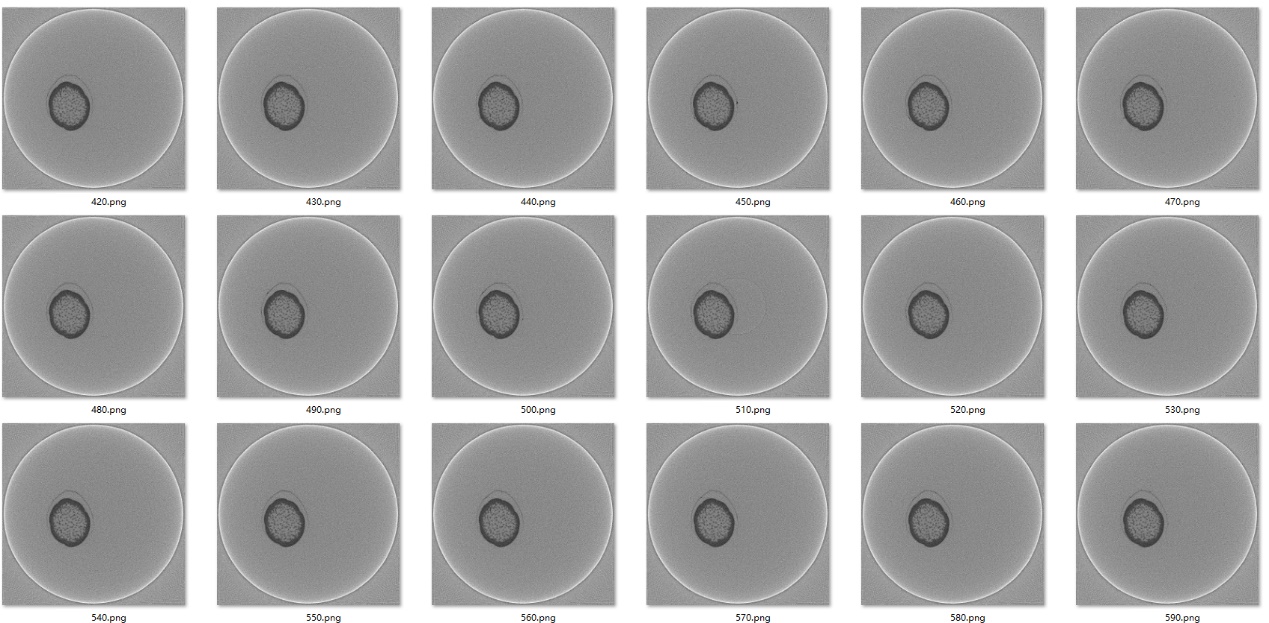


Figure 17 Reconstructed transverse section images of maize stems at different heights

1. **Open the program of “CT traits extraction_Maize stem_English version.vi” and Set the path of reading X-ray projected images**


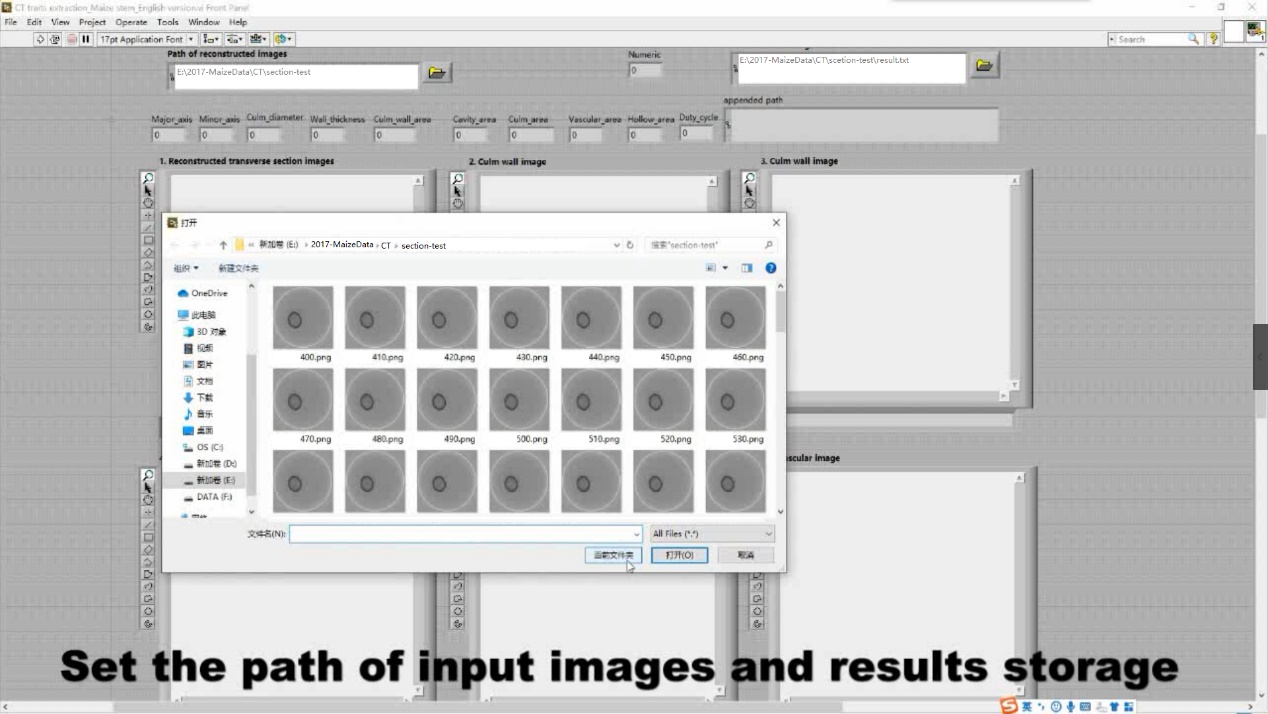


Figure 18 Setting the path of reading X-ray projected images

**Setting the path of input images and results storage**


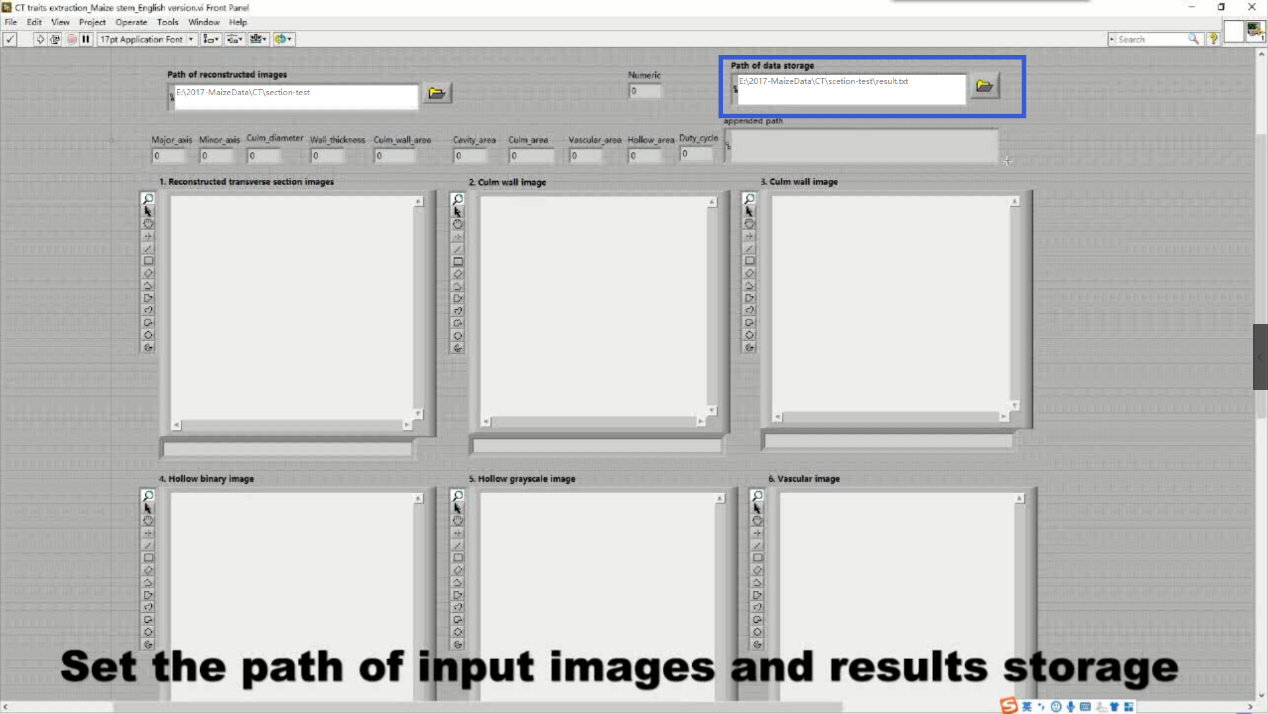


Figure 19 Setting the path of input images and results storage

1. **Run the program and check the stored results**


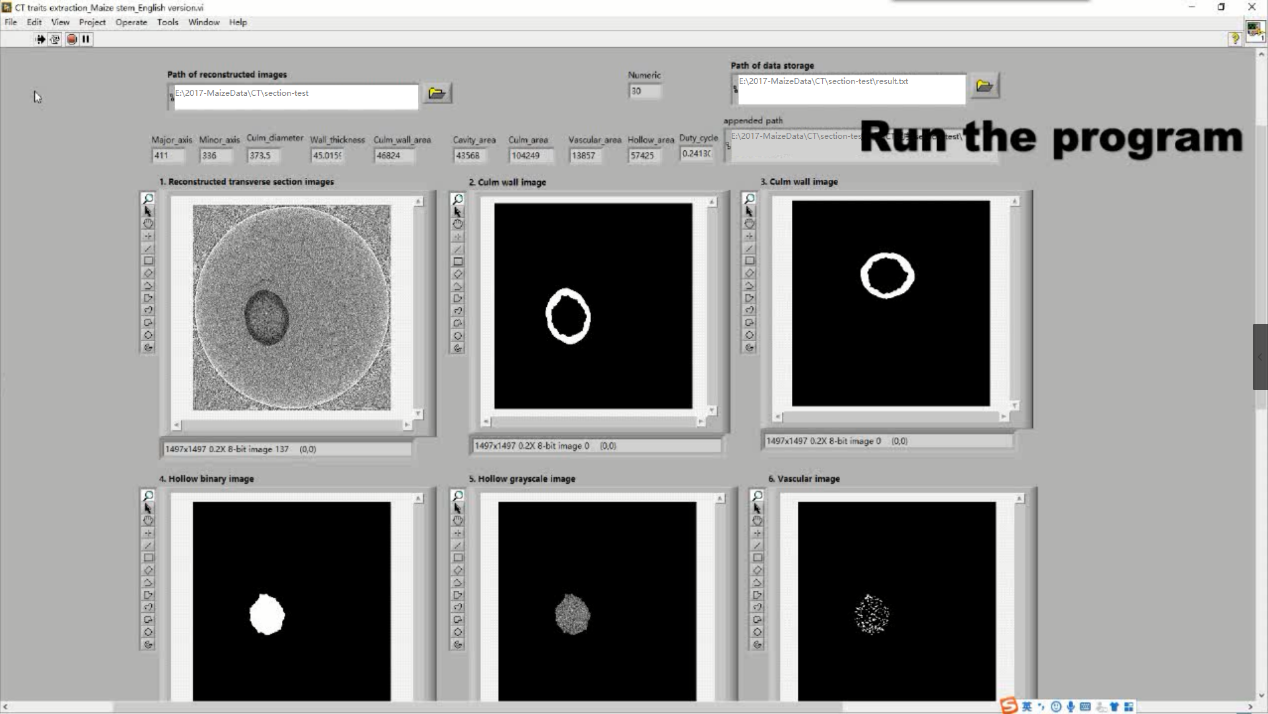


Figure 20 Running the program


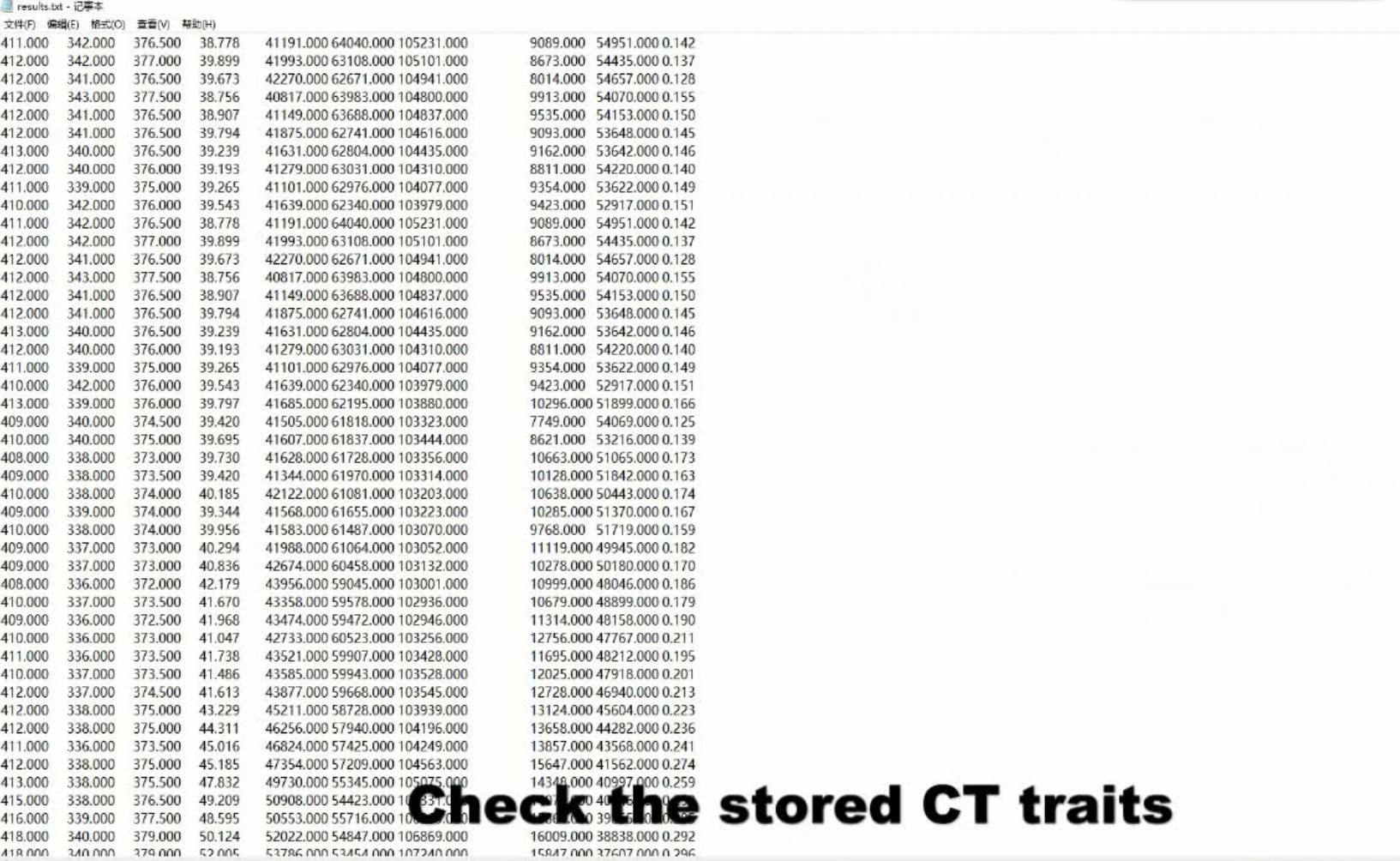


Figure 21 Check the stored CT i-traits

**Operation guide for RGB data processing program**

More intuitive information can be found in the Supplemental Video 4

Program running environment: Windows 64 bit, LabVIEW2015 32 bit

1. **Overview of program interface**

The software interface of the side program is shown in Figure 22. At the top of the interface are path controls. The lower part of the interface is the image display controls, and the right top side of the interface is the ROI control. The lower right side of the interface is part of the calculated i-traits.


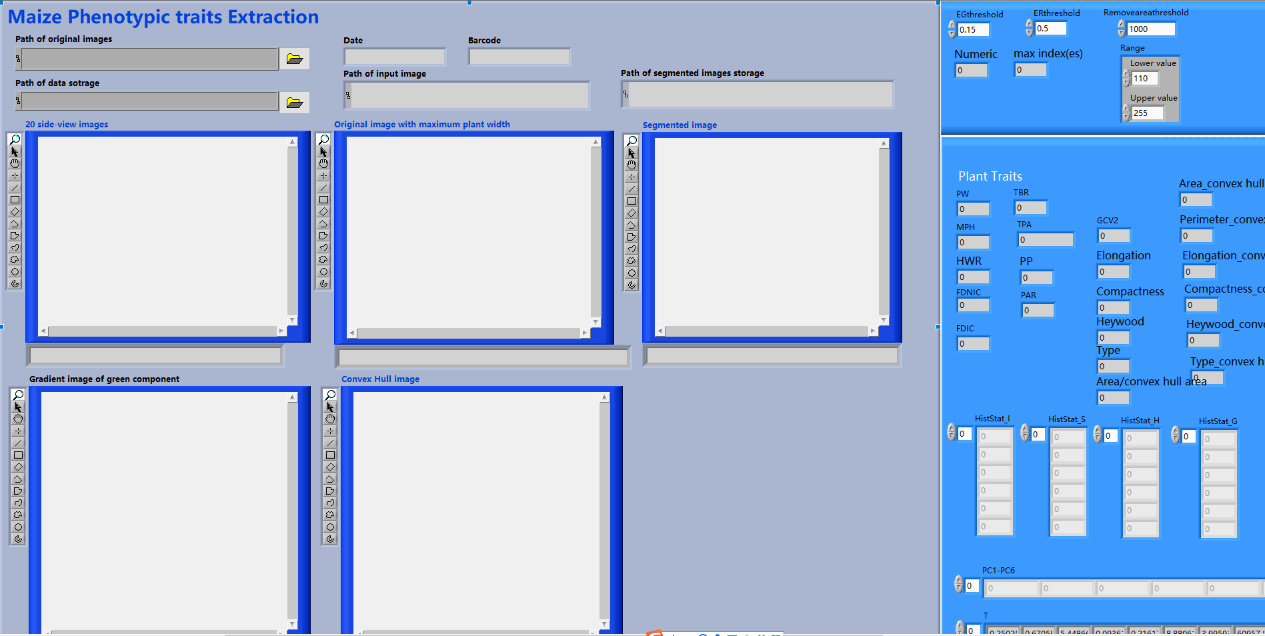


Figure 22 The software interface of the RGB data processing program

1. **Opening the “Maize traits Extraction_English version.vi” and setting the path of input images and stored results.**


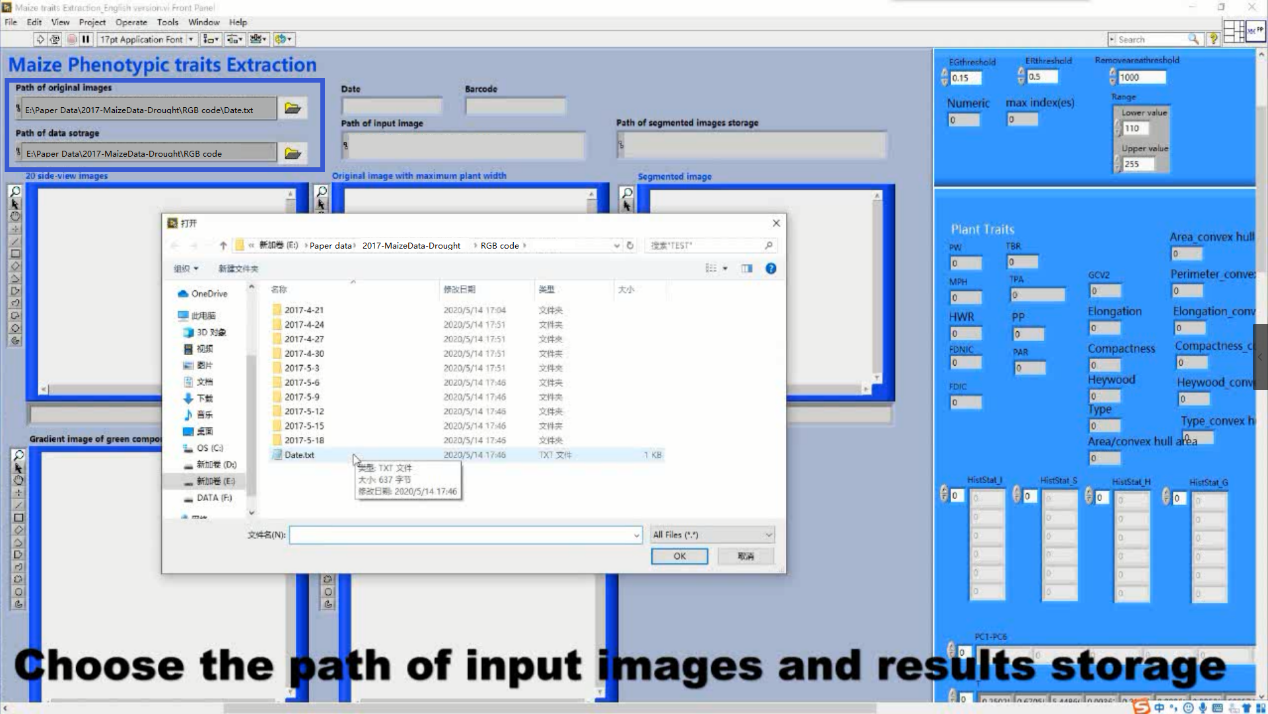


Figure 22 Setting the path of input images and stored results.

1. **Running the program.**


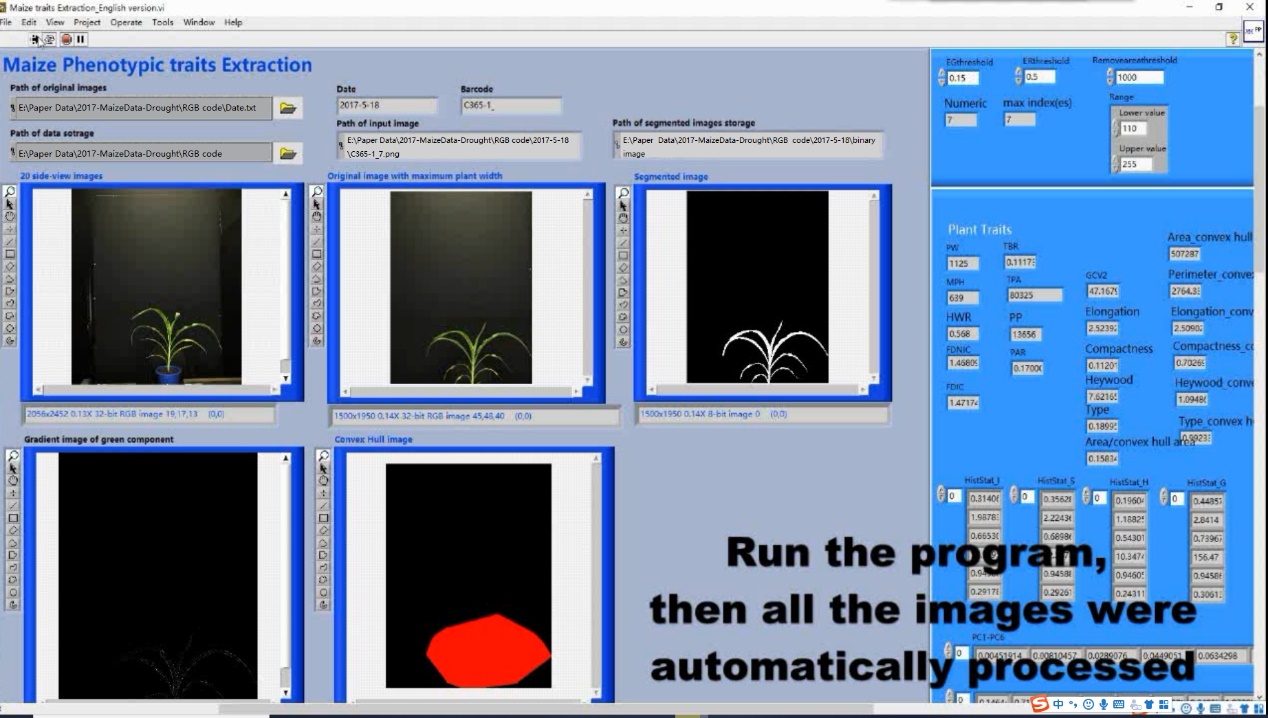


Figure 23 Running the program.

1. **After all the images are processed, checking the stored results.**


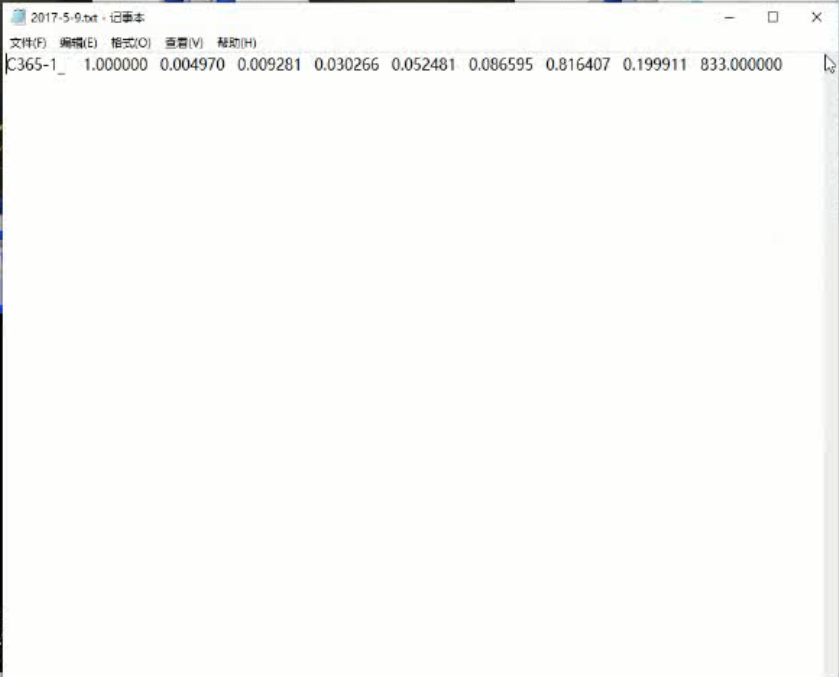


Figure 24 Results file.

**Operation guide for filtering outliers**

More intuitive information can be found in the Supplemental Video 6

Program running environment: Windows 64 bit, LabVIEW2015

1. **Opening the “filtering outliers.vi” and setting the path of folder of input files.**

**
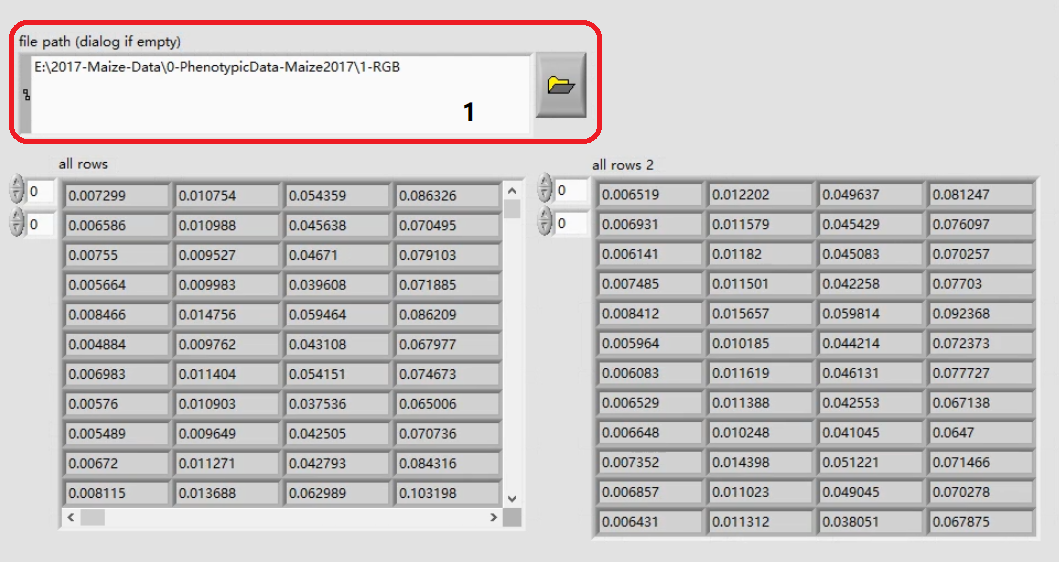
**

**Figure 25 The interface of the program “filtering outliers.vi”**

1. **Set parameters. The red box 1 in Figure 25 represents the path of the i-traits.**
2. **Press “Ctrl+R” to run the program**
3. **When the program finished, the results of the image segmentation were saved in a separate folder called “**** filtering outliers”**


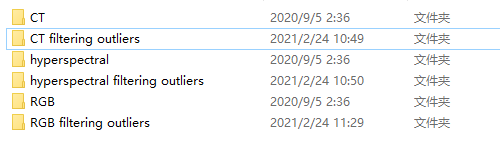


**Figure 26 The result folder**

**Operation guide for T-tests**

More intuitive information can be found in the Supplemental Video 7

Program running environment: Windows 64 bit, IBM SPSS Statistics 25

1. **Opening the i-traits data with IBM SPSS Statistics 25. The i-traits of WW was marked with “1” and the i-traits of DS was marked with “2”**


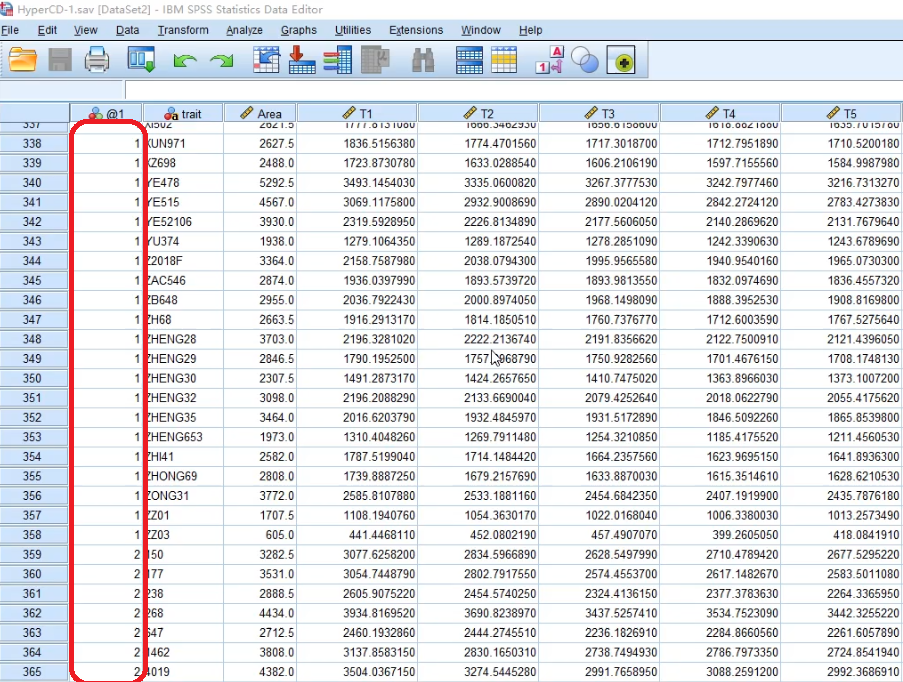


**Figure 27 The i-traits data with IBM SPSS Statistics 25**

1. **Clicking on the content as shown in the following figure**


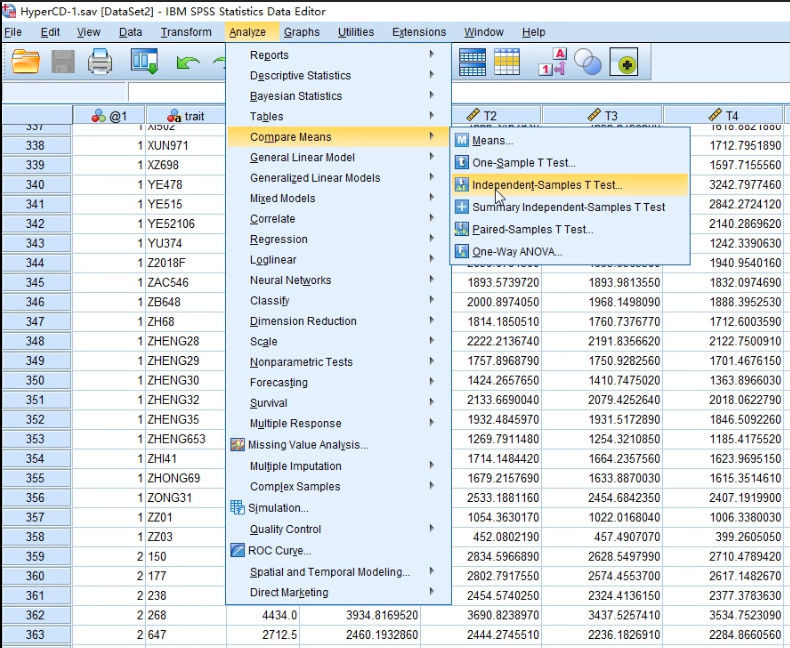


**Figure 28 Steps of T-tests**

1. **Screening i-traits with significant differences based on the results of Red box**


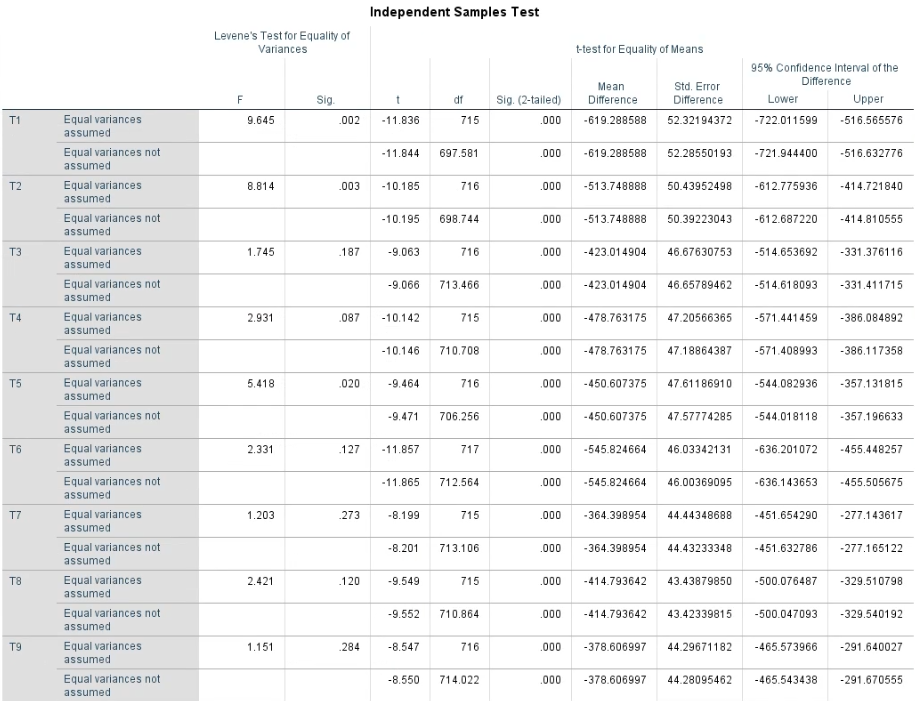


**Figure 29 Results of T-tests**

**Operation guide for multilayer perceptron (MLP)**

More intuitive information can be found in the Supplemental Video 8

Program running environment: Windows 64 bit, IBM SPSS Statistics 25

1. Opening the i-traits data with IBM SPSS Statistics 25. The i-traits of WW was marked with “1” and the i-traits of DS was marked with “2”


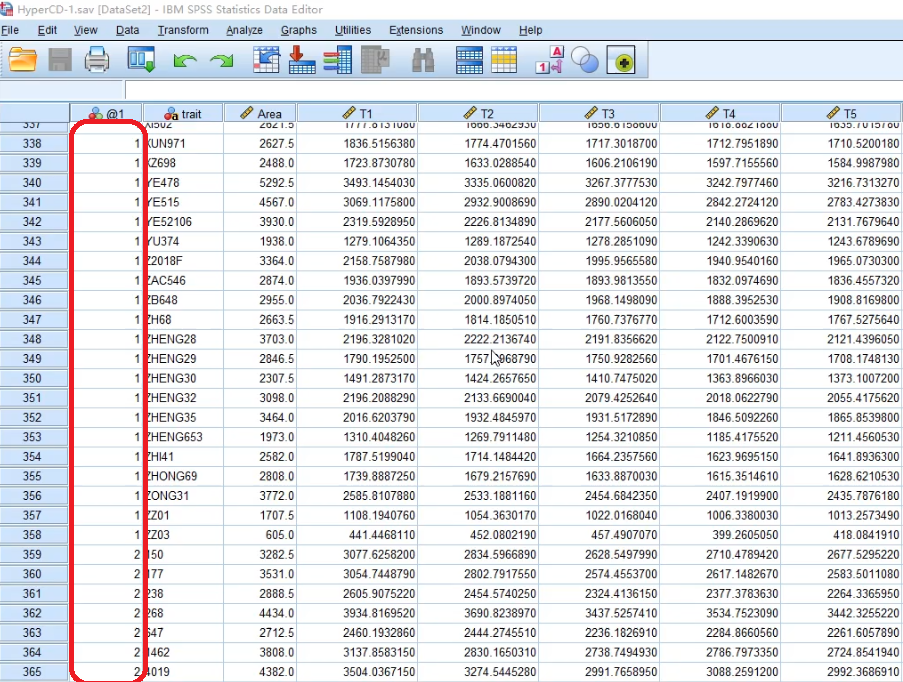


**Figure 30 The i-traits data with IBM SPSS Statistics 25**

1. **Clicking on the content as shown in the following figure**


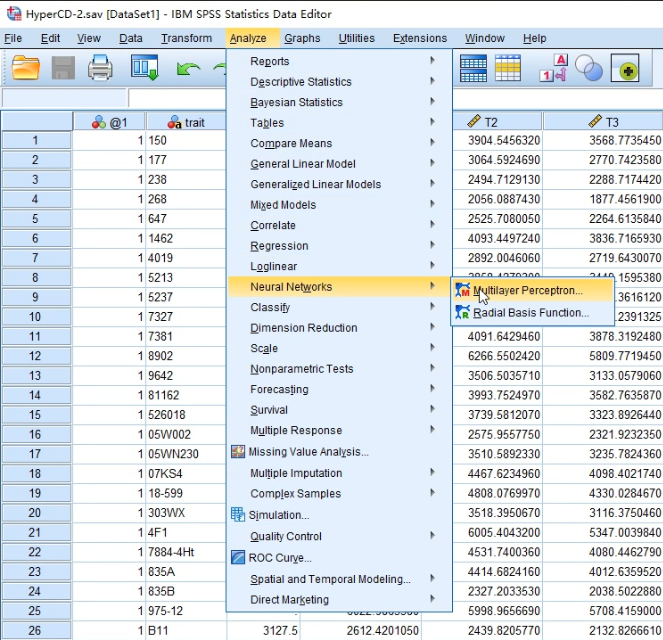


**Figure 31 Steps of MLP**

1. **Screening i-traits with significant differences based on the results of MLP**


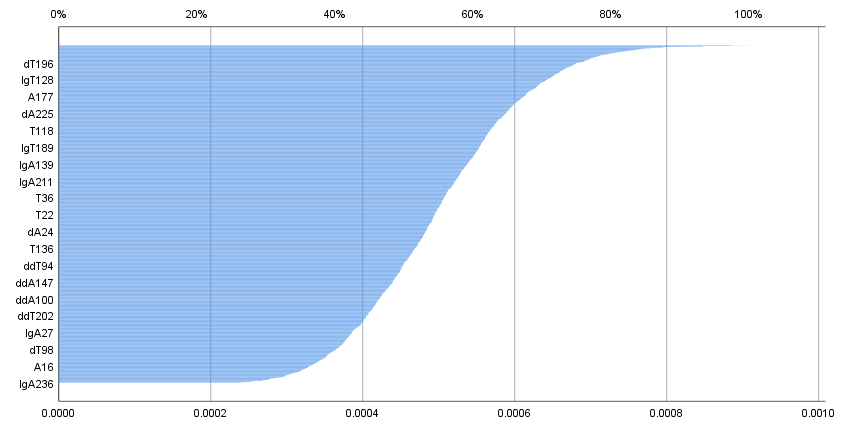


**Figure 32 Results of MLP**

**Operation guide for calculation of heritability**

**The heritability was calculated using the lmer function in the lme4 package in the R environment**


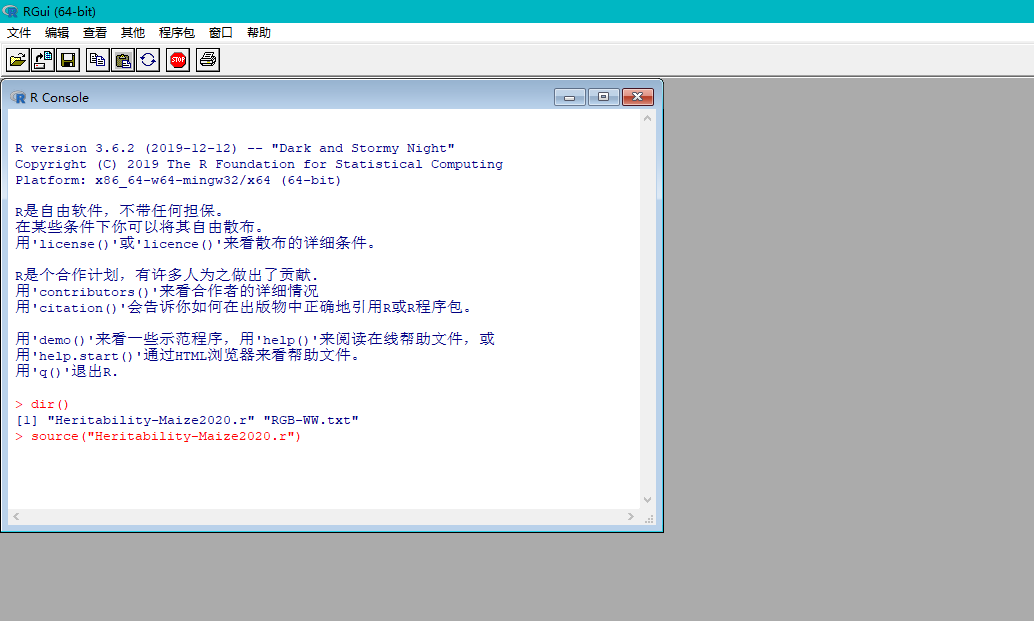


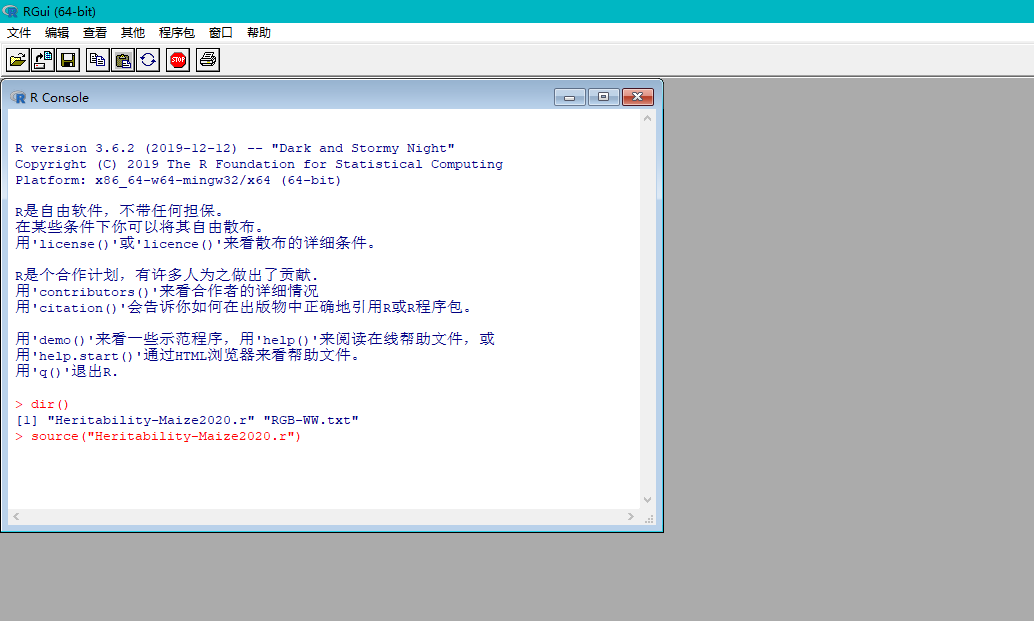


**Figure 33 Calculation of heritability**


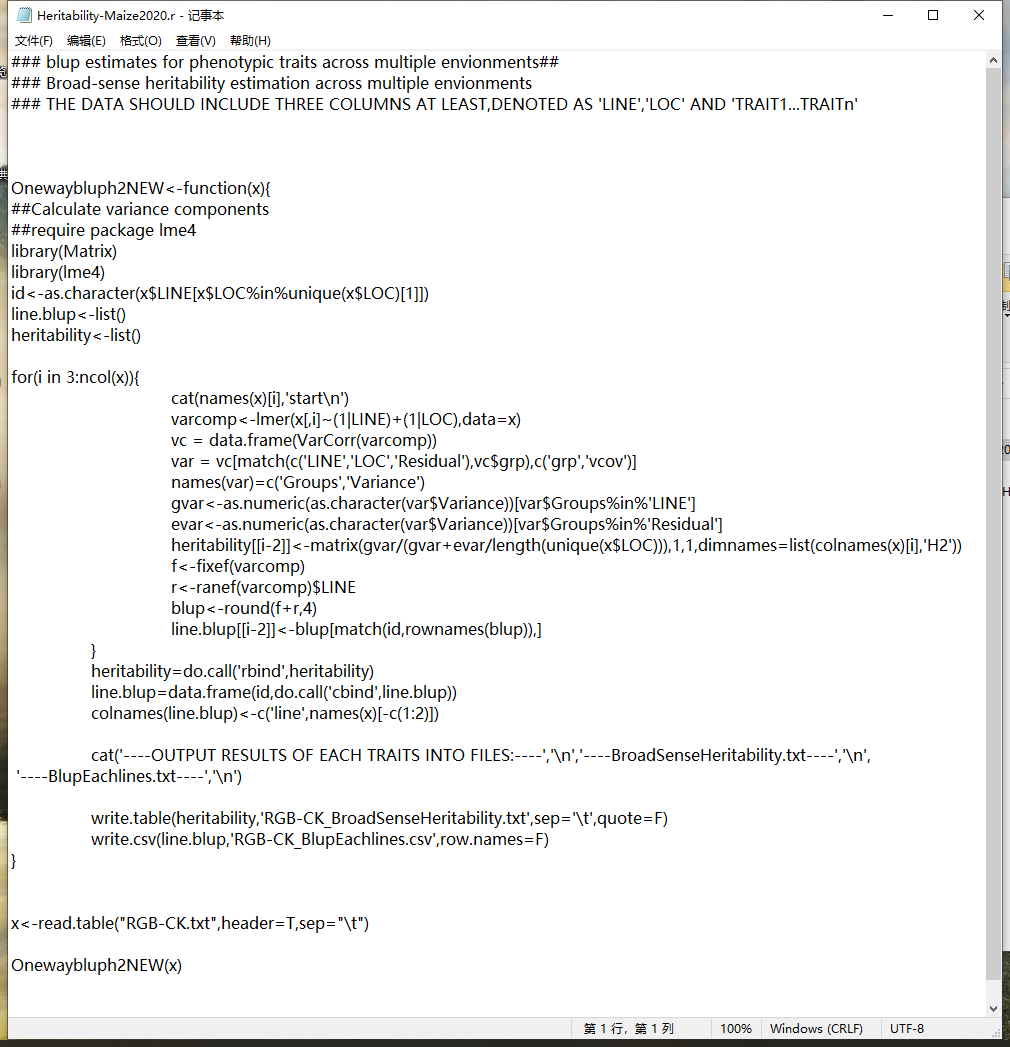


**Figure 34 R-code of heritability**

**Note:**

1. **More intuitive information can be obtained in the Supplemental Video 2-4 and 6-8.**
2. **The code of CT, HSI and RGB image analysis pipelines could be downloaded via the link: https://github.com/fenghuifh2006/Maize-RGB-CT-HSI-program and https://doi.org/10.5281/zenodo.4690730. (The files which the size greater than 25M can be download in the red box)**


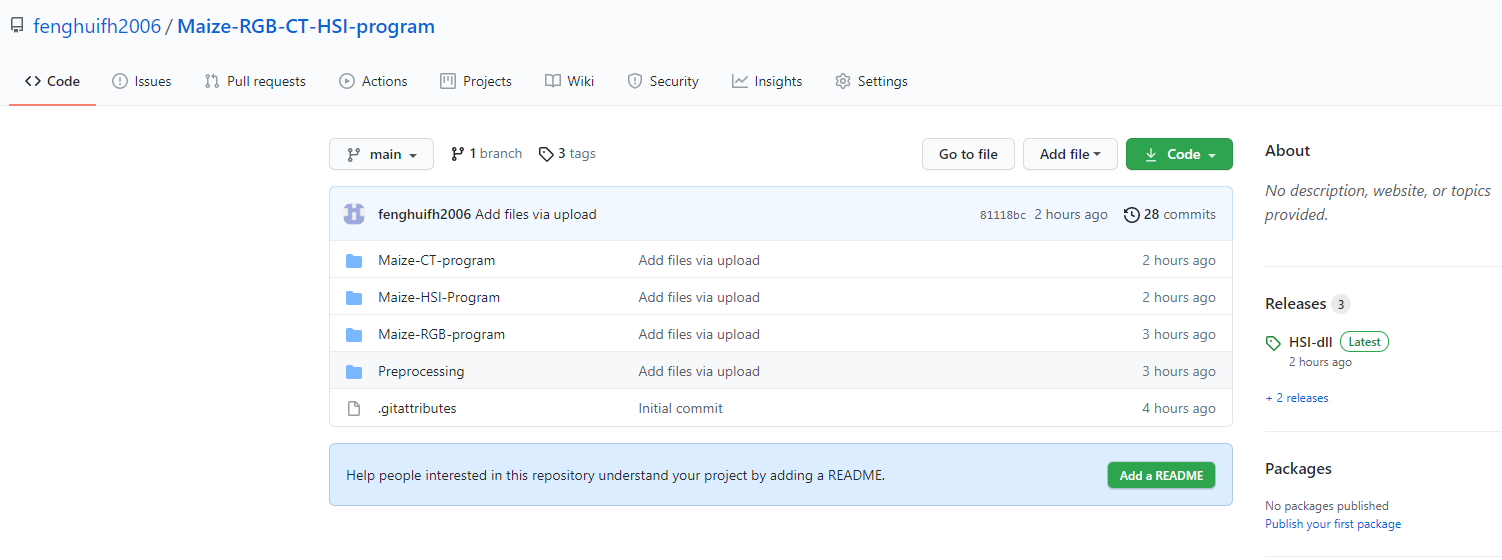


1. **If you have questions about running our program (such as calling the dynamic link library, dll files), please contact Wanneng Yang (ywn@mail.hzau.edu.cn)**
