# Supplementary material for: Using high-throughput multiple optical phenotyping to decipher the genetic architecture of maize drought tolerance
Source: Genome Biol. 2021 Jun 24;22:185. doi: 10.1186/s13059-021-02377-0 (PMC8223302; doi:10.1186/s13059-021-02377-0)
Supplement: Supplementary file 10 — Additional file 10: Figures S1-S11. [file 13059_2021_2377_MOESM10_ESM.pptx]

## Slide 1
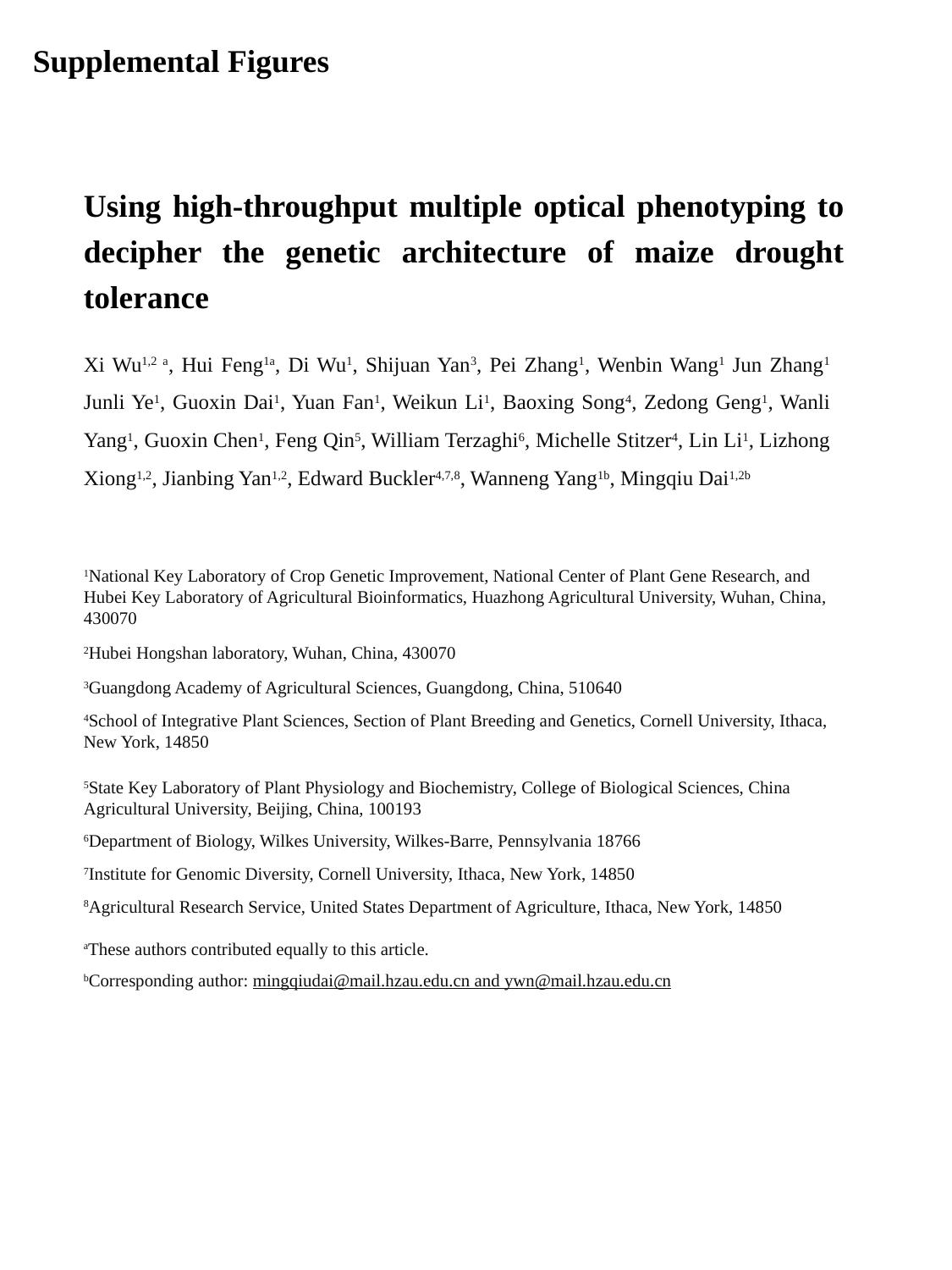

Supplemental Figures
Using high-throughput multiple optical phenotyping to decipher the genetic architecture of maize drought tolerance
Xi Wu1,2 a, Hui Feng1a, Di Wu1, Shijuan Yan3, Pei Zhang1, Wenbin Wang1 Jun Zhang1 Junli Ye1, Guoxin Dai1, Yuan Fan1, Weikun Li1, Baoxing Song4, Zedong Geng1, Wanli Yang1, Guoxin Chen1, Feng Qin5, William Terzaghi6, Michelle Stitzer4, Lin Li1, Lizhong Xiong1,2, Jianbing Yan1,2, Edward Buckler4,7,8, Wanneng Yang1b, Mingqiu Dai1,2b
1National Key Laboratory of Crop Genetic Improvement, National Center of Plant Gene Research, and Hubei Key Laboratory of Agricultural Bioinformatics, Huazhong Agricultural University, Wuhan, China, 430070
2Hubei Hongshan laboratory, Wuhan, China, 430070
3Guangdong Academy of Agricultural Sciences, Guangdong, China, 510640
4School of Integrative Plant Sciences, Section of Plant Breeding and Genetics, Cornell University, Ithaca, New York, 14850
5State Key Laboratory of Plant Physiology and Biochemistry, College of Biological Sciences, China Agricultural University, Beijing, China, 100193
6Department of Biology, Wilkes University, Wilkes-Barre, Pennsylvania 18766
7Institute for Genomic Diversity, Cornell University, Ithaca, New York, 14850
8Agricultural Research Service, United States Department of Agriculture, Ithaca, New York, 14850
aThese authors contributed equally to this article.
bCorresponding author: mingqiudai@mail.hzau.edu.cn and ywn@mail.hzau.edu.cn

## Slide 2
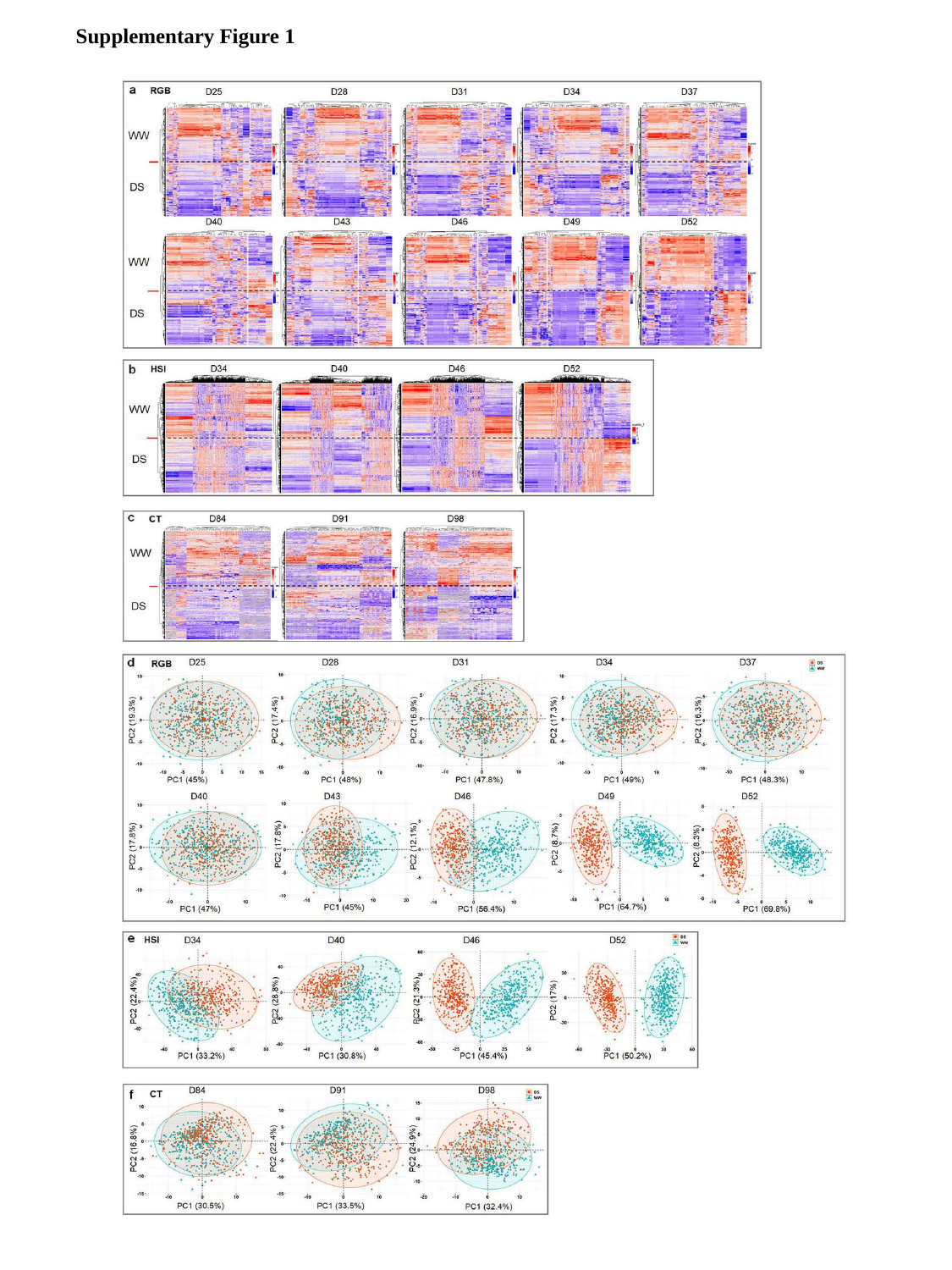

Supplementary Figure 1

## Slide 3
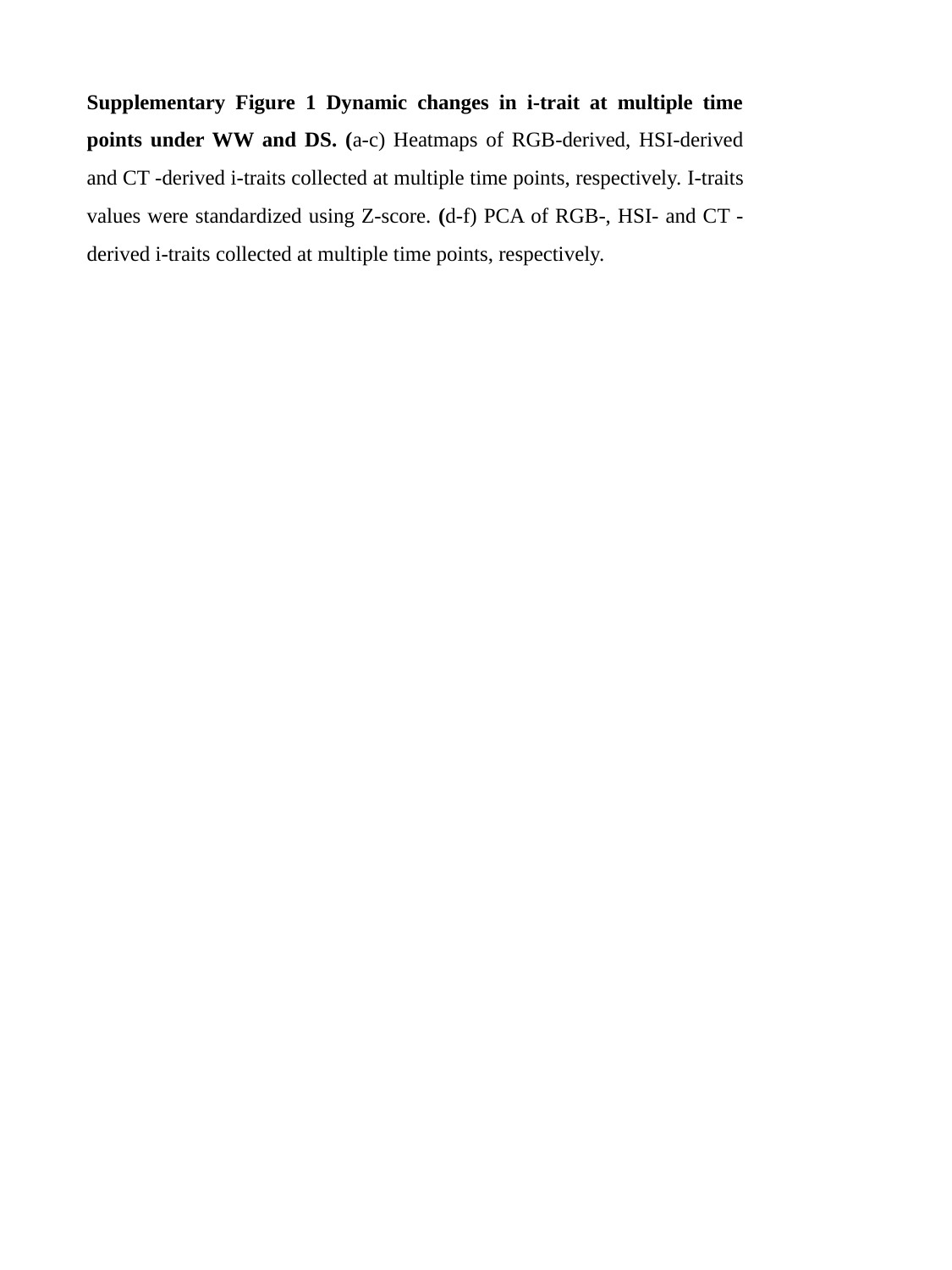

Supplementary Figure 1 Dynamic changes in i-trait at multiple time points under WW and DS. (a-c) Heatmaps of RGB-derived, HSI-derived and CT -derived i-traits collected at multiple time points, respectively. I-traits values were standardized using Z-score. (d-f) PCA of RGB-, HSI- and CT -derived i-traits collected at multiple time points, respectively.

## Slide 4
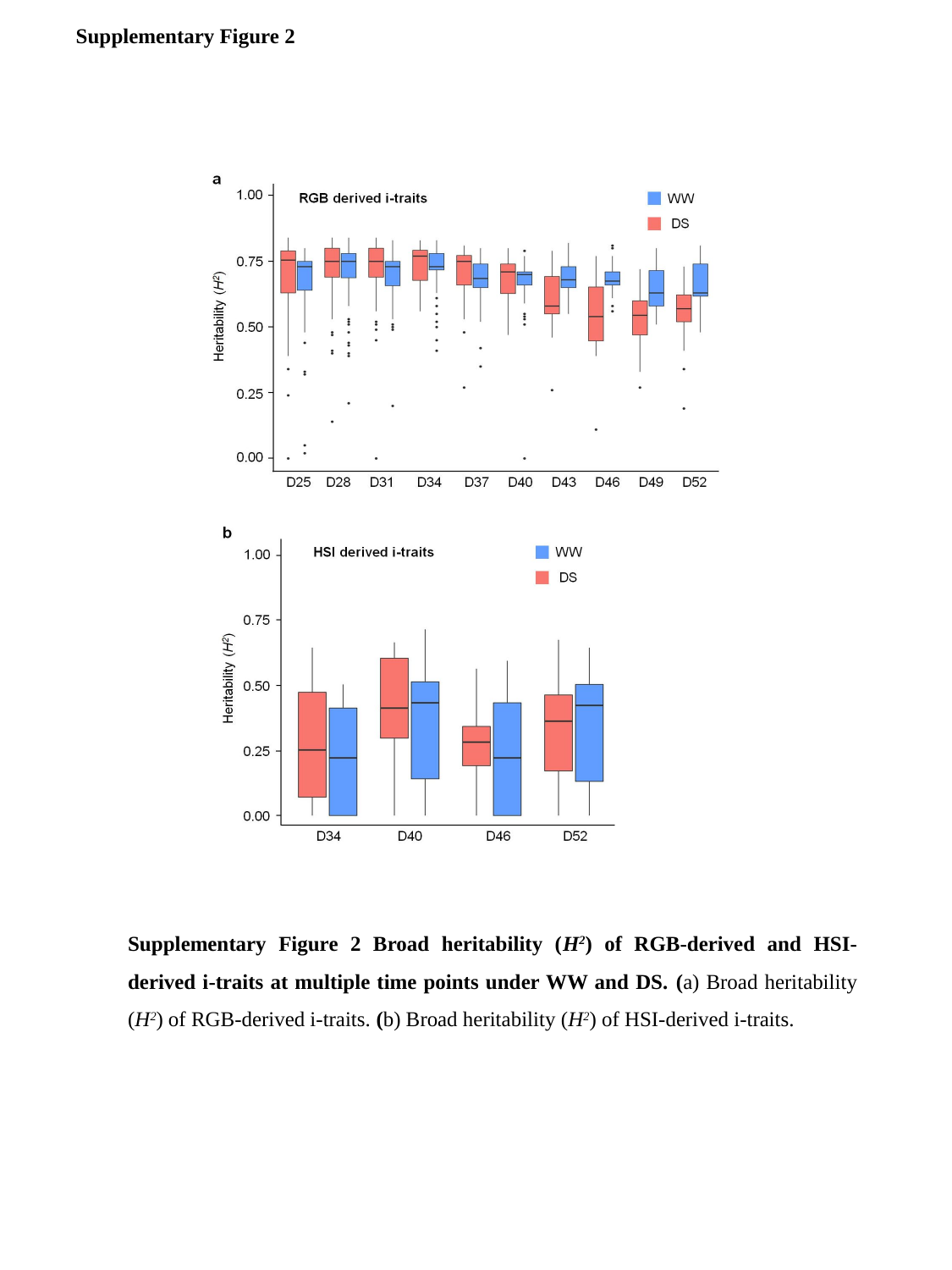

Supplementary Figure 2
Supplementary Figure 2 Broad heritability (H2) of RGB-derived and HSI-derived i-traits at multiple time points under WW and DS. (a) Broad heritability (H2) of RGB-derived i-traits. (b) Broad heritability (H2) of HSI-derived i-traits.

## Slide 5
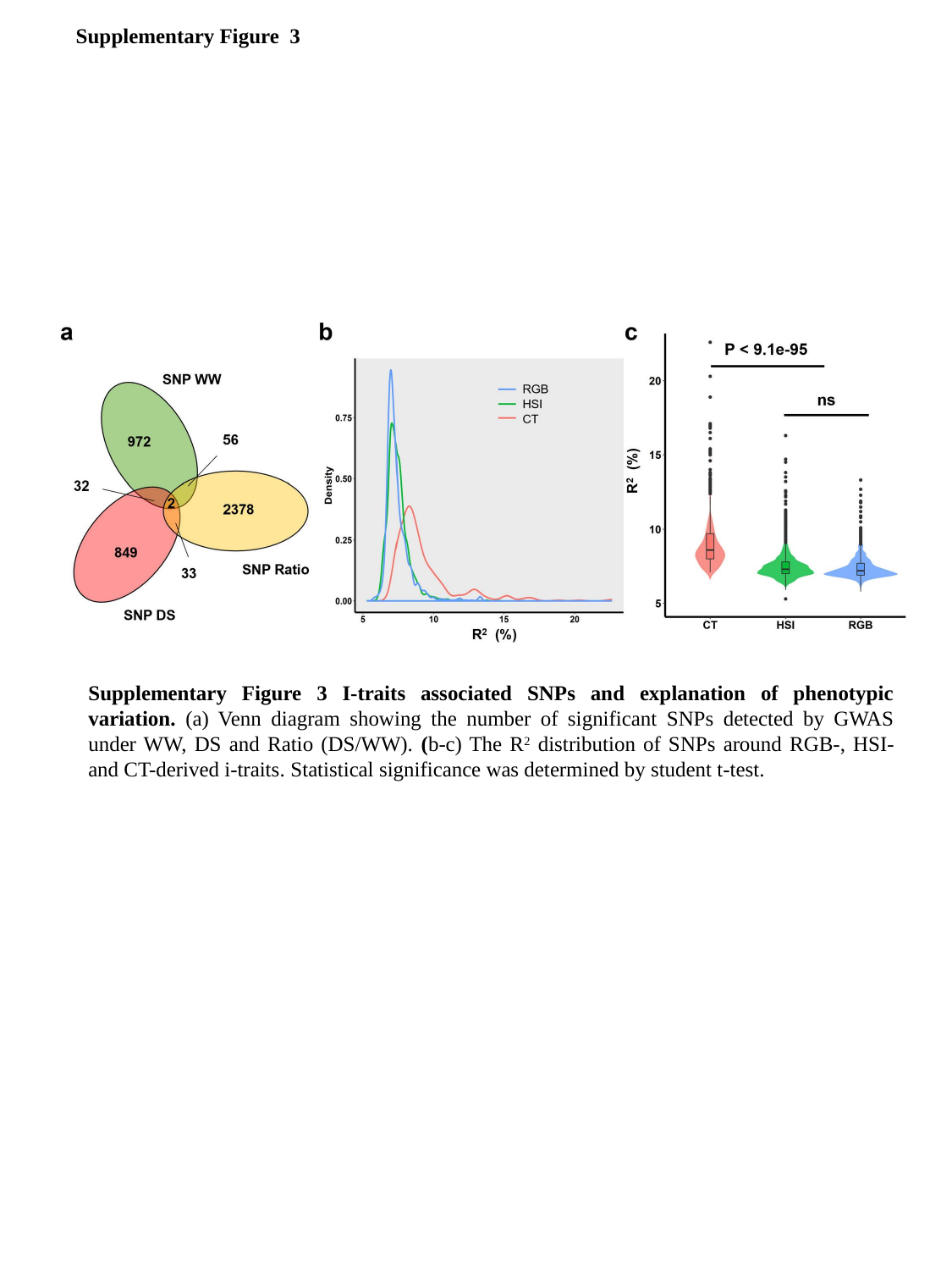

Supplementary Figure 3
Supplementary Figure 3 I-traits associated SNPs and explanation of phenotypic variation. (a) Venn diagram showing the number of significant SNPs detected by GWAS under WW, DS and Ratio (DS/WW). (b-c) The R2 distribution of SNPs around RGB-, HSI- and CT-derived i-traits. Statistical significance was determined by student t-test.

## Slide 6
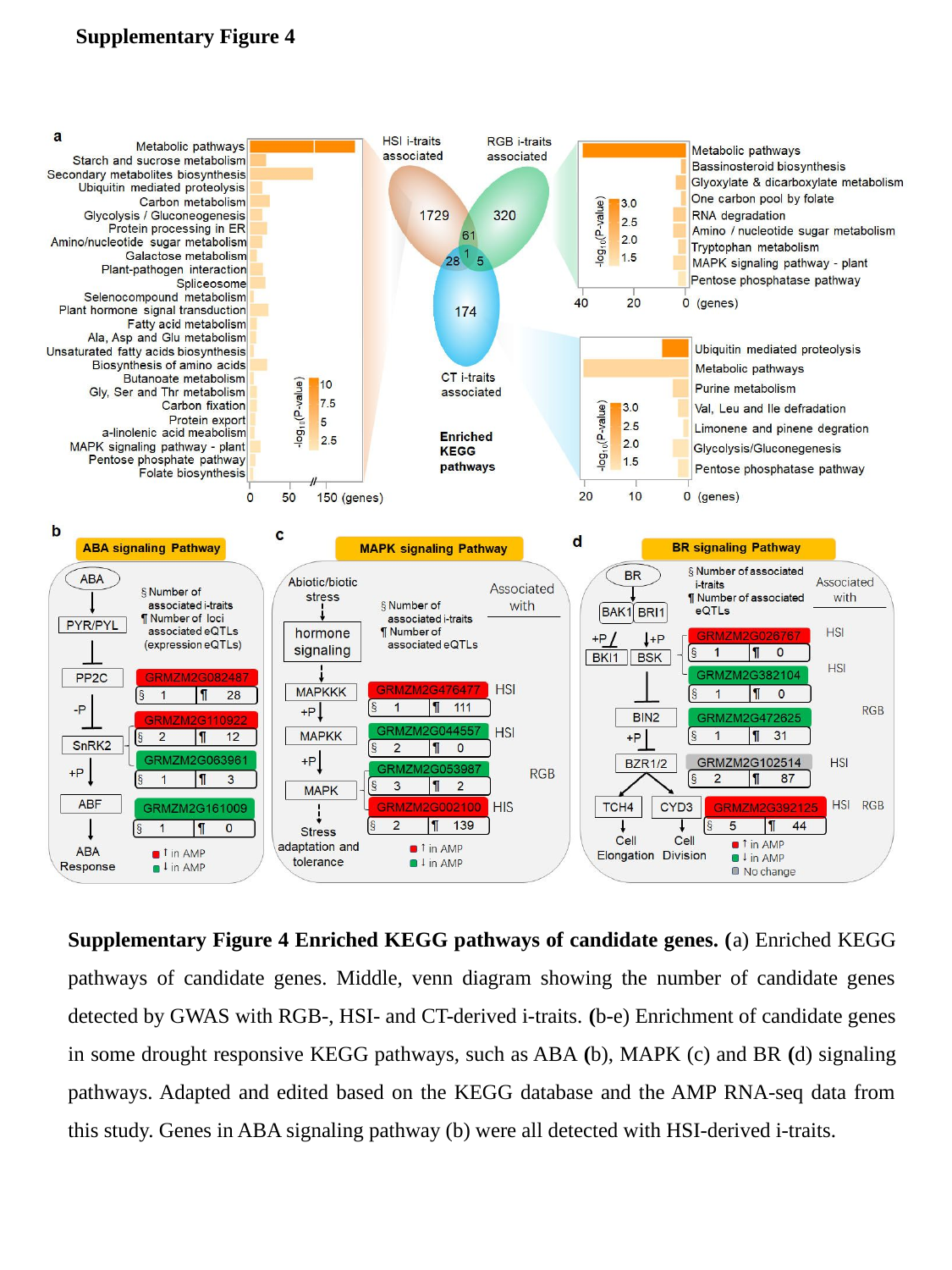

Supplementary Figure 4
Supplementary Figure 4 Enriched KEGG pathways of candidate genes. (a) Enriched KEGG pathways of candidate genes. Middle, venn diagram showing the number of candidate genes detected by GWAS with RGB-, HSI- and CT-derived i-traits. (b-e) Enrichment of candidate genes in some drought responsive KEGG pathways, such as ABA (b), MAPK (c) and BR (d) signaling pathways. Adapted and edited based on the KEGG database and the AMP RNA-seq data from this study. Genes in ABA signaling pathway (b) were all detected with HSI-derived i-traits.

## Slide 7
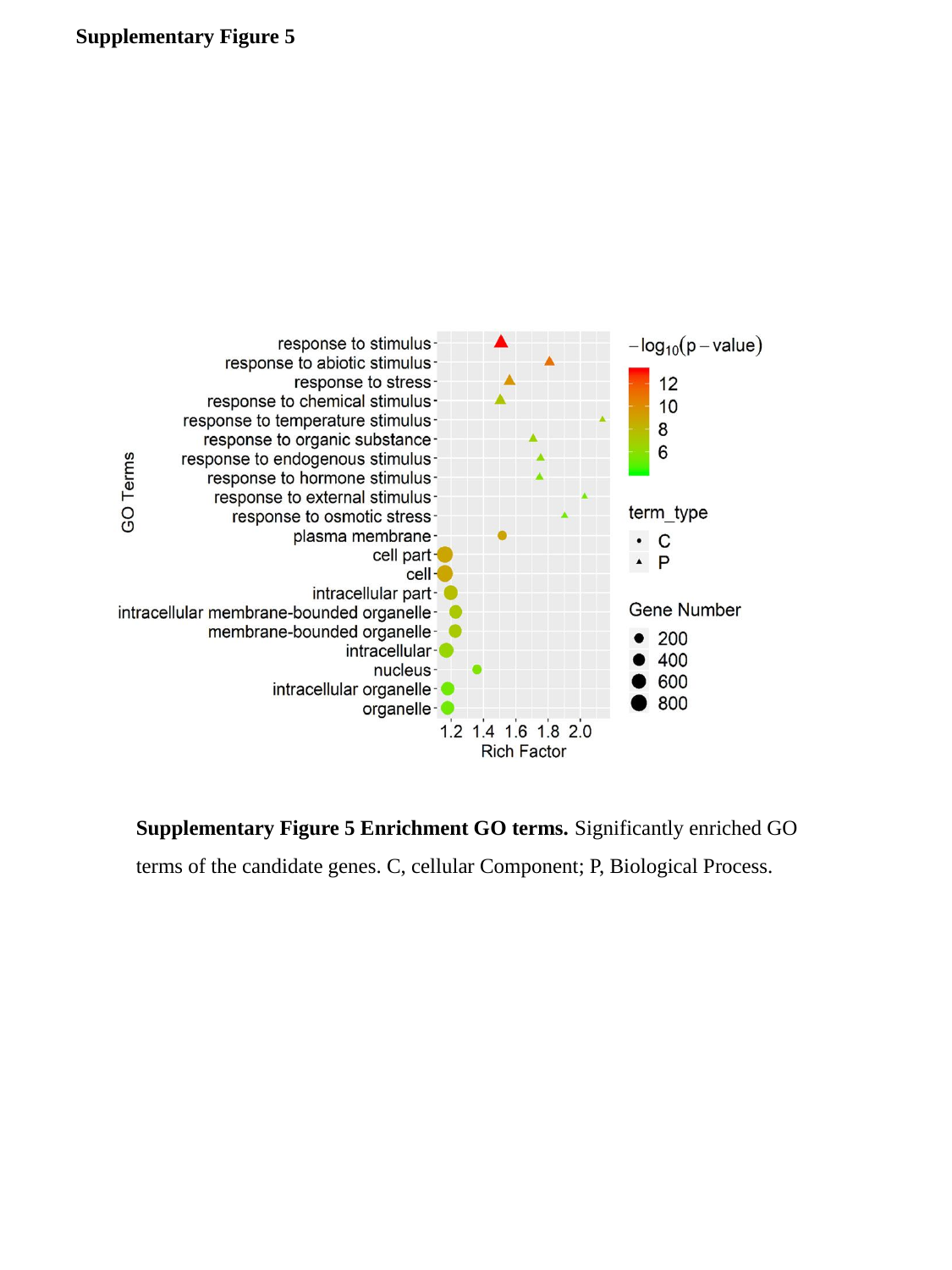

Supplementary Figure 5
Supplementary Figure 5 Enrichment GO terms. Significantly enriched GO terms of the candidate genes. C, cellular Component; P, Biological Process.

## Slide 8
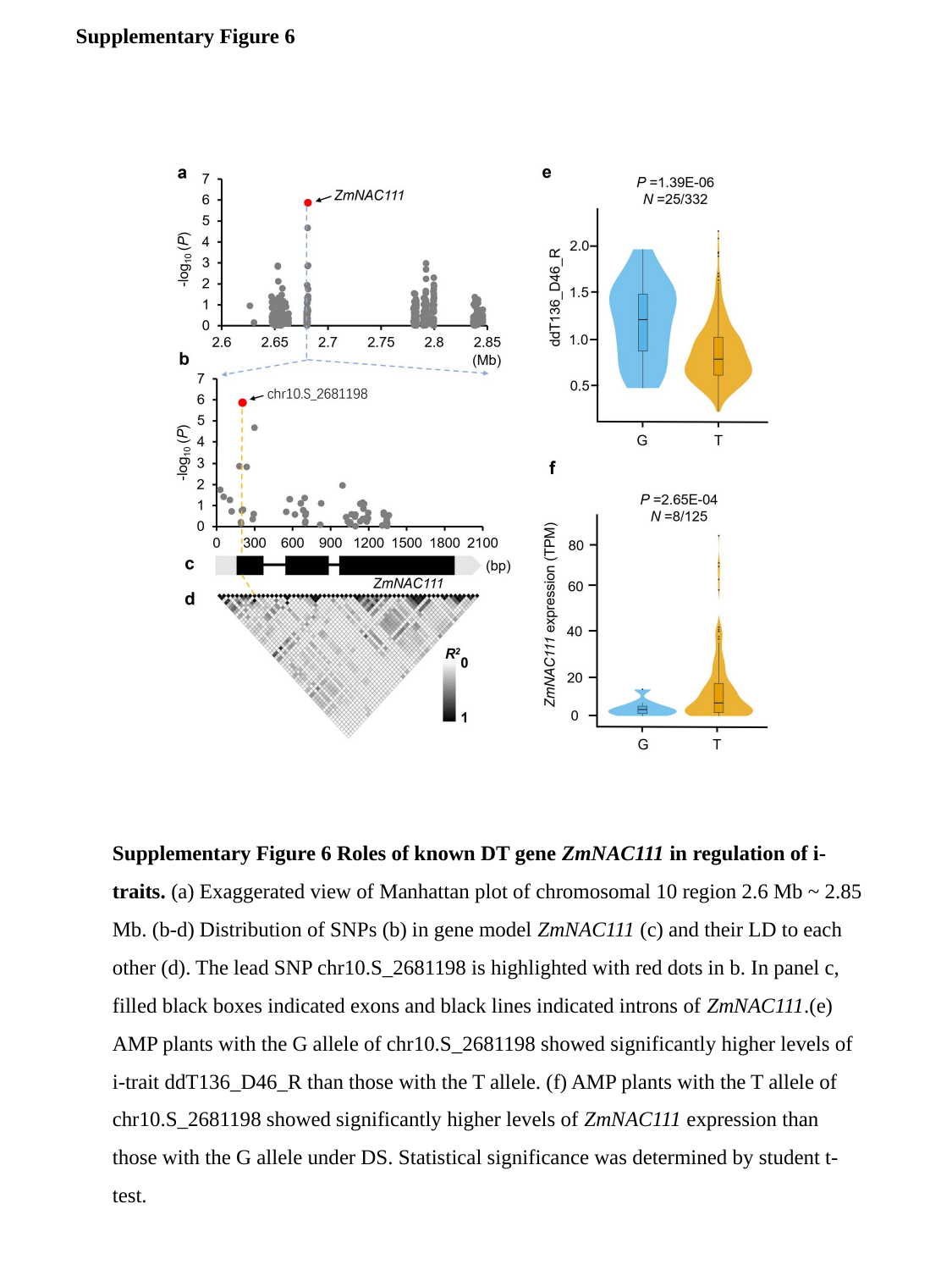

Supplementary Figure 6
Supplementary Figure 6 Roles of known DT gene ZmNAC111 in regulation of i-traits. (a) Exaggerated view of Manhattan plot of chromosomal 10 region 2.6 Mb ~ 2.85 Mb. (b-d) Distribution of SNPs (b) in gene model ZmNAC111 (c) and their LD to each other (d). The lead SNP chr10.S_2681198 is highlighted with red dots in b. In panel c, filled black boxes indicated exons and black lines indicated introns of ZmNAC111.(e) AMP plants with the G allele of chr10.S_2681198 showed significantly higher levels of i-trait ddT136_D46_R than those with the T allele. (f) AMP plants with the T allele of chr10.S_2681198 showed significantly higher levels of ZmNAC111 expression than those with the G allele under DS. Statistical significance was determined by student t-test.

## Slide 9
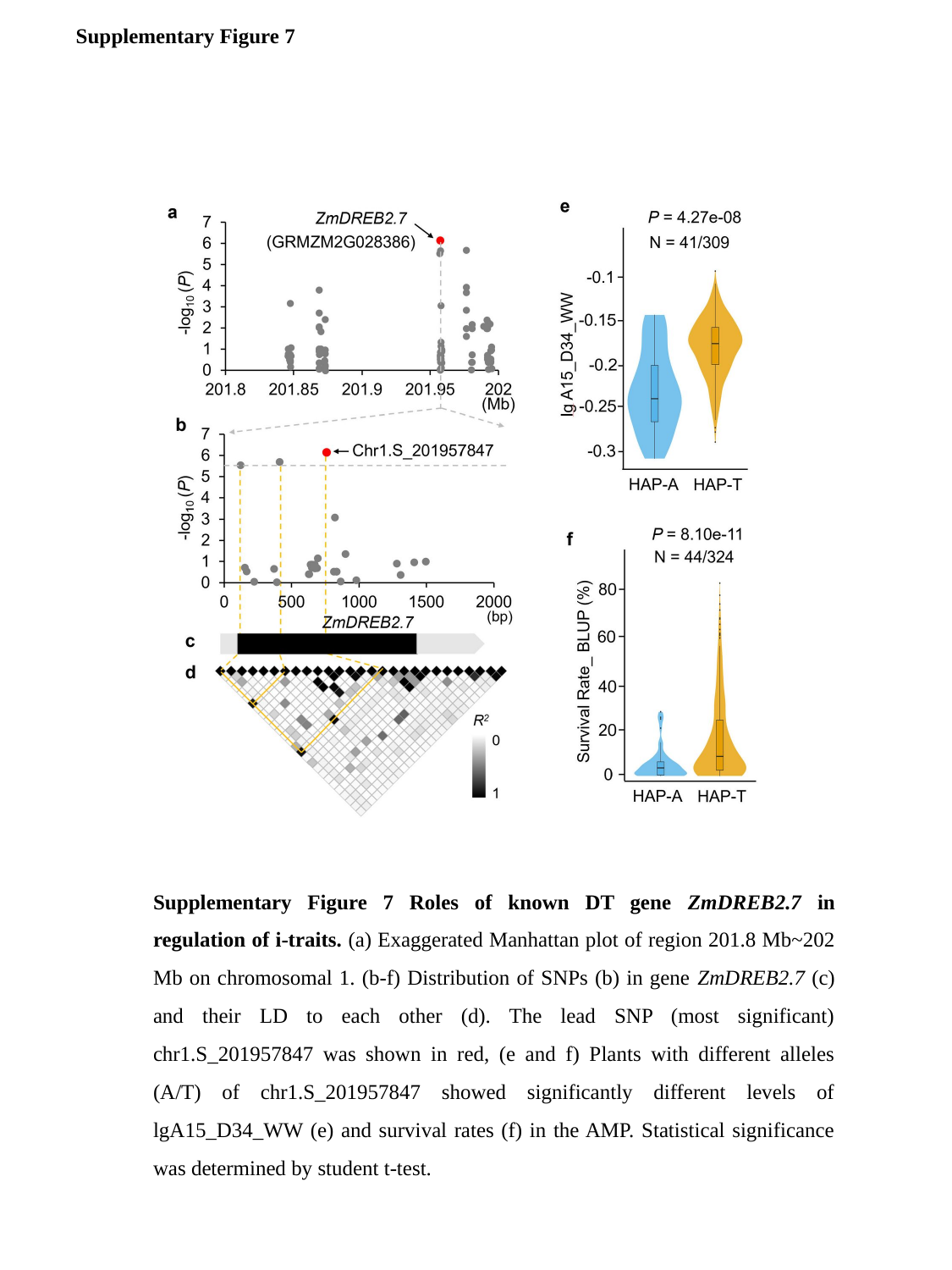

Supplementary Figure 7
Supplementary Figure 7 Roles of known DT gene ZmDREB2.7 in regulation of i-traits. (a) Exaggerated Manhattan plot of region 201.8 Mb~202 Mb on chromosomal 1. (b-f) Distribution of SNPs (b) in gene ZmDREB2.7 (c) and their LD to each other (d). The lead SNP (most significant) chr1.S_201957847 was shown in red, (e and f) Plants with different alleles (A/T) of chr1.S_201957847 showed significantly different levels of lgA15_D34_WW (e) and survival rates (f) in the AMP. Statistical significance was determined by student t-test.

## Slide 10
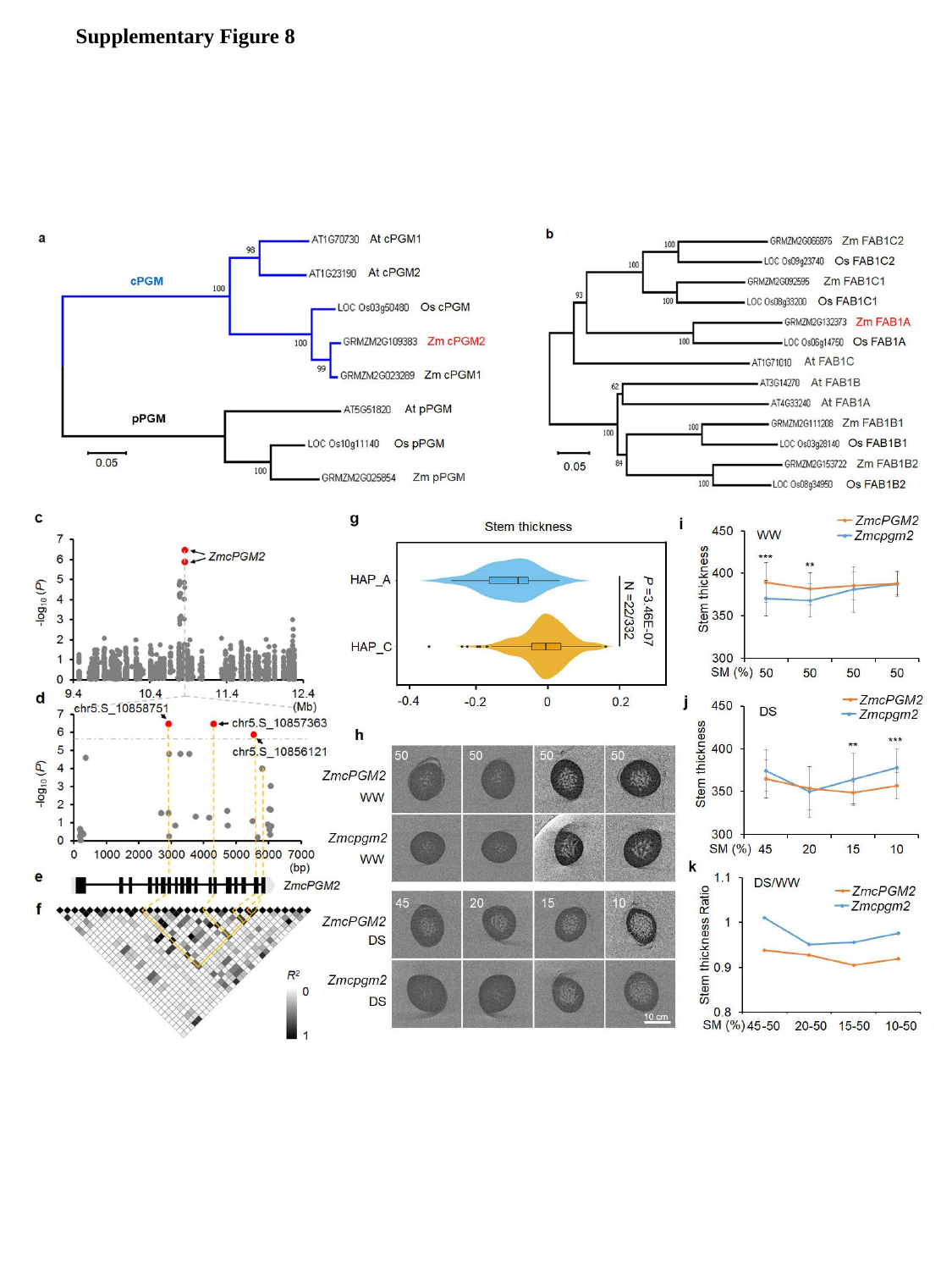

Supplementary Figure 8

## Slide 11
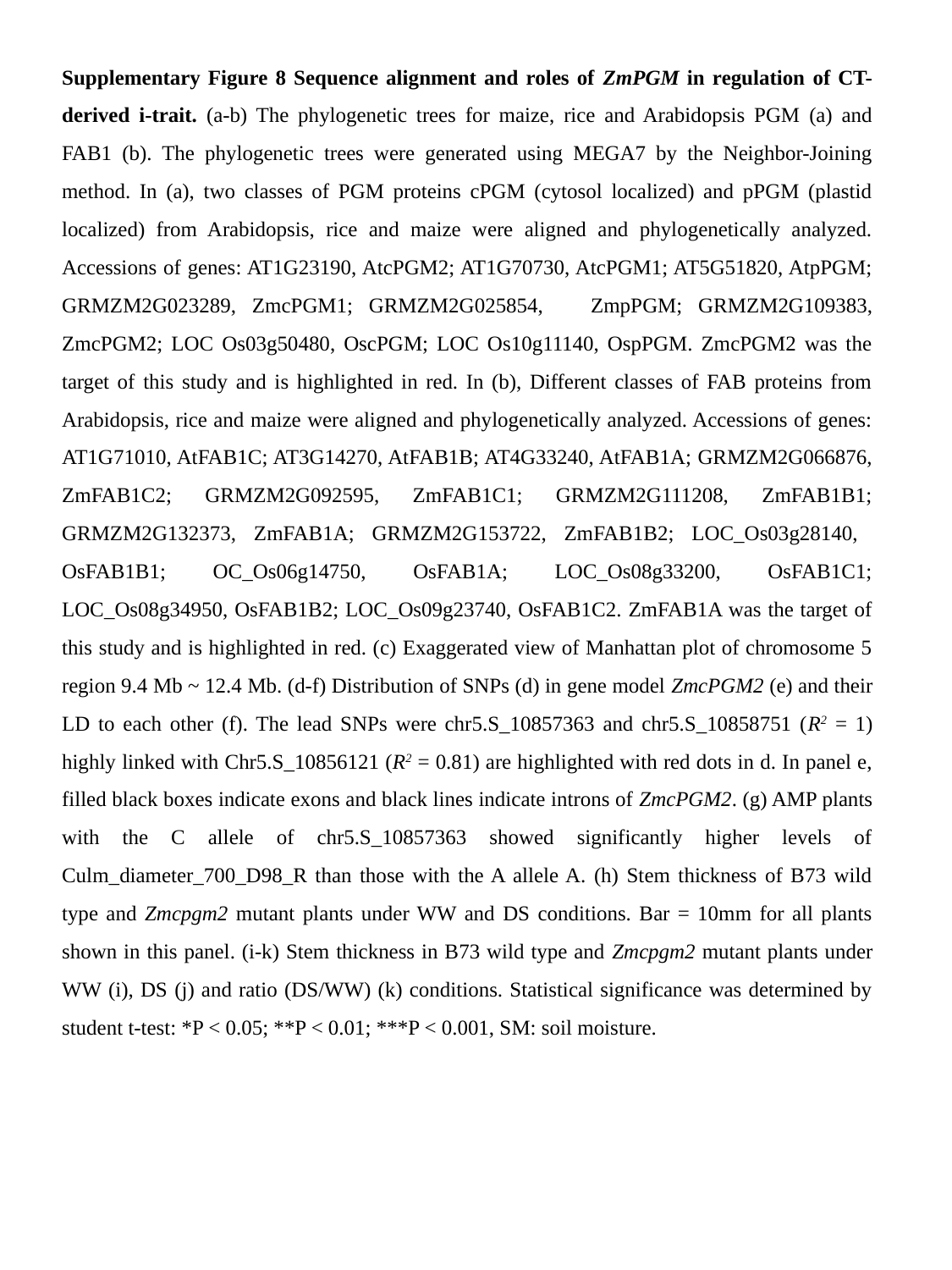

Supplementary Figure 8 Sequence alignment and roles of ZmPGM in regulation of CT-derived i-trait. (a-b) The phylogenetic trees for maize, rice and Arabidopsis PGM (a) and FAB1 (b). The phylogenetic trees were generated using MEGA7 by the Neighbor-Joining method. In (a), two classes of PGM proteins cPGM (cytosol localized) and pPGM (plastid localized) from Arabidopsis, rice and maize were aligned and phylogenetically analyzed. Accessions of genes: AT1G23190, AtcPGM2; AT1G70730, AtcPGM1; AT5G51820, AtpPGM; GRMZM2G023289, ZmcPGM1; GRMZM2G025854,	ZmpPGM; GRMZM2G109383, ZmcPGM2; LOC Os03g50480, OscPGM; LOC Os10g11140, OspPGM. ZmcPGM2 was the target of this study and is highlighted in red. In (b), Different classes of FAB proteins from Arabidopsis, rice and maize were aligned and phylogenetically analyzed. Accessions of genes: AT1G71010, AtFAB1C; AT3G14270, AtFAB1B; AT4G33240, AtFAB1A; GRMZM2G066876, ZmFAB1C2; GRMZM2G092595, ZmFAB1C1; GRMZM2G111208, ZmFAB1B1; GRMZM2G132373, ZmFAB1A; GRMZM2G153722, ZmFAB1B2; LOC_Os03g28140,	OsFAB1B1; OC_Os06g14750, OsFAB1A; LOC_Os08g33200, OsFAB1C1; LOC_Os08g34950, OsFAB1B2; LOC_Os09g23740, OsFAB1C2. ZmFAB1A was the target of this study and is highlighted in red. (c) Exaggerated view of Manhattan plot of chromosome 5 region 9.4 Mb ~ 12.4 Mb. (d-f) Distribution of SNPs (d) in gene model ZmcPGM2 (e) and their LD to each other (f). The lead SNPs were chr5.S_10857363 and chr5.S_10858751 (R2 = 1) highly linked with Chr5.S_10856121 (R2 = 0.81) are highlighted with red dots in d. In panel e, filled black boxes indicate exons and black lines indicate introns of ZmcPGM2. (g) AMP plants with the C allele of chr5.S_10857363 showed significantly higher levels of Culm_diameter_700_D98_R than those with the A allele A. (h) Stem thickness of B73 wild type and Zmcpgm2 mutant plants under WW and DS conditions. Bar = 10mm for all plants shown in this panel. (i-k) Stem thickness in B73 wild type and Zmcpgm2 mutant plants under WW (i), DS (j) and ratio (DS/WW) (k) conditions. Statistical significance was determined by student t-test: *P < 0.05; **P < 0.01; ***P < 0.001, SM: soil moisture.

## Slide 12
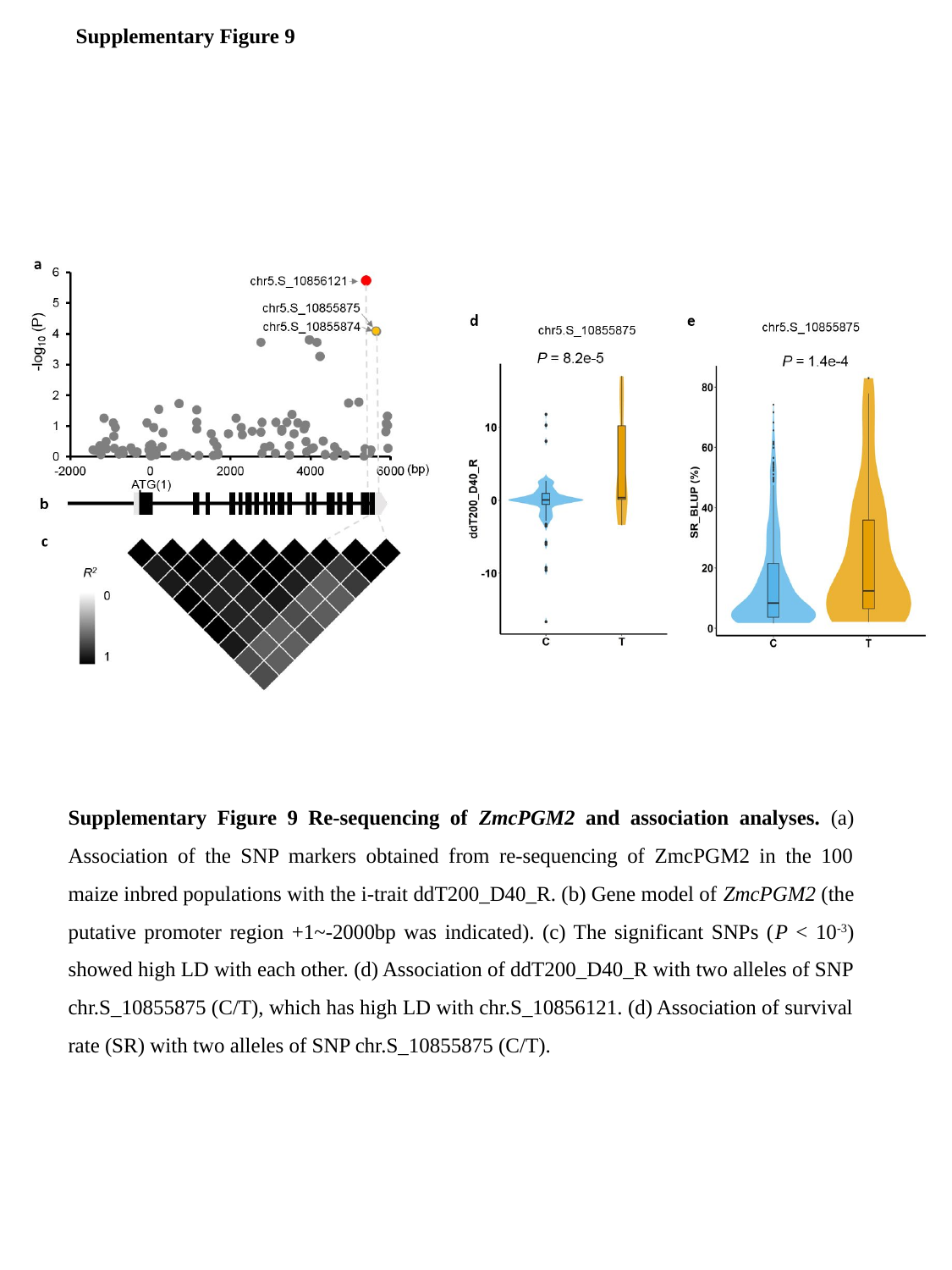

Supplementary Figure 9
Supplementary Figure 9 Re-sequencing of ZmcPGM2 and association analyses. (a) Association of the SNP markers obtained from re-sequencing of ZmcPGM2 in the 100 maize inbred populations with the i-trait ddT200_D40_R. (b) Gene model of ZmcPGM2 (the putative promoter region +1~-2000bp was indicated). (c) The significant SNPs (P < 10-3) showed high LD with each other. (d) Association of ddT200_D40_R with two alleles of SNP chr.S_10855875 (C/T), which has high LD with chr.S_10856121. (d) Association of survival rate (SR) with two alleles of SNP chr.S_10855875 (C/T).

## Slide 13
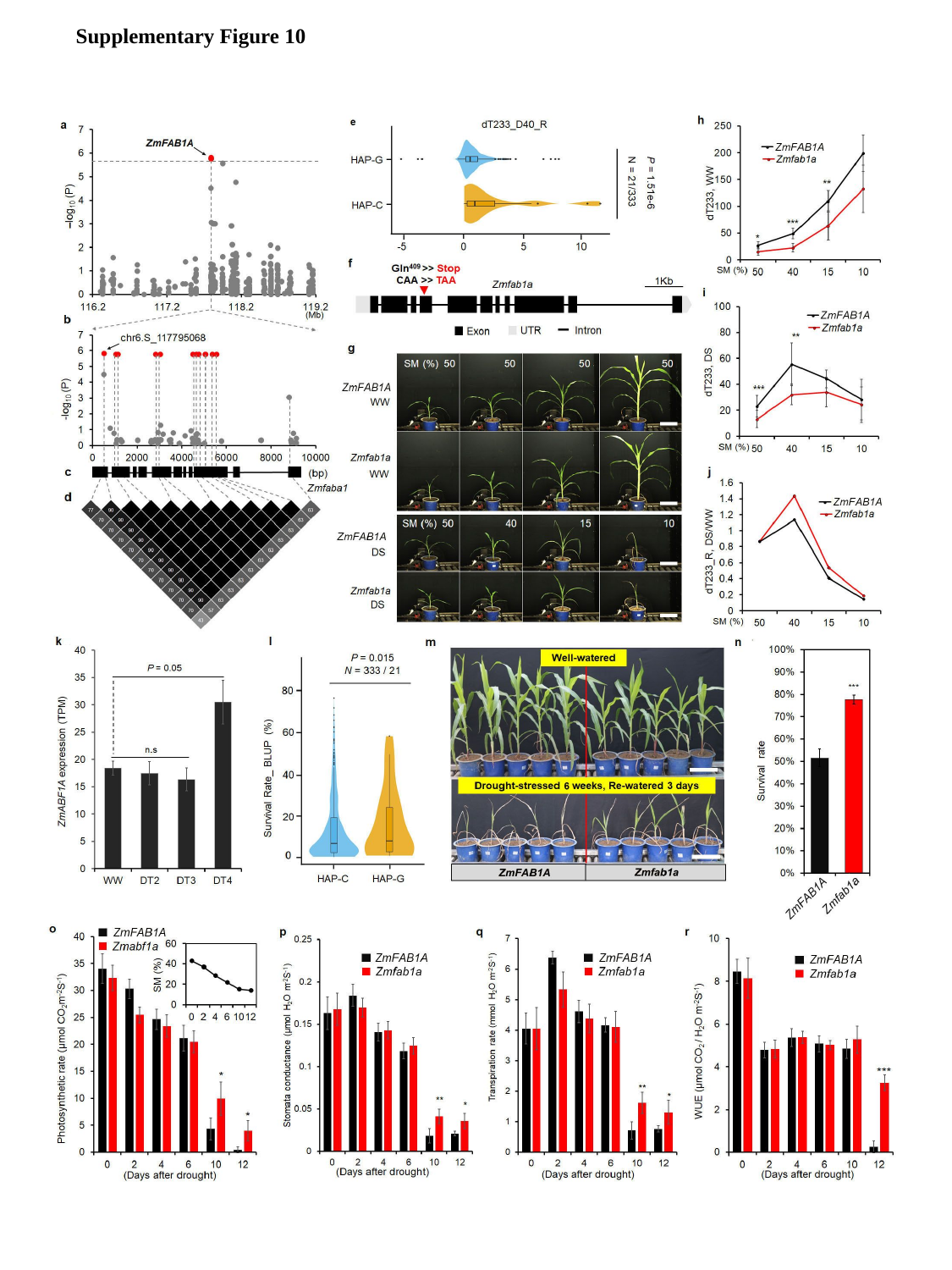

Supplementary Figure 10

## Slide 14
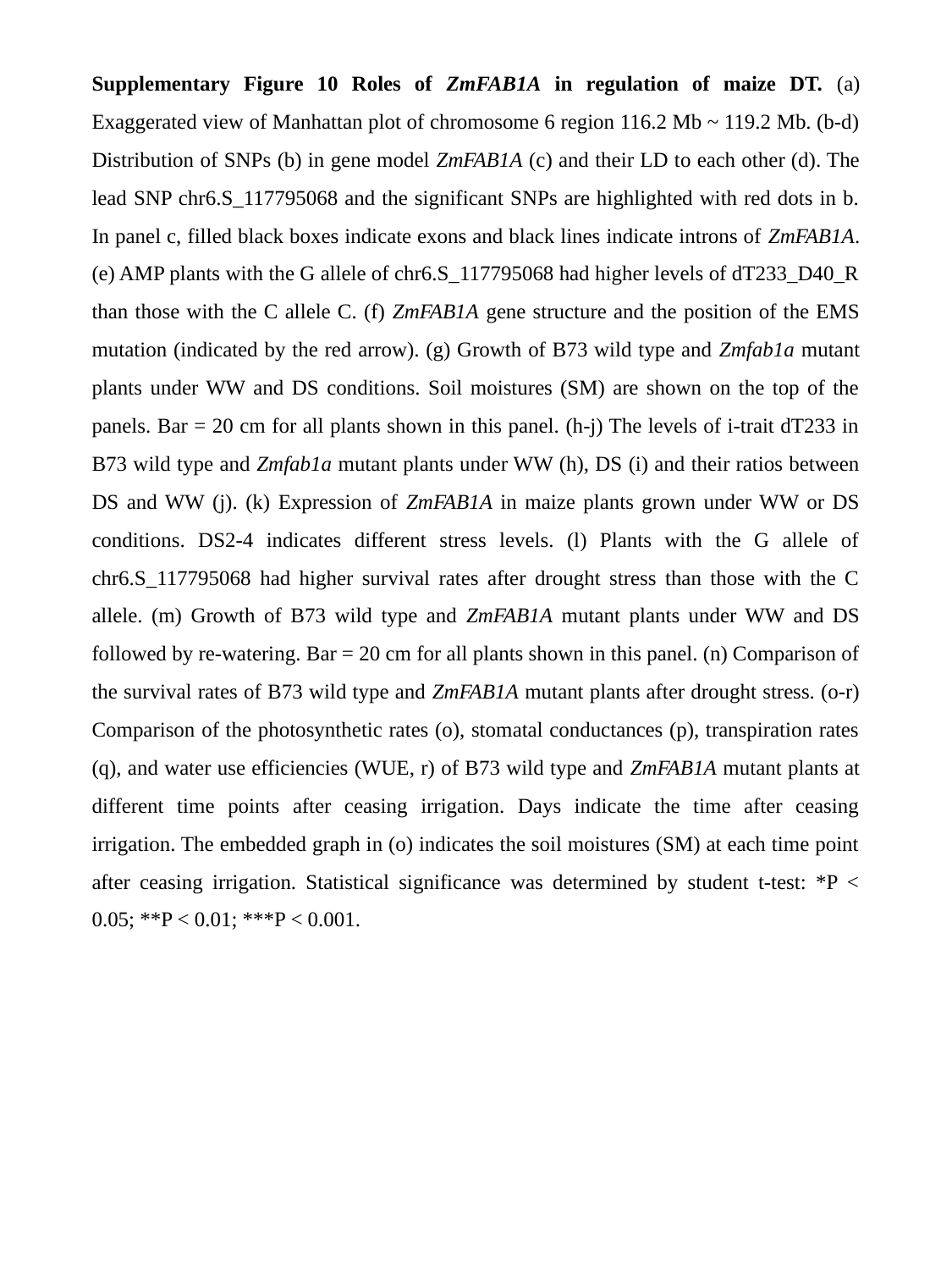

Supplementary Figure 10 Roles of ZmFAB1A in regulation of maize DT. (a) Exaggerated view of Manhattan plot of chromosome 6 region 116.2 Mb ~ 119.2 Mb. (b-d) Distribution of SNPs (b) in gene model ZmFAB1A (c) and their LD to each other (d). The lead SNP chr6.S_117795068 and the significant SNPs are highlighted with red dots in b. In panel c, filled black boxes indicate exons and black lines indicate introns of ZmFAB1A. (e) AMP plants with the G allele of chr6.S_117795068 had higher levels of dT233_D40_R than those with the C allele C. (f) ZmFAB1A gene structure and the position of the EMS mutation (indicated by the red arrow). (g) Growth of B73 wild type and Zmfab1a mutant plants under WW and DS conditions. Soil moistures (SM) are shown on the top of the panels. Bar = 20 cm for all plants shown in this panel. (h-j) The levels of i-trait dT233 in B73 wild type and Zmfab1a mutant plants under WW (h), DS (i) and their ratios between DS and WW (j). (k) Expression of ZmFAB1A in maize plants grown under WW or DS conditions. DS2-4 indicates different stress levels. (l) Plants with the G allele of chr6.S_117795068 had higher survival rates after drought stress than those with the C allele. (m) Growth of B73 wild type and ZmFAB1A mutant plants under WW and DS followed by re-watering. Bar = 20 cm for all plants shown in this panel. (n) Comparison of the survival rates of B73 wild type and ZmFAB1A mutant plants after drought stress. (o-r) Comparison of the photosynthetic rates (o), stomatal conductances (p), transpiration rates (q), and water use efficiencies (WUE, r) of B73 wild type and ZmFAB1A mutant plants at different time points after ceasing irrigation. Days indicate the time after ceasing irrigation. The embedded graph in (o) indicates the soil moistures (SM) at each time point after ceasing irrigation. Statistical significance was determined by student t-test: *P < 0.05; **P < 0.01; ***P < 0.001.

## Slide 15
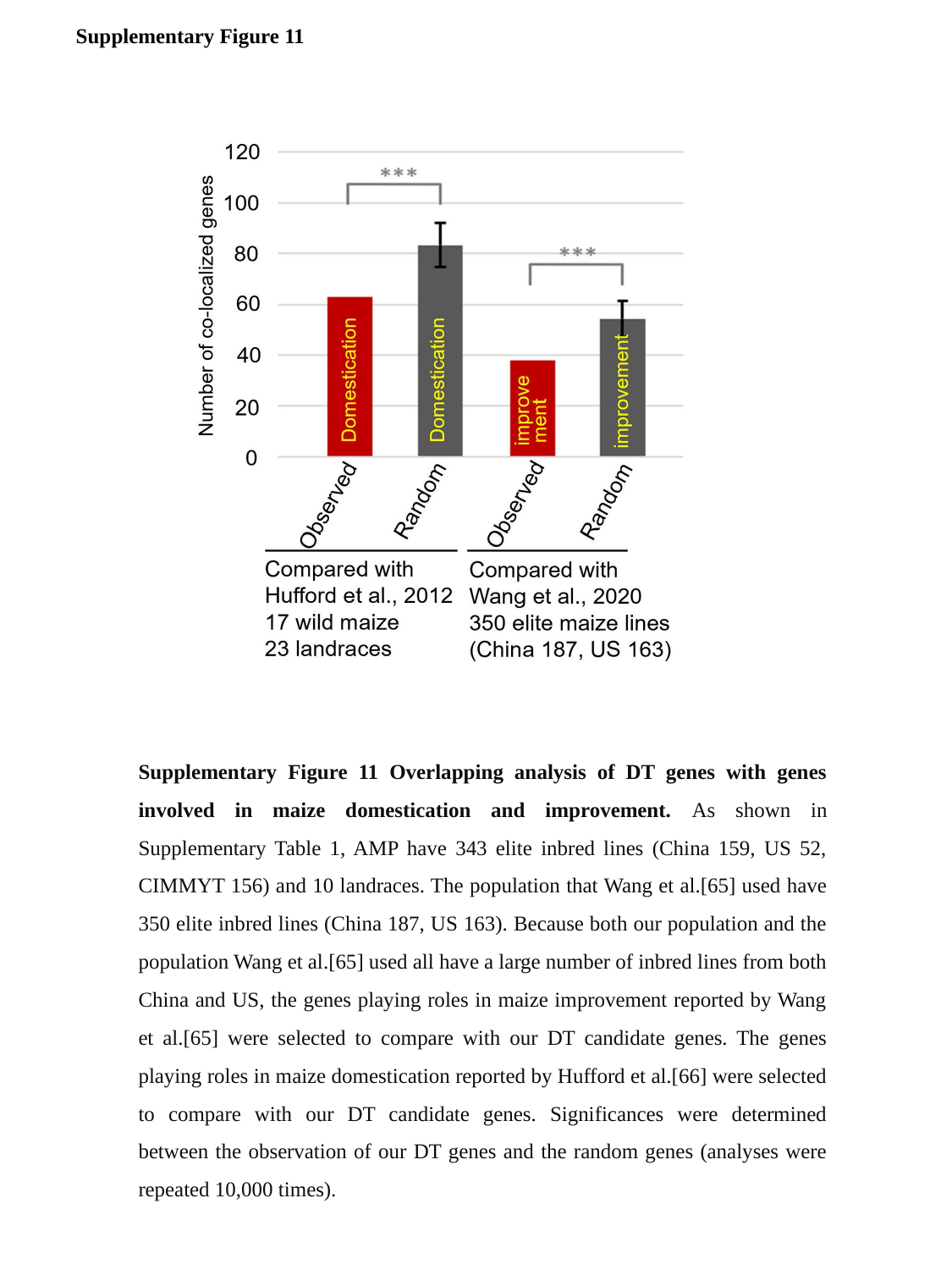

Supplementary Figure 11
Supplementary Figure 11 Overlapping analysis of DT genes with genes involved in maize domestication and improvement. As shown in Supplementary Table 1, AMP have 343 elite inbred lines (China 159, US 52, CIMMYT 156) and 10 landraces. The population that Wang et al.[65] used have 350 elite inbred lines (China 187, US 163). Because both our population and the population Wang et al.[65] used all have a large number of inbred lines from both China and US, the genes playing roles in maize improvement reported by Wang et al.[65] were selected to compare with our DT candidate genes. The genes playing roles in maize domestication reported by Hufford et al.[66] were selected to compare with our DT candidate genes. Significances were determined between the observation of our DT genes and the random genes (analyses were repeated 10,000 times).
