# Supplementary material for: Using high-throughput multiple optical phenotyping to decipher the genetic architecture of maize drought tolerance
Source: Genome Biol. 2021 Jun 24;22:185. doi: 10.1186/s13059-021-02377-0 (PMC8223302; doi:10.1186/s13059-021-02377-0)
Supplement: Supplementary file 11 — Additional file 11. Review history. [file 13059_2021_2377_MOESM11_ESM.docx]

Review History

**First round of review**

**Reviewer 1**

**Are you able to assess all statistics in the manuscript, including the appropriateness of statistical tests used?**

Yes, and I have assessed the statistics in my report.

**Comments to author:**

Drought tolerance is an important trait in crops. This work uses high-throughput optical images for phenotyping and performs GWAS in 368 inbred maize lines. The paper provides many candidate genes for follow-up functional studies on maize drought tolerance, which will be useful for maize community. However, I have several concerns on the novelty and quality of the work.

1. The image-based phenotyping method is not novel. The authors have used the same method in rice drought tolerance GWAS (see Guo et al., Mol Plant, 2018). The method is not newly developed in this work, and the descriptions should be shortened in the main text.

2. I'm not clear how the candidate genes are determined. If simply picking the nearest genes based on GWAS peaks, there must be many false positives. I have the same concerns for network analysis and GO analysis, since both of them rely on "correct" genes.

3. Functional reports of PGM2 and FAB1A in Arabidopsis should be mentioned. Moreover, what about the causative variants for the two genes? PCR-based sequencing could be performed. Furthermore, any differences in phenotypes between natural weak-function alleles and loss-of-function mutants?

4. Many important QTLs underlying drought tolerance may be of rare allele, and cannot be discovered in 368 inbred maize lines. Discussions may be needed.

5. Fig 2 and 3 could be moved to Supplementary Information. A list of candidate genes could be provided as a main text table.

**Reviewer 2**

**Are you able to assess all statistics in the manuscript, including the appropriateness of statistical tests used?**

Yes, and I have assessed the statistics in my report.

**Comments to author:**

The authors present a very interesting and huge study looking at high-resolution digital phenotyping of maize under well-watered and drought stress conditions.  The scope of the study and the datasets is really impressive.  The authors find and validate some interesting associations for drought response connecting the digital (e.g. hyperspectral measurements) to known physiological drought response.  However, some critical limitations of the study are that the methods and the overall work is poorly documents, methods are lacking, and it is difficult to read and understand the story.  The methods need much better details to understand what has been done in the data analysis, and thus understand the results.  The authors also need to pay close attention to 'drought response' vs 'drought tolerance'.  With a minor exception in the evaluation of mutant isolines, the study is not actually looking at drought tolerance per se.  There are also some serious questions on the multiple testing issues around doing GWAS for thousands of traits.  Overall, it is a very interesting study, with some solid validation of the GWAS, but needs some serious work on the writing, analysis and presentation.

L32 - need to define terms before using acronyms

L33 - what are 'i-traits'

L47 - the identified genes are causal or associated?

L64 - this sentence does not make sense.  'limited duration' ??

L80 - saying that GWAS has not been applied to understand the genetic architecture of DT is an overstatement, particularly in view of the previously cited studies.  The high-throughput phenotyping is not needed per se to understand the genetic architecture.  This has already been described.

L83 - 'Huge number' is incorrect grammar and also non-descriptive (vague)

L88 - how are you differentiating RGB vs HSI and CT?  The HSI and CT are just as external as the RGB.

The number of i-traits is not clearly being defined or distinguished.  Taking the same trait under two different treatments, or at two different time points, does NOT make a different trait, but just another measurement of the same trait.  This needs to be clarified in the methods and throughout the manuscript.  This combining of the same trait from different treatments and calling it different traits leads to lots of confusion in the rest of the manuscript.

L540 - is the heritability being calculated separately for each treatment?

L531 - '3sigma criterion' needs to be defined

The filtering of outliers presumes a normal distribution of the trait, which presumes a quantitative genetic architecture underlying the trait.  These are basically untested hypothesis.  Was the distribution and normal distribution of the traits examined?

L127 - TPA needs to be defined.

L129 - dT233 needs to be defined.

The study is comparing different i-traits to drought stress.  However, 'drought stress' has not actually been defined or measured?

L129 - the authors are claiming that a given trait is 'indicative of different levels of maize drought stress' but from the experimental design this appears to be nothing other than a difference in the given trait between the drought and the well-water treatment.  In no way, is this actually a measure of drought stress.

L144 - averaging the H2 of multiple different traits does not make any sense.  The mean is a statistical measure of multiple samples taken from a given population.  There is nothing presume that one trait should have any relation to another trait.

L153 - reporting the average effect of a significant SNP also doesn't make any sense.

L167 - if there are only 95 genes consistently associated with more than one i-trait, then this is telling me that there is a lot of noise in the system and the detection of SNP associations.

L558 - the experimental type I error for multiple testing markers is being controlled using something that sortof looks like a Bonferonni correction, but that correction should be the target threshold (e.g. 0.05) divided by the number of markers.  I can not actually find the number of markers used in the study reported here.  L571 reports 1.25 million SNPs.  If that is the correct number of SNPs tested in GWAS, then the calculation of the experimental p-value is not correct.

In addition to this, it must be recognized that there is actually huge number of multiple testing from running GWAS on 26,910 i-traits.  (assuming there are 555,000 markers used for GWAS)  This is actually resulting in 26,910 * 555,000 association tests (=1.49E10 total tests).  Using a bonferonni threshold for this many tests with an experimental alpha of 0.05 gives a GWAS threshold of 3.3E-12.

The selection of 'drought-related i-traits' is not clear.  The methods describe the use of multilayer perceptron, but this is not sufficiently described to understand the methods or repeat the analysis.  Further to this, the identification of these drought related traits is simply looking for traits that differentiate the response of the population under drought stress from the well water treatment.  Genetically determined drought tolerance per se, would actually be the RELATIVE DIFFERENCE of a given under well-watered vs drought treatment.  For the example of yield, if finding a genetic determinant of yield under drought conditions, then this would only be a 'drought QTL' if that genetic effect was not observed in well-watered conditions.  Otherwise (if the QTL is observed in both well-water and drought) then it is simply a QTL for yield and not drought.  This is something critical missing in this study.  It is not clear that the study is looking at the genetic response to drought stress, which is observed as a differential response.  In some of the results section the authors discuss the 'ratio of i-traits' which I find from the table S3 that this actually is the relative response of the given trait comparing drought treatment to well water treatment.  This is not described in the methods at all.  This needs to be much better clarified and brought to the front as this ratio is the variation in genetic drought response per se.

The analysis scripts and code used for processing and extraction of the i-traits needs to be included for reproducibility of this study.

The actual i-traits derived in this study is very opaque.  This needs much better detail including the exact parameters to derived the trait and the source code.  As such, this is not any sufficient documentation to understand what is a given trait.  I have reviewed Table S2 and S3-5, these tables include a general description of the trait, but nothing sufficient to understand what the trait is or how it was calculated.  For example TPA is 'Total Projected Area'.  But that is the only description.  Needs proper description of how it was calculated.  Further to this there are traits like T1 'The correlation' which is completely non-descriptive.

L224 - the description of something like lgA15_D334_WW in the text is completely useless.  This gives no understandable idea of what is the actual trait.

L241-246 - the analysis of eQTL for drought treatment response is interesting, but such it is currently based on unpublished RNA-seq data, which is unsuitable for including in this manuscript.  If running eQTL analysis, then need to include and publish the RNAseq dataset.

L285 - here again the authors are saying that the genes found in RESPONSE to drought are REGULATING drought tolerance.  It needs to be very clear that drought tolerance has NOT been assessed in this study.  Interestingly, some of the extracted traits that estimate biomass could serve as proxy for the relative growth rate and biomass and the difference (ratio) in biomass between drought and well water is drought tolerance.  These things are (would have been) very tractable particularly for the mutant isoline studies, were actual plant biomass and yield could have been studied in detail.

L311 - 324 - this analysis and interpretation of ZmcPGM2 is much better and the structure that the rest of the discussion is needing.  It is an actual understandable trait (compared to 'ddT200' - L287) that can translate into a physiological understanding of the plant.  This is an important aspect that is missing throughout.  Further to this the authors do a beautiful job of evaluation actual drought stress in the WUE and the ASI, which are shown to increase performance under drought stress.  This section on Zmcpgm2 is very solid.

The trait naming throughout the paper is difficult to read and makes the writing messy.  Things like "ddT200_D40_R" is great for R coding, but a mess to read in the text.  Need to figure out a better and more readable designation for the traits.

L359 - 'promoted' should be 'increased'

Fig. 6c-j - needs to be labeled with ZmcPGM2 and Zmcpgm2 as 'B73' is the background. Same for Figure 5

L947 - 'exaggerated' is the wrong word use 'zoom'  legend for Fig5a does not describe what is the i-trait for association

L949 - 'lead' is the wrong word.  I think what is meant is 'most significant'.

The application of GS using the identified genes vs a random set of the same number of genes is interesting approach to validate the association of this gene set with drought stress response, however this is of no practical value.  Simply a larger number of random genes is doing better than a small number of selected candidate genes.  The generation of high density marker data is cheap and easy, so actual implementation of GS will have huge number of markers (e.g. >>100K)

The use of RR-BLUP is really not the best model if expecting these identified candidate genes to have a larger effect on the trait.  With ridge regression all of the effects are shrunk to zero.  This is fine if having a large number of markers and most all of them are expected to have zero effect.  In this case the set of candidate genes are expected to ALL have an effect, so a model like BayesA would be more appropriate and allow all markers to have a relatively large effect.

L384-390 - the i-traits can not be used to 'predict' drought tolerance because they are actually measures of drought response, so the plants have to actually grown and these traits measured.  At that point it is no longer a 'prediction' but an 'observation'.

Including the equations on Fig 7 makes it very messy.  Should be inside the graph in small font, or not included.

The authors argue that the small effects of drought tolerance loci is a bottle neck to understand the genetic basis.  This is correct, and the traditional phenotyping if low-throughput limits the power of genetic studies which are determined by the SIZE of the population studies.  The authors have taken extremely high resolution phenotypes in this study but they have not actually increase the size of the population being studied.  In this respect the study does not actually increase the power for genetic mapping.

**Authors Response**

**Point-by-point responses to the reviewers’ comments:**

Reviewer reports:

Reviewer #1: Drought tolerance is an important trait in crops. This work uses high-throughput optical images for phenotyping and performs GWAS in 368 inbred maize lines. The paper provides many candidate genes for follow-up functional studies on maize drought tolerance, which will be useful for maize community.

*Reply: We wish to thank this reviewer for his/her support to our manuscript.*

However, I have several concerns on the novelty and quality of the work.

1. The image-based phenotyping method is not novel. The authors have used the same method in rice drought tolerance GWAS (see Guo et al., Mol Plant, 2018). The method is not newly developed in this work, and the descriptions should be shortened in the main text.

*Reply: In this work, we developed a high-throughput multiple optical imaging systems and the corresponding image analysis pipelines. To the best of our knowledge, very little information is available to combine CT, hyperspectral, RGB imaging system and GWAS to dissect the candidate genes of maize drought response. Notably, we successfully validated the roles of two candidate genes ZmcPGM2 and ZmFAB1A in regulation of i-traits and maize drought tolerance, which indicated the reliability of our strategy (using multiple optical imaging systems and GWAS) to dissect the genetic and molecular mechanisms of maize drought response.*

*1) We used the key words “hyperspectral”, “maize” and “genetic dissection” (or “genetic study” or “GWAS”) to search the published papers in Web of Science. The result showed that there were no records matching these key words (Reply Fig 1). To the best of our knowledge, very little information is available to use hyperspectral imaging technology and GWAS to decipher the genetic basis of maize stress responses.*

*2) We next used the key words “CT” (or Computed Tomography), “maize” and “genetic dissection” (or “genetic study” or “GWAS”) to search the published papers in Web of Science. The result showed that there were no records matching these key words (Reply Fig 2). To the best of our knowledge, very little information is available to use CT technology and GWAS to decipher the genetic basis of stress responses.*

*3) In our previous work (Guo et al., 2018), we used RGB image-based traits and GWAS to study drought resistance in rice. In this paper, we developed an integrated multiple optical phenotyping (CT, hyperspectral and RGB imaging) and specific image analysis pipelines to extract the multiple optical image traits, which could be combined with GWAS to dissect the genetic basis of maize drought response. Interestingly, we detected much more genetic loci by using i-traits collected with hyperspectral and CT imaging systems than those with RGB imaging system, which indicate that it is necessary and important to use multiple optical imaging strategy to dissect the genetic basis of crop complex traits, including drought response. Notably, we successfully validated the roles of two candidate genes ZmcPGM2 and ZmFAB1A in regulation of i-traits and maize drought tolerance, which indicated the reliability of our strategy (using multiple optical imaging systems and GWAS) to dissect the genetic and molecular mechanisms of maize drought response.*

*Reply Fig 1. Search the keywords“hyperspectral”, “maize” and “genetic dissection” at Web of Science.*

*Reply Fig 2. Search the key words “CT” (or Computed Tomography), “maize” and “genetic dissection” (or “genetic study” or “GWAS”) at Web of Science.*

*Reference*

*Guo Z, Yang W, Chang Y, Ma X, Tu H, Xiong F, Jiang N, Feng H, Huang C, Yang P, Zhao H, Chen G, Liu H, Luo L, Hu H, Liu Q, Xiong L. Genome-Wide Association Studies of Image Traits Reveal Genetic Architecture of Drought Resistance in Rice. Mol Plant 2018, 11:789-805.*

2. I'm not clear how the candidate genes are determined. If simply picking the nearest genes based on GWAS peaks, there must be many false positives. I have the same concerns for network analysis and GO analysis, since both of them rely on "correct" genes.

*Reply: We determined the candidate genes based on the GWAS peaks, that is, if a significant GWAS peak was in a gene, then this gene was determined a candidate gene.*

*We understand that for the self-pollinated plants, such as rice, there will be many false positive results using the GWAS peaks to determine the candidate genes, because the linkage disequilibrium (LD) decays slowly in these plants. However, maize is cross-pollinated plant species and exhibits extensive genetic variation, so much so that the average diversity at the nucleotide level between any two maize natural lines is higher than that between humans and chimps (Buckler and Stevens 2005). Moreover, the LD decays rapidly in diverse maize genotypes to the extent of 2–5 kb in the elite inbred lines (Yan et al. 2009). In the founders of NAM population, LD generally decays within 2 kb (Gore et al., 2009). These studies indicating that the association studies will generally define QTLs in small regions in such maize populations. The panel of 368 association mapping populations, which were used in this study, had been collected from tropical, subtropical and temperate regions around the world, and have broad genetic diversities. A previous study of this maize panel has suggested that the LD decays in 0.5kb in this diverse panel (Fu et al., 2013), therefore, the association mapping with this maize panel could reach single gene resolution. By using the same method as this study in determining the candidate genes with the same maize panel, previous studies have successfully cloned several maize drought tolerant genes, including ZmDREB2.7 (Liu et al., 2013), ZmNAC111(Mao et al., 2015), ZmVPP1 (Wang et al., 2016) and ZmPP2C-A10 (Xiang et al., 2017).*

*Although we could not absolutely exclude the false positives based on our method, the candidate genes, as a whole, could be reliable because 1) there were 25 previously-identified drought-tolerant genes were detected in our candidate gene set (Table S13), 2) association analysis with survival rate (SR, an index of drought tolerance) of this maize panel showed that there were more significant SNP-SR associations in the candidate genes than random simulation (Fig. 3d), 3) we successfully validated the roles of two candidate genes ZmcPGM2 and ZmFAB1A in regulation of i-traits and maize drought tolerance (Fig. 5 and 6; Fig. S10).*

*References:*

*(1) Buckler ES, Stevens NM: Maize Origins, Domestication, and selection. In: Motley TJ, Zerega N, Cross H (eds) Darwin’s Harvest. Columbia University Press, New York, 2005, pp 67–90.*

*(2) Fu J, Cheng Y, Linghu J, Yang X, Kang L, Zhang Z, Zhang J, He C, Du X, Peng Z: RNA sequencing reveals the complex regulatory network in the maize kernel. Nature Communications 2013, 4:2832.*

*(3) Gore MA, Chia JM, Elshire R, Sun Q, Ersoz E, Hurwitz BL, Peiffer JA, McMullen MD, Grills GS, Ross-Ibarra J, Ware DH, Buckler ES: A first-generation haplotype map of maize. Science 2009, 326:1115–1117.*

*(4) Liu S, Wang X, Wang H, Xin H, Yang X, Yan J, Li J, Tran LS, Shinozaki K, Yamaguchi-Shinozaki K, Qin F: Genome-wide analysis of ZmDREB genes and their association with natural variation in drought tolerance at seedling stage of Zea mays L. PLoS Genet 2013, 9:e1003790.*

*(5) Mao H, Wang H, Liu S, Li Z, Yang X, Yan J, Li J, Tran LS, Qin F: A transposable element in a NAC gene is associated with drought tolerance in maize seedlings. Nat Commun 2015, 6:8326.*

*(6) Wang X, Wang H, Liu S, Ferjani A, Li J, Yan J, Yang X, Qin F: Genetic variation in ZmVPP1 contributes to drought tolerance in maize seedlings. Nat Genet 2016, 48:1233-1241.*

*(7) Xiang Y, Sun X, Gao S, Qin F, Dai M: Deletion of an Endoplasmic Reticulum Stress Response Element in a ZmPP2C-A Gene Facilitates Drought Tolerance of Maize Seedlings. Mol Plant 2017, 10:456-469.*

*(8) Yan JB, Shah T, Warburton ML, Buckler ES, McMullen MD, Crouch J: Genetic characterization and linkage disequilibrium estimation of a global maize collection using SNP markers. PloS ONE 2009, 4:e8451*

3. Functional reports of PGM2 and FAB1A in Arabidopsis should be mentioned.

*Reply: We summarized the roles and PGM2 and FAB1A/B in Arabidopsis. Please see revised manuscript lines 296-298 (PGM2) and lines 371-373 (FAB1A/B).*

Moreover, what about the causative variants for the two genes? PCR-based sequencing could be performed.

*Reply: Thanks for the valuable suggestions of this reviewer. We performed PCR-based genomic DNA sequencing of ZmcPGM2 and ZmFAB1A in the maize populations (100 populations from the panel) as suggested. The sequenced fragments of ZmcPGM2 and ZmFAB1A include the putative promoters and 5’UTRs (-2000bp ~ +1 bp, base A in the start codon ATG is +1), the gene body regions from +1 to the stop codon (exons and introns), and the 3’UTRs (~500 bp). Although we identified many new variations from these two genes, but none of them was significantly associated with the corresponding i-traits or drought tolerant phenotype survival rate. We further analyzed the most significant SNPs (with i-traits or drought tolerance) and their tightly-linked SNPs, the putative causative variants were thus proposed and discussed. Please see revised manuscript lines 347-355 (ZmcPGM2) and 393-400 (ZmFAB1A).*

Furthermore, any differences in phenotypes between natural weak-function alleles and loss-of-function mutants?

*Reply: Many agronomic traits, including drought responses or tolerance, are very complex. The genetic backgrounds have great effects on these complex traits. Therefore, comparing the loss-of-function mutants (eg Zmcpgm2 and Zmfab1a) to their related wild type genotypes than to other genotypes (eg. natural weak alleles of the target genes with different genotypes) might be more conclusive for the functional interpretation of the target genes. Fortunately, in this study, B73, the mutation background of the loss of function mutants Zmcpgm2 and zmfab1a, is a weak allele of ZmcPGM2 (SNP chr5.S_10857363, C allele) or ZmFAB1A (SNP chr6.S_117795068, C allele) in the maize panel. Comparison between B73 and the mutants of Zmcpgm2 and zmfab1a suggested that the loss-of-function mutants had stronger drought tolerance than the weak allele B73 (please see Fig 6, Fig S10).*

4. Many important QTLs underlying drought tolerance may be of rare allele, and cannot be discovered in 368 inbred maize lines. Discussions may be needed.

*Reply: Thanks for the suggestions. We agree with this reviewer that rare allele could be also important for maize drought response and tolerance, and some of these alleles might not be detected in our maize panel. New populations and statistical approaches may need to be developed to detect these alleles. We discussed this part in the revised discussion section as suggested. Please see lines 483-489 of the revised manuscript.*

5. Fig 2 and 3 could be moved to Supplementary Information. A list of candidate genes could be provided as a main text table.

*Reply: Thanks for the suggestions of this reviewer. In our manuscript, there are seven figures in the main text. Fig.1 is mainly about the whole technical scheme. Fig. 2 is mainly about the overall description of the phenotypic data. Fig. 3 is about the description of the whole candidate genes. Fig. 4 is mainly about the description of the eQTLs that were associated with the expression of the candidate genes. Fig. 5 is about the roles of candidate gene ZmcPGM2 in regulation of i-traits and sugar biosynthesis. Fig. 6 is about the roles of ZmcPGM2 in regulation of maize drought tolerance, which is a case study. Fig.7 is mainly about the prediction results of maize drought tolerance by candidate genes and i-traits. These seven figures together suggested the main findings of a full story, therefore, it might be better to show them together as main figures for readers to better understand the story. A list of candidate genes in the main text could be helpful for readers to get useful information. However, we got 2318 candidate genes, which are too many to be put in the main text. A list of these candidate genes in supplemental information might be more suitable.*

Reviewer #2: REVIEW - GBIO-D-20-01281

The authors present a very interesting and huge study looking at high-resolution digital phenotyping of maize under well-watered and drought stress conditions. The scope of the study and the datasets is really impressive. The authors find and validate some interesting associations for drought response connecting the digital (e.g. hyperspectral measurements) to known physiological drought response.

*Reply: We wish to thank this reviewer for his/her support to our manuscript*

However, some critical limitations of the study are that the methods and the overall work is poorly documents, methods are lacking, and it is difficult to read and understand the story. The methods need much better details to understand what has been done in the data analysis, and thus understand the results. The authors also need to pay close attention to 'drought response' vs 'drought tolerance'. With a minor exception in the evaluation of mutant isolines, the study is not actually looking at drought tolerance per se. There are also some serious questions on the multiple testing issues around doing GWAS for thousands of traits. Overall, it is a very interesting study, with some solid validation of the GWAS, but needs some serious work on the writing, analysis and presentation.

*Reply: We appreciate the valuable suggestions from this reviewer. We carefully evaluated these suggestions and comments, and revised the manuscript accordingly. Please see our point-to-point responses. To describe the more details of data analysis, we modified the Materials and Methods in main text and revised Note S1-S2 to show the definition and calculation of HSI, RGB and CT image-based traits. In addition, we added Video S5-S7 to show the procedures of original data preprocessing including filtering outliers, T-test, and MLP. More details of our methods could be found at Supplementary Notes and Videos:*

*Supplementary Notes*

*Note S1. Definition and calculation of HSI, RGB, and CT i-traits*

*Note S2. Operation guide for the HSI, CT, and RGB data processing programs*

*Supplementary Videos*

*Video S1. High-throughput phenotyping to extract image-based traits for maize drought tolerance study*

*Video S2. High-throughput hyperspectral images acquisition and analysis*

*Video S3. High-throughput RGB images acquisition and analysis*

*Video S4. High-throughput CT images acquisition and analysis*

*Video S5. Operation procedure of filtering outlier*

*Video S6. Operation procedure of T-test*

*Video S7. Operation procedure of Multi-layer perception (MLP)*

*Video S8. Description of database including original images, phenotypic data, and genotype data*

L32 - need to define terms before using acronyms

*Reply: We defined the terms as suggested. (Line 32 in the revise manuscript)*

L33 - what are 'i-traits' ?

*Reply: 'I-traits' are the image-based traits. These images include RGB, HSI and CT images, which are digitized and quantified based on the 368 maize populations grown under well-watered and drought-stressed conditions, therefore, can be treated as 'traits'. We defined the term 'i-traits' in the revised manuscript. (Lines 32-34 in the revise manuscript)*

L47 - the identified genes are causal or associated?

*Reply: Technically, they are associated genes as detected by GWAS, although we can validate some of them. We replaced the word 'causal' with 'associated' in the revised manuscript (line 50) to give more precise statement.*

L64 - this sentence does not make sense. 'limited duration' ?

*Reply: We removed the duration statement in the revised manuscript (line 67).*

L80 - saying that GWAS has not been applied to understand the genetic architecture of DT is an overstatement, particularly in view of the previously cited studies. The high-throughput phenotyping is not needed per se to understand the genetic architecture. This has already been described.

*Reply: We are sorry for the vague statement. We mean that combining both high-throughput phenotyping and GWAS has not been applied in maize drought response study. We revised the statement accordingly (Line 81 in the revise manuscript).*

L83 - 'Huge number' is incorrect grammar and also non-descriptive (vague)

*Reply: Thank this reviewer for his/her reminding. We replaced these words with “Many” in the revised manuscript (Line 85)*

L88 - how are you differentiating RGB vs HSI and CT? The HSI and CT are just as external as the RGB.

*Reply: RGB contains three channels: red, green, and blue, which are mainly used to extract color-related and morphological-related traits, which could be used to mainly reflect “external characteristics”. Hyperspectral imaging (HSI) is mainly used to extract “internal” physiological and biochemical related traits. X-ray computed tomography (CT) can penetrate samples, which are mainly used to extract internal structure related traits.*

The number of i-traits is not clearly being defined or distinguished. Taking the same trait under two different treatments, or at two different time points, does NOT make a different trait, but just another measurement of the same trait. This needs to be clarified in the methods and throughout the manuscript. This combining of the same trait from different treatments and calling it different traits leads to lots of confusion in the rest of the manuscript.

*Reply: We agree with the reviewer’s comment. However, different loci could be detected associated with the same i-trait under different growth conditions or at two different time points. Moreover, treating the same i-traits under two different treatments, or at two different time points as different i-traits is helpful to facilitate the statistical analysis of phenotypic data and GWAS. Actually, some other studies also have adopted the same way as ours in definition of i-traits (Chen et al., 2018; Guo et al., 2018; Knoch et al., 2019)*

*References:*

*(1) Chen D, Shi R, Pape JM, Neumann K, Arend D, Graner A, Chen M, Klukas C. Predicting plant biomass accumulation from image-derived parameters. Gigascience. 2018, 7:1-13.*

*(2) Guo Z, Yang W, Chang Y, Ma X, Tu H, Xiong F, Jiang N, Feng H, Huang C, Yang P, Zhao H, Chen G, Liu H, Luo L, Hu H, Liu Q, Xiong L. Genome-Wide Association Studies of Image Traits Reveal Genetic Architecture of Drought Resistance in Rice. Mol Plant 2018, 11:789-805.*

*(3) Knoch D, Abbadi A, Grandke F, Meyer RC, Samans B, Werner CR, Snowdon RJ, Altmann T. Strong temporal dynamics of QTL action on plant growth progression revealed through high-throughput phenotyping in canola. Plant Biotechnol J. 2020, 18:68-82.*

L540 - is the heritability being calculated separately for each treatment?

*Reply: Yes, we calculated the heritability of each i-trait separately for each treatment (WW and DS).*

L531 - '3sigma criterion' needs to be defined

*Reply: We defined 3σCriterion in the revised manuscript. Basically, PauTa Criterion or 3σCriterion is to take the given confidence probability 99.7 % as the standard and the triple standard deviation limit as the basis. Any error exceeding this limit is considered not to belonging to the category of random error, but to the gross error. The measurement value with gross error is called abnormal value, which is undesirable and should be eliminated from the measurement data (Lines 582-587 in the revised manuscript).*

The filtering of outliers presumes a normal distribution of the trait, which presumes a quantitative genetic architecture underlying the trait. These are basically untested hypothesis. Was the distribution and normal distribution of the traits examined?

*Reply: We examined the distribution of the data after filtering of outliers. Interestingly, we found some of these i-traits did not show normal distribution in the maize panel, but shew a skewed distribution. This distribution pattern is different from some other directly-measurable agronomic traits, such as plant height, flowering time, grain yield etc., whose distribution patterns are usually normal. It has been reported by us and other groups that the distribution of some indirectly-measurable stress responsive traits, including maize drought tolerance and salt resistance, also show skewed distribution (Wang et al., 2016; Luo et al., 2018), in line with the distribution patterns of some i-traits detected in this study. Even the distribution is skewed, the quantitative genetic loci underlying these drought tolerance and salt resistance traits have been revealed (Wang et al., 2016; Luo et al., 2018). For some of the i-traits showing normal distribution or skewed distribution patterns, we could perform GWAS to detect significant associations, with normal Q-Q plots (Reply Fig 3).*

*Reply Fig 3. GWAS of exampled i-traits with normal or skewed distribution. a and d. normal distribution of i-trait lgA186_D46_R (a) and skewed distribution of Culm_diameter_700_D98_R (d). b and e. manhhattan plots of lgA186_D46_R (b) and Culm_diameter_700_D98_R based GWAS (e). c and f. Q-Q plots of lgA186_D46_R (c) and Culm_diameter_700_D98_R based GWAS (f).*

*Reference*

*(1) Wang X, Wang H, Liu S, Ferjani A, Li J, Yan J, Yang X, Qin F: Genetic variation in ZmVPP1 contributes to drought tolerance in maize seedlings. Nat Genet 2016, 48:1233-1241.*

*(2) Luo X, Wang B, Gao S, Zhang F, Terzaghi W, Dai M. Genome-wide association study dissects the genetic bases of salt tolerance in maize seedlings. J Integr Plant Biol 2019, 61:658-674.*

L127 - TPA needs to be defined.

*Reply: We defined the TPA as suggested. Please see revised manuscript line 130.*

L129 - dT233 needs to be defined.

*Reply: dT233: The first-order derivative of the total reflectance of the plant under different wavelength (233:959 nm), please see Supplemental Note 1 for more details. We defined dT233 in the revised manuscript as suggested, please see line 133 in revised manuscript.*

The study is comparing different i-traits to drought stress. However, 'drought stress' has not actually been defined or measured?

*Reply: We wish to thank this reviewer for his/her careful reading and valuable suggestions. We are sorry not to give clear explanation of 'drought stress' in the manuscript. As suggested, we explained these two growth conditions in the revised manuscript (lines 542-545). The 'drought stress' indicates the growth conditions without watering after the initial irrigation, while 'well-watered' indicates the normal watering during the growth of the maize plants. The term 'drought stress' is relative to the term 'well-watered' in this study, with the soil moistures of 'drought stress' conditions dropping from 50% to ~10%, while with the soil moistures of 'well-watered' conditions keeping at ~50%. Therefore, the stressed levels for the drought-stressed plants could be from no stress, to slight-level, middle-level and severe-level stresses. We measured the soil moistures at each time point during the maize growth. Please refer to Fig 1a in the manuscript and the Reply Fig 4 shown below. We could also see significantly impaired growth of the maize plants from middle-level to severe-level drought stresses (eg. from D43-D52. Fig 1a, middle panel).*

*Reply Fig 4. Soil moistures of the drought-stressed and well-watered growth conditions (original data).*

L129 - the authors are claiming that a given trait is 'indicative of different levels of maize drought stress' but from the experimental design this appears to be nothing other than a difference in the given trait between the drought and the well-water treatment. In no way, is this actually a measure of drought stress.

*Reply: Drought impaired the plant photosynthesis, thus to reduce plant biomass. In rice, TPA has been reported to indicate the growth of rice in drought stress experiment (Guo et al., 2018). We changed our statement from 'indicative of different levels of maize drought stress' into 'indicative of different growth situations of maize under various drought stresses'. Please see the revised manuscript (lines 132-133).*

*Reference*

*Guo Z, Yang W, Chang Y, Ma X, Tu H, Xiong F, Jiang N, Feng H, Huang C, Yang P, Zhao H, Chen G, Liu H, Luo L, Hu H, Liu Q, Xiong L. Genome-Wide Association Studies of Image Traits Reveal Genetic Architecture of Drought Resistance in Rice. Mol Plant 2018, 11:789-805.*

L144 - averaging the H2 of multiple different traits does not make any sense. The mean is a statistical measure of multiple samples taken from a given population. There is nothing presume that one trait should have any relation to another trait.

*Reply: We agree the notion that one trait may be not related to another. Therefore, we changed the average H2 (0.35) into middle number of H2 (0.4) in the revised manuscript to give a general information of H2 distribution. (Line 148 in the revised manuscript)*

L153 - reporting the average effect of a significant SNP also doesn't make any sense.

*Reply: We changed our statement by omitting the average effect of a significant SNP. (Line 156 in the revised manuscript)*

L167 - if there are only 95 genes consistently associated with more than one i-trait, then this is telling me that there is a lot of noise in the system and the detection of SNP associations.

*Reply: We used a mixed linear model controlling population structure (MLM, Q+K) in GWAS, which usually is more strict than general linear model (GLM) in GWAS to reduce noise. We detected few genes consistently associated with more than one i-trait with MLM model, which could indicate the differential genetic control of RGB, HSI and CT-derived i-traits. This might also suggest the importance and necessity to use multiple optical phenotyping to study crop complex agronomic traits.*

L558 - the experimental type I error for multiple testing markers is being controlled using something that sort of looks like a Bonferonni correction, but that correction should be the target threshold (e.g. 0.05) divided by the number of markers. I can not actually find the number of markers used in the study reported here. L571 reports 1.25 million SNPs. If that is the correct number of SNPs tested in GWAS, then the calculation of the experimental p-value is not correct. In addition to this, it must be recognized that there is actually huge number of multiple testing from running GWAS on 26,910 i-traits. (assuming there are 555,000 markers used for GWAS) This is actually resulting in 26,910 * 555,000 association tests (=1.49E10 total tests). Using a Bonferroni threshold for this many tests with an experimental alpha of 0.05 gives a GWAS threshold of 3.3E-12.

*Reply: We wish to thank this reviewer for his/her careful reading to our manuscript, and are sorry not giving clearer information. We added more information of these SNP markers in the revised manuscript (line 612). The SNP markers for the 368 maize populations are 558,650, which has been obtained by RNA sequencing to these populations (Fu et al., 2013). The SNP markers used for eQTL analysis are 1.25 million as published previously by GBS and RNA sequencing for a panel consisting of 540 maize inbred lines (Liu et al., 2017) (Line 624-626). In another separate project, we did RNA sequencing to 197 of the 540 maize inbred lines grown under DS and WW conditions. Of the 197 inbred lines, 135 lines are also belonging to the 368 maize populations used in this study (line 626-627).*

*It is true that stricter threshold (eg. Bonferroni correction) could reduce false positive results. However, it could also cause the loss of false negative results, especially when there is linkage among many SNP markers, which results in the non-independence of these SNPs. Taking into consideration of the linkage among the SNP makers of the same 368 maize populations, a previous GWAS study of these populations in drought response used a threshold of P < 1 × 10-5 to control type I error (Wang et al., 2016). To balance the non-independence of the SNP markers and the Bonferroni correction, we used the threshold 1/n (n = total markers used) in GWAS in this study, which has been also used in previous GWAS and eQTL studies (Wen et al., 2014; Liu et al., 2017).*

*Notably, we proved the reliability of the candidate genes from i-trait-based GWAS with multiple assays: 1) there were 25 previously-identified drought-tolerant genes were detected in our candidate gene set (Table S13), 2) association analysis with survival rate (SR) of this maize panel showed that there were more significant SNP-SR associations in candidate genes than random simulation (Fig. 3d), 3) we successfully validated the roles of two candidate genes ZmcPGM2 and ZmFAB1A in regulation of i-traits and maize drought tolerance (Fig. 5 and 6; Fig. S9).*

*References*

*(1) Fu J, Cheng Y, Linghu J, Yang X, Kang L, Zhang Z, Zhang J, He C, Du X, Peng Z: RNA sequencing reveals the complex regulatory network in the maize kernel. Nature Communications 2013, 4:2832.*

*(2) Liu H, Luo X, Niu L, Xiao Y, Chen L, Liu J, Wang X, Jin M, Li W, Zhang Q: Distant eQTLs and Non-coding Sequences Play Critical Roles in Regulating Gene Expression and Quantitative Trait Variation in Maize. Molecular Plant 2017, 10:414-426.*

*(3) Wang X, Wang H, Liu S, Ferjani A, Li J, Yan J, Yang X, Qin F: Genetic variation in ZmVPP1 contributes to drought tolerance in maize seedlings. Nature Genetics 2016, 48:1233-1241.*

*(4) Wen W, Li D, Li X, Gao Y, Li W, Li H, Liu J, Liu H, Chen W, Luo J, Yan J: Metabolome-based genome-wide association study of maize kernel leads to novel biochemical insights. Nature Communications 2014, 5:3438.*

The selection of 'drought-related i-traits' is not clear. The methods describe the use of multilayer perceptron, but this is not sufficiently described to understand the methods or repeat the analysis. Further to this, the identification of these drought related traits is simply looking for traits that differentiate the response of the population under drought stress from the well water treatment. Genetically determined drought tolerance per se, would actually be the RELATIVE DIFFERENCE of a given under well-watered vs drought treatment. For the example of yield, if finding a genetic determinant of yield under drought conditions, then this would only be a 'drought QTL' if that genetic effect was not observed in well-watered conditions. Otherwise (if the QTL is observed in both well-water and drought) then it is simply a QTL for yield and not drought. This is something critical missing in this study. It is not clear that the study is looking at the genetic response to drought stress, which is observed as a differential response. In some of the results section the authors discuss the 'ratio of i-traits' which I find from the table S3 that this actually is the relative response of the given trait comparing drought treatment to well water treatment. This is not described in the methods at all. This needs to be much better clarified and brought to the front as this ratio is the variation in genetic drought response per se.

*Reply: “Selection of drought-related i-traits” was changed into “Selection of drought responsive i-traits” (line 594 in the revised manuscript). And we added Video S5-S7 to show the procedures of original data preprocessing including filtering outliers, T-test, and MLP. After data preprocessing, the i-traits that have small difference under drought and well-watered conditions were removed, and largely reduced the complexity of consequent GWAS analysis. Notably, the selected drought responsive i-traits were not always associated the functional genes.*

The analysis scripts and code used for processing and extraction of the i-traits needs to be included for reproducibility of this study.

*Reply: The selected RGB, HSI, CT i-traits are listed in Table S3-S5. 4,322 significant SNPs and 2,318 candidate genes associated with i-traits are listed in Table S7. All of the original images, phenotypic data, and genotype data are publicly available for reuse with the link: http://plantphenomics.hzau.edu.cn/search_maize.action. The code of image analysis pipeline could be downloaded via the link: http://plantphenomics.hzau.edu.cn/download_checkiflogin_en.action. The operation guide for the HSI, CT, and RGB image analysis and data preprocessing can be obtained in the Video S2-S7, respectively. If further information is required for running the programs (such as calling the dynamic link library, dll files), please contact Dr. Wanneng Yang (ywn@mail.hzau.edu.cn). We also added the guidelines in Data availability.*

The actual i-traits derived in this study is very opaque. This needs much better detail including the exact parameters to derived the trait and the source code. As such, this is not any sufficient documentation to understand what is a given trait. I have reviewed Table S2 and S3-5, these tables include a general description of the trait, but nothing sufficient to understand what the trait is or how it was calculated. For example TPA is 'Total Projected Area'. But that is the only description. Needs proper description of how it was calculated. Further to this there are traits like T1 'The correlation' which is completely non-descriptive.

*Reply: We defined all the terms and abbreviations when first appeared in the main text. It is difficult to describe the detailed definition and calculation of all i-traits in the main text, thus, the detailed descriptions were shown into the Note S1 and Table S2.*

L224 - the description of something like lgA15_D334_WW in the text is completely useless. This gives no understandable idea of what is the actual trait.

*Reply: We defined all the terms and abbreviations when first appeared in the main text. (Lines 130, 133, 210, 228, 300, 328, 375 in the revised manuscript)*

L241-246 - the analysis of eQTL for drought treatment response is interesting, but such it is currently based on unpublished RNA-seq data, which is unsuitable for including in this manuscript. If running eQTL analysis, then need to include and publish the RNAseq dataset.

*Reply: We are sorry not providing the RNA-seq source data in this manuscript as they are under review in a separate manuscript, which is about the genomic basis of the balance between environmental adaptation and yield. However, the source RNA-seq data are temporally accessible at https://bigd.big.ac.cn/gsa/s/72gW6di4, and will be published with that manuscript.*

L285 - here again the authors are saying that the genes found in RESPONSE to drought are REGULATING drought tolerance. It needs to be very clear that drought tolerance has NOT been assessed in this study. Interestingly, some of the extracted traits that estimate biomass could serve as proxy for the relative growth rate and biomass and the difference (ratio) in biomass between drought and well water is drought tolerance. These things are (would have been) very tractable particularly for the mutant isoline studies, were actual plant biomass and yield could have been studied in detail.

*Reply: Although a “drought responsive” gene is not necessary a gene regulating drought tolerance, many genes regulating plant drought tolerance are usually drought responsive. The well-known cases are from the genes involved in ABA signaling. For example, the genes important for early ABA signal transduction, including PYLs, PP2CAs, SnRKs, are up- or down-regulated by drought stress (because of the drought responsive or ABA responsive elements in their promoters), suggesting the “response” of these genes to drought, however, mutation of or enhancing the expression of these genes have changed the plant drought tolerance (Fujita et al., 2009; Bhaskara et al., 2012; Zhao et al., 2016; Xiang et al., 2017; He et al., 2018), indicating that they can also “regulate” plant drought tolerance. There are several common indexes used to monitor plant “drought tolerance”, such as survival rate, biomass etc. We agree with the reviewer that biomass is an alternative indicator of plant drought tolerance, however, there are many researchers have used survival rate as an index in studying crop drought tolerance (Liu et al., 2013; Mao et al., 2015; Wang et al., 2016; Xiang et al., 2017; Guo et al., 2018). ZmcPGM2 and ZmFAB1A shew “response” to drought, because their expression levels were changed upon drought stress (Fig 6a, Fig S10k), but whether they regulate drought tolerance remains known. For the cases of ZmcPGM2 and ZmFAB1A in this study, we used survival rate to evaluate their roles in maize drought tolerance, and the results indicated that they play negative roles to regulate survival rates (drought tolerance) after drought stress (Fig 6, Fig S10).*

*References*

*(1) Fujita Y, Nakashima K, Yoshida T, Katagiri T, Kidokoro S, Kanamori N, Umezawa T, Fujita M, Maruyama K, Ishiyama K, Kobayashi M, Nakasone S, Yamada K, Ito T, Shinozaki K, Yamaguchi-Shinozaki K: Three SnRK2 protein kinases are the main positive regulators of abscisic acid signaling in response to water stress in Arabidopsis. Plant Cell Physiol. 2009, 50:2123-32.*

*(2) Bhaskara GB, Nguyen TT, Verslues PE. Unique drought resistance functions of the highly ABA-induced clade A protein phosphatase 2Cs. Plant Physiol. 2012, 160:379-95.*

*(3) Zhao Y, Chan Z, Gao J, Xing L, Cao M, Yu C, Hu Y, You J, Shi H, Zhu Y, Gong Y, Mu Z, Wang H, Deng X, Wang P, Bressan RA, Zhu JK. ABA receptor PYL9 promotes drought resistance and leaf senescence. Proc Natl Acad Sci U S A. 2016,113:1949-54.*

*(4) Xiang Y, Sun X, Gao S, Qin F, Dai M. Deletion of an Endoplasmic Reticulum Stress Response Element in a ZmPP2C-A Gene Facilitates Drought Tolerance of Maize Seedlings. Mol Plant. 2017,10:456-469.*

*(5) He Z, Zhong J, Sun X, Wang B, Terzaghi W, Dai M. The Maize ABA Receptors ZmPYL8, 9, and 12 Facilitate Plant Drought Resistance. Front Plant Sci. 2018, 9:422.*

L311 - 324 - this analysis and interpretation of ZmcPGM2 is much better and the structure that the rest of the discussion is needing. It is an actual understandable trait (compared to 'ddT200' - L287) that can translate into a physiological understanding of the plant. This is an important aspect that is missing throughout. Further to this the authors do a beautiful job of evaluation actual drought stress in the WUE and the ASI, which are shown to increase performance under drought stress. This section on Zmcpgm2 is very solid.

*Reply: We thank this reviewer for his/her support. In the revised manuscript, we tried our best to explain more clearly the i-traits to make them more understandable. Please see the revised manuscript.*

The trait naming throughout the paper is difficult to read and makes the writing messy. Things like "ddT200_D40_R" is great for R coding, but a mess to read in the text. Need to figure out a better and more readable designation for the traits.

*Reply: ddT200_D40_R is the ratio of second-order derivative of the 880 nm total reflectance under drought stress to second-order derivative of the 880 nm total reflectance under well-water at 40 days after sowing. We also defined all the terms and abbreviations when first appeared in the main text (Lines 130, 133, 210, 228, 300, 328, 375 in the revised manuscript)*

L359 - 'promoted' should be 'increased'

*Reply: We changed 'promoted' into 'increased' as suggested. Please see revised manuscript. (Line 390 in the revised manuscript)*

Fig. 6c-j - needs to be labeled with ZmcPGM2 and Zmcpgm2 as 'B73' is the background. Same for Figure 5

*Reply: We changed the label as suggested in Fig5 and 6 as suggested. Please see revised Fig 5 and 6*.

L947 - 'exaggerated' is the wrong word use 'zoom' legend for Fig5a does not describe what is the i-trait for association

*Reply: We changed the legend for Fig5a as suggested and the associated i-trait was given in the legend. (Line 1028-1030 in the revised manuscript)*

L949 - 'lead' is the wrong word. I think what is meant is 'most significant'.

*Reply: We replaced the word 'lead' in the legend for Fig5b with 'most significant'. (Line 1031 in the revised manuscript)*

The application of GS using the identified genes vs a random set of the same number of genes is interesting approach to validate the association of this gene set with drought stress response, however this is of no practical value. Simply a larger number of random genes is doing better than a small number of selected candidate genes. The generation of high density marker data is cheap and easy, so actual implementation of GS will have huge number of markers (e.g. >>100K)

*Reply: The approaches used to obtain markers for genome selection (GS) are usually chip identification and/or sequencing of a target crop, the cost in marker detection increases with the number of markers used in GS. With more often use of GS in breeding, people are generating more and more data sets for GS, which also increase the cost of data storage and analyses. We agree with this review that more genes (e.g. whole genomic genes or markers) will have better GS performs than a small number of selected candidate genes. However, if a small number of selected candidate genes can perform similarly or closely as a large number of selected random genes in GS, for example in our study, 30 candidate genes showed 80% (0.7/0.88) efficiency of the whole genomic genes in GS assays (please see revised Fig 7a), this will help us to reduce the cost in GS.*

The use of RR-BLUP is really not the best model if expecting these identified candidate genes to have a larger effect on the trait. With ridge regression all of the effects are shrunk to zero. This is fine if having a large number of markers and most all of them are expected to have zero effect. In this case the set of candidate genes are expected to ALL have an effect, so a model like BayesA would be more appropriate and allow all markers to have a relatively large effect.

*Reply: Thanks for the suggestion from this reviewer. We performed both RR-BLUP and BayesA with our data sets. Interestingly, there was no significant difference between the results from RR-BLUP and those from BayesA (please see revised Fig 7a). In case of markers or loci with large effects on traits, BayesA may perform better than RR-BLUP in GS. However, in our case, RR-BLUP and BayesA did not perform differently because the trait drought tolerance is usually controlled by many loci with small effects (Hu and Xiong, 2014).*

*Reference*

*Hu H, Xiong L: Genetic Engineering and Breeding of Drought-Resistant Crops. Annual Review of Plant Biology 2014, 65:715-741.*

L384-390 - the i-traits can not be used to 'predict' drought tolerance because they are actually measures of drought response, so the plants have to actually grown and these traits measured. At that point it is no longer a 'prediction' but an 'observation'.

*Reply: We changed the statement of 'prediction' into 'selection' (Line 440 in the revised manuscript). The significant association of the i-traits with survival rates (drought tolerance) of maize plants after drought stress indicated that these i-traits could be potential biomarker used for selection of maize germplasms.*

Including the equations on Fig 7 makes it very messy. Should be inside the graph in small font, or not included.

*Reply: Figure 7 has been modified as suggested.*

The authors argue that the small effects of drought tolerance loci is a bottle neck to understand the genetic basis. This is correct, and the traditional phenotyping if low-throughput limits the power of genetic studies which are determined by the SIZE of the population studies. The authors have taken extremely high resolution phenotypes in this study but they have not actually increase the size of the population being studied. In this respect the study does not actually increase the power for genetic mapping.

*Reply: We agree with this reviewer that population size is important to increase the power for genetic mapping. Drought tolerance is a very complex quantitative trait controlled by many loci with small effects. How to dissect this complex trait is a bottle neck for drought tolerance study. The traditional methods in drought response phenotyping are usually low-throughput, time-consuming and costly. Rather than increase the GWAS power, the advances of this study are that we developed a high-throughput multiple optical system to dissect drought responses into many pieces and investigated the genetic control of each piece, resulting in the identification of many novel drought responsive QTLs and candidate genes, which will be helpful for us to deep understand the genetic and molecular basis of crop drought tolerance. Moreover, this approach could be also suitable for studying the complex traits of other crops.*

**Second round of review**

**Reviewer 1**

The revision is improved. Especially, the causative genes were sequenced and screened for causative variation, although no strong candidates are detected. Some descriptions are also revised.
